# A randomized, open-label, parallel, multi-center Phase IV study to compare the efficacy and safety of atorvastatin 10 and 20 mg in high-risk Asian patients with hypercholesterolemia

**DOI:** 10.1371/journal.pone.0245481

**Published:** 2021-01-22

**Authors:** Ji Bak Kim, Woo Hyuk Song, Jong Sung Park, Tae-Jin Youn, Yong Hyun Park, Shin-Jae Kim, Sung Gyun Ahn, Joon-Hyung Doh, Yun-Hyeong Cho, Jin Won Kim

**Affiliations:** 1 Department of Medicine, Korea University Graduate School, Seoul, Korea; 2 Division of Cardiology, Department of Internal Medicine, Korea University Ansan Hospital, Ansan, Korea; 3 Department of Cardiology, Dong-A University Hospital, Busan, Korea; 4 Division of Cardiology, Department of Internal Medicine, College of Medicine, Seoul National University and Cardiovascular Center, Seoul National University Bundang Hospital, Seongnam, Korea; 5 Division of Cardiology, Department of Internal Medicine, Cardiovascular Center, Pusan National University Yangsan Hospital, Yangsan, Korea; 6 Department of Cardiology, Ulsan University Hospital, University of Ulsan College of Medicine, Ulsan, Korea; 7 Division of Cardiology, Department of Internal Medicine, Wonju Severance Christian Hospital, Wonju, Korea; 8 Department of Cardiology, Inje University Ilsan Paik Hospital, Goyang, Korea; 9 Department of Internal Medicine, Myongji Hospital, Goyang, Korea; 10 Cardiovascular Center, Korea University Guro Hospital, Seoul, Korea; Universita degli Studi di Milano, ITALY

## Abstract

**Background:**

Although accumulating evidence suggests a more extensive reduction of low-density lipoprotein cholesterol (LDL-C), it is unclear whether a higher statin dose is more effective and cost-effective in the Asian population. This study compared the efficacy, safety, and cost-effectiveness of atorvastatin 20 and 10 mg in high-risk Asian patients with hypercholesterolemia.

**Methods:**

A 12-week, open-label, parallel, multicenter, Phase IV randomized controlled trial was conducted at ten hospitals in the Republic of Korea between October 2017 and May 2019. High-risk patients with hypercholesterolemia, defined according to 2015 Korean guidelines for dyslipidemia management, were eligible to participate. We randomly assigned 250 patients at risk of atherosclerotic cardiovascular disease to receive 20 mg (n = 124) or 10 mg (n = 126) of atorvastatin. The primary endpoint was the difference in the mean percentage change in LDL-C levels from baseline after 12 weeks. Cost-effectiveness was measured as an exploratory endpoint.

**Results:**

LDL-C levels were reduced more significantly by atorvastatin 20 mg than by 10 mg after 12 weeks (42.4% vs. 33.5%, p < 0.0001). Significantly more patients achieved target LDL-C levels (<100 mg/dL for high-risk patients, <70 mg/dL for very high-risk patients) with atorvastatin 20 mg than with 10 mg (40.3% vs. 25.6%, p < 0.05). Apolipoprotein B decreased significantly with atorvastatin 20mg versus 10 mg (−36.2% vs. −29.9%, p < 0.05). Lipid ratios also showed greater improvement with atorvastatin 20 mg than with 10 mg (total cholesterol/high-density lipoprotein cholesterol ratio, −33.3% vs. −29.4%, p < 0.05; apolipoprotein B/apolipoprotein A1 ratio, −36.7% vs. −31.4%, p < 0.05). Atorvastatin 20 mg was more cost-effective than atorvastatin 10 mg in terms of both the average and incremental cost-effectiveness ratios. Safety and tolerability of atorvastatin 20 mg were comparable to those of atorvastatin 10 mg.

**Conclusion:**

In high-risk Asian patients with hypercholesterolemia, atorvastatin 20 mg was both efficacious in reducing LDL-C and cost-effective compared with atorvastatin 10 mg.

## Introduction

Dyslipidemia is one of the most critical risk factors for cardiovascular disease (CVD) [1, 2]. Therefore, lipid-lowering therapy is undeniably essential for reducing cardiovascular adverse events (AEs), especially in patients with risk factors or established CVD [3]. 3-Hydroxy-3-methylglutaryl-coenzyme A reductase inhibitors, known as statins, are the most effective and widely used drugs for treating dyslipidemia and have been the cornerstone medication for lipid-lowering therapy [4, 5]. Studies conducted during the past few decades have confirmed the effectiveness and safety of statins through several large-scale clinical trials [6–8]. Based on these studies, the latest international guidelines for dyslipidemia recommend increasingly intensive statin treatment to prevent CVD [1, 9]. However, the landmark statin trials were conducted in western countries. Therefore, some questions remain regarding statin treatment in the Asian population. Asians exhibit a higher response to statin treatment than Westerners [10, 11], and high-dose statin-related side effects are known to be more common in patients of Asian ethnicity due to variations in drug metabolism and clearance [12, 13]. In the same context, Health Canada and the US Food and Drug Administration refer to patients of Asian ethnicity as a higher risk group for statin-induced myopathy and recommend starting patients on lower statin doses [14, 15]. Therefore, there is still no clear information regarding the appropriate statin dose for Asian patients. Unlike the recommendations in the latest guidelines, low-dose statins are used widely and the achievement rate of target low-density lipoprotein cholesterol (LDL-C) levels and compliance with statin treatment are suboptimal in Asian countries [16–20].

Because lipid-lowering therapy using statin should continue throughout the patient’s life, it is important to select the appropriate dose of statin to maximize the effect and minimize the risk of side effects. Therefore, this study’s objective was to compare the efficacy and safety of atorvastatin 10 mg versus those of atorvastatin 20 mg in Asian patients with high CVD risk, defined according to 2015 Korean guidelines for managing dyslipidemia. Also, cost-effectiveness, another essential factor for long-term drug maintenance [21, 22], was analyzed.

## Methods

### Ethics statement

The study protocol was designed with sufficient consideration of patient safety in accordance with the recommendations of The Korea University Guro Hospital Institutional Review Board. The Korea University Guro Hospital Institutional Review Board approved the study protocol (KUGH17199-001). Written informed consent was obtained from all participants before their inclusion in the study.

### Study patients

Patients aged >19 years who had hypercholesterolemia with high CVD risk, according to the 2015 Korean guidelines for managing dyslipidemia, were eligible to participate in the study. Based on the guidelines, patients with carotid stenosis of >50%, abdominal aortic aneurysm, or diabetes were considered the high-risk group. Patients with coronary artery disease (CAD), ischemic stroke, transient ischemic attack (TIA), or peripheral arterial disease (PAD) were considered the very high-risk group. In case the patients were previously treated with lipid-lowering agents, a washout period was implemented (8 weeks for fenofibrate, four weeks for other lipid-lowering agents). If the patients were naïve to lipid-lowering agents or had already completed the washout period, a 1:1 randomization was performed when the following conditions were met: 1) LDL-C levels ≥100 mg/dL and triglyceride (TG) levels ≤500 mg/dL for high-risk patients, and 2) LDL-C levels ≥70 mg/dL and TG levels ≤500 mg/dL for very high-risk patients. Patients with uncontrolled diabetes mellitus (glycosylated hemoglobin, HbA1c, >9%, arbitrary threshold), uncontrolled hypertension (systolic blood pressure ≥180 mmHg or diastolic blood pressure ≥110 mmHg at screening), and thyroid dysfunction (thyroid-stimulating hormone ≥1.5 times the upper limit of normal) were excluded. Patients with severe renal insufficiency (serum creatinine level ≥2 times the upper limit of normal), active liver diseases (serum aspartate or alanine aminotransferase levels more than twice the upper limit of normal), known history of myopathy, or elevated creatinine phosphokinase level (more than twice the upper limit of normal) were excluded from the study. Further exclusion criteria are listed in **S1 Table**. Use of other statins, fibrates, niacin, bile acid sequestrants, oral steroids, anti-obesity drugs, fish oil, cholestin, fiber-based laxatives, phytosterol margarine, cyclosporine, macrolide, and antifungal drugs was not permitted during the study.

### Study design

**Fig 1:**
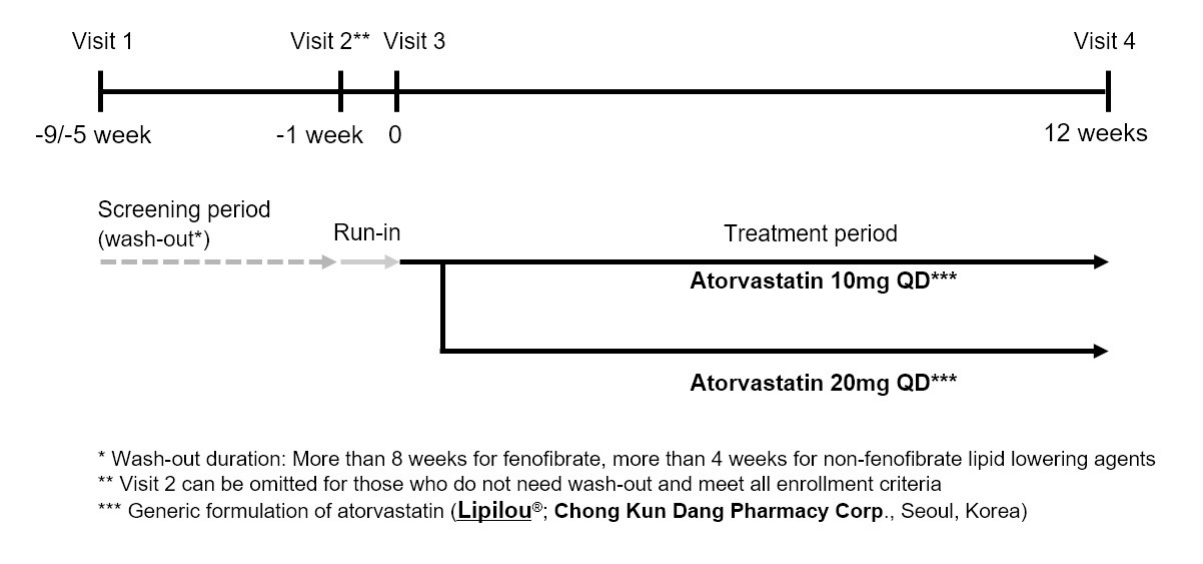
Study scheme of the study.

This study was a 12-week, open-label, parallel, multicenter, Phase IV randomized controlled trial conducted at ten hospitals in the Republic of Korea between October 2017 and May 2019 (**S2 Table**). The study protocol was designed with sufficient consideration of patient safety. The institutional review board (IRB) of each hospital approved the study protocol. The study was not registered before subject enrollment, as it was not a requirement of the IRB. This trial has since been registered with Clinicaltrials.gov (NCT04511000, Aug 2020). All participants or their legal guardians provided written informed consent before their inclusion in the study. This trial has the following three phases: (1) Screening period; (2) Run-in period; and (3) Treatment period (**Fig 1**). All blood tests were conducted in the central laboratory. At visit 1, eligible and consenting patients underwent tests for baseline assessment (**S3 Table**) and were instructed to make therapeutic lifestyle changes. Patients on a statin or another lipid-lowering therapy underwent a washout period of 8 weeks for fenofibrate and four weeks for other lipid-lowering agents, including statins. At visit 2, patients were tested for reevaluation to determine whether they still met the inclusion criteria. If the eligible patients were naïve to lipid-lowering agents or did not take lipid-lowering agents for a specified period at the screening examination (8 weeks for fenofibrate, four weeks for other lipid-lowering agents), visit two was omitted. At visit 3, the patients were randomly assigned to the atorvastatin 10 mg or atorvastatin 20 mg groups in a 1:1 ratio using SAS version 9.3 (SAS Institute, Inc, Cary, North Carolina). We used the stratified block randomization method and the patients entered the 12-week treatment period. We used a generic atorvastatin drug (Atorvastatin calcium anhydrous; Lipilou^®^; Chong Kun Dang Pharmacy Corp., Seoul, Korea) in this study. After the 12-week treatment period, we repeated all baseline measurements, conducted residual study drug retrieval, measured drug compliance, checked for concomitant medication, and evaluated adverse reactions.

### Endpoints and safety assessment

We conducted an efficacy analysis on full analysis (FA) sets and per-protocol (PP) sets and a safety analysis on the safety analysis set. The FA set included all participants who had taken at least one dose of the study drug and underwent at least one efficacy assessment. The PP set consisted of all patients in the FA set who completed the study without major protocol deviation. Subjects who did not complete the period specified in the clinical trial protocol, or had medication adherence of <80% or >120%, and deviated from the inclusion/exclusion criteria were excluded from the study. The safety set consisted of participants who had taken at least one dose of the study drug and underwent at least one safety-related interview.

The primary efficacy endpoint was comparing the mean percentage change in LDL-C levels between atorvastatin 10 and 20 mg groups after 12 weeks of treatment. The secondary endpoint was the mean percentage change from baseline in the following parameters: (1) lipid parameters including total cholesterol (TC), TG, high-density lipoprotein cholesterol (HDL-C), non-HDL-C, apolipoprotein (Apo)-B, and Apo-A1; (2) LDL-C/HDL-C ratio, TC/HDL-C ratio, non-HDL-C/HDL-C ratio, and Apo-B/Apo-A1 ratio; and (3) rate of achievement of target LDL-C and non-HDL-C levels according to the patient’s risk factors after 12 weeks of treatment. We also compared mean changes in fasting glucose and hemoglobin A1c (HbA1c) levels from baseline after 12 weeks of treatment. As an additional exploratory endpoint, we compared the cost-effectiveness between atorvastatin 10 and 20 mg in reducing the LDL-C levels. The reduction of LDL-C levels was defined as similar to that of the primary efficacy endpoint, i.e., the mean percentage change in LDL-C levels after 12 weeks of treatment. The cost-effective analysis results are presented as the average cost-effectiveness ratio (ACER) and the incremental cost-effectiveness ratio (ICER). The ACER was calculated by dividing the mean cost of each group by the mean percentage change in each group’s LDL-C levels. The ICER was calculated by dividing the difference in the mean cost of each group by the difference in the mean percentage change in each group’s LDL-C levels. The ACER implies the cost of reducing the LDL-C level for each alternative, and the lower the ACER, the better the cost-effectiveness. The ICER indicates the additional cost of further reducing the LDL-C level when comparing the two options.

Safety assessments consisted of monitoring and recording all laboratory tests, vital signs, electrocardiograms, AEs, serious AEs, and possible association of AEs with the study drug. Adverse drug reactions (ADRs) were defined as drug-related AEs and classified as certain, probable/likely, possible, unlikely, conditional/unclassified, unassessable/unclassifiable, or not applicable to the study drug. We evaluated laboratory AEs by comparing baseline laboratory values with those measured at follow-up. AE severity was classified as mild for mild symptoms or signs not affecting activities of daily living, moderate for minor limitations in daily living activities, and severe for significant limitations in daily living activities. The investigators at each center decided whether the patients with drug-related AEs should withdraw from the study.

### Statistical analysis

Data are expressed as mean, standard deviation (SD), median, minimum, and maximum values for continuous variables. The number and percentage of patients are presented for categorical variables. The normality of the data distribution as tested with the Shapiro-Wilk test, and homogeneity of variances was verified by F-test. Since the normality assumption was satisfied, we conducted independent two-sample t-test to compare continuous variables between two treatment groups and performed Pearson’s χ2 test or Fisher’s exact test to compare categorical variables between groups. Within each group, a paired t-test was used to compare the pre- and post-treatment measurements. All statistical analyses were two-sided, and p- values <0.05 were considered to be statistically significant. The SAS software package version 9.4 (SAS Institute Inc, Cary, North Carolina, USA) was used for all analyses.

### Sample size calculation

The sample size of the study was calculated according to the estimated mean percentage changes in LDL-C levels obtained from the US Food and Drug Administration approval data for rosuvastatin. Using this reference, we assumed that the difference in the mean percentage change in LDL-C levels between rosuvastatin 20 and 10 mg was −6% [23]. Based on this assumption, a sample size of approximately 99 patients per treatment group was calculated to provide 90% power to detect a difference of 6% (assuming a SD of 13%) and to detect the superiority of atorvastatin 20 mg over 10 mg with a two-sided alpha level of 0.05. Using a 1:1 sampling ratio and a dropout rate of 20%, a final sample size of 124 patients per treatment group (total 248 patients) was determined to provide an adequate evaluation.

## Results

### Baseline characteristics

**Fig 2:**
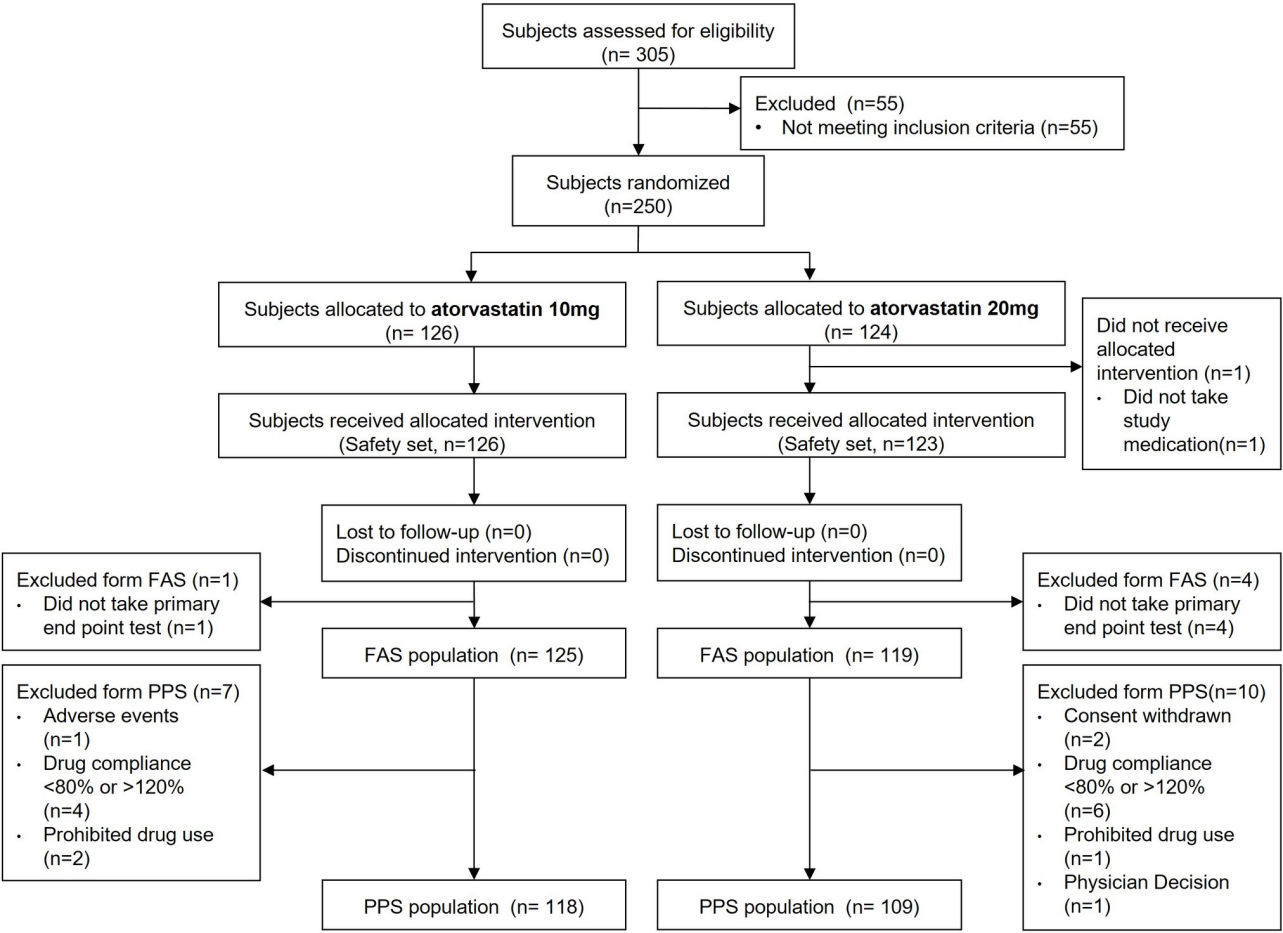
Consolidated Standards of Reporting Trials (CONSORT) flow diagram of patient disposition by analysis sets. FAS = full analysis set; PPS = per-protocol set.

Of the 305 patients who agreed to participate in this clinical trial, 55 patients failed to meet the inclusion criteria and were excluded. The remaining 250 patients were randomly assigned to receive either atorvastatin 10 mg or atorvastatin 20 mg. We included 249 patients in the safety set (one patient who had never taken the experimental drug) and 244 patients (except for five patients who did not undergo efficacy evaluation) in the FA set. During the trial, an additional 17 patients were excluded due to dropout, major protocol deviation, and drug non-compliance. Thus, 227 patients were included in the PP set (**Fig 2**). The demographic and baseline characteristics of the study participants, according to the group, are presented in **Table 1**. The characteristics of the participants in the two intervention groups were well balanced. The baseline lipid profiles and risk group stratification according to the 2015 Korean guidelines for dyslipidemia management were similar between both groups.

**Table 1:** Demographic and baseline characteristics of the study patients (FA set).

Variable	Atorvastatin 10mg (n = 125)	Atorvastatin 20mg (n = 119)	p-value
**Demographic**			
Age, mean (SD), y	62.8 (9.3)	62.8 (10.3)	0.9841^a^
Male (%)	96 (76.8%)	102 (85.7%)	0.0752^b^
BMI, mean (SD), kg/m^2^	25.1 (3.6)	25.1 (3.1)	0.9166^a^
**Risk group**			
Very high risk group	104 (83.2%)	103 (86.6%)	0.4652^b^
High risk group	21 (16.8%)	16 (13.5%)	
**Blood glucose parameters**			
Diabetes mellitus (%)	38 (30.4%)	34 (28.6%)	0.7542^c^
HbA1c, mean (SD) %	6.0 (0.7)	6.1 (0.9)	0.2186^a^
Fasting glucose, mean(SD), mg/dL	106.9 (24.5)	107.3 (26.5)	0.8968^a^
**Lipid profile**			
Total cholesterol, mean(SD), mg/dL	203.5 (35.7)	202.2 (39.0)	0.7880^a^
Triglycerides, mean (SD), mg/dL	186.9 (90.7)	166.9 (76.6)	0.0637^a^
HDL-C, mean (SD), mg/dL	43.2 (12.2)	44.6 (11.4)	0.3503^a^
LDL-C, mean (SD), mg/dL	142.6 (33.7)	142.1 (34.7)	0.9076^a^
Non-HDL-C, mean (SD), mg/dL	160.3 (34.3)	157.6 (36.4)	0.5501^a^
Apolipoprotein B, mean (SD), mg/dL	120.2 (24.0)	119.6 (25.8)	0.8439^a^
Apolipoprotein A1, mean (SD), mg/dL	130.2 (27.0)	130.6 (22.3)	0.8988^a^
**No. of patient achieving LDL-C goal**	-	-	n/a
**No. of patient achieving Non-HDL-C goal**	8(6.4%)	9(7.6%)	0.7213^c^

^a^ p-value of Independent t-test for comparison between groups

^b^ p-value of Fisher’s exact test for comparison between groups

^c^ p-value of Chi-square test for comparison between groups

### Efficacy

**Fig 3:**
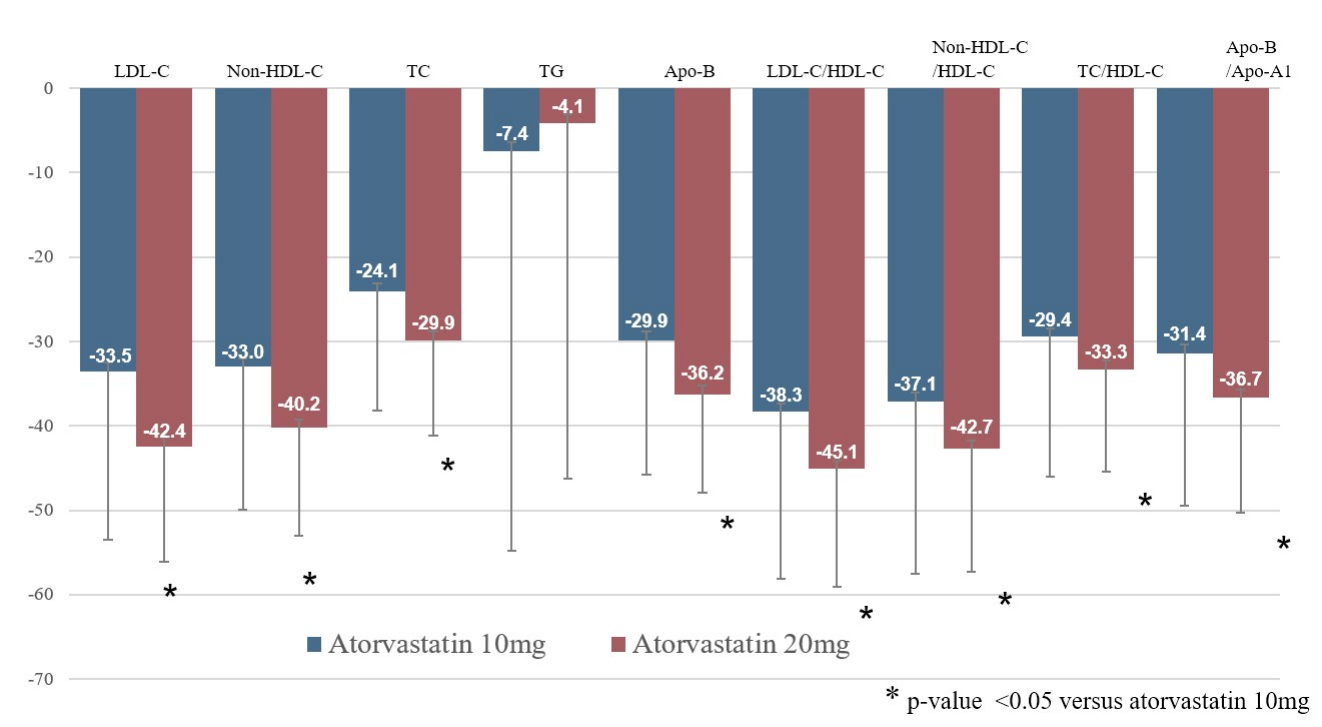
Changes from baseline in lipid parameters after treatment. LDL-C = low density lipoprotein cholesterol; HDL-C = high density lipoprotein cholesterol; TC = total cholesterol; TG = triglyceride; Apo-B = apolipoprotein B; Apo-A1 = apolipoprotein A1.

**Table 2** and **Fig 3** present the lipid profile changes after 12 weeks of treatment. In the FA set analysis, the LDL-C level decreased from 142.1 to 80.3 mg/dl (−42.4%, p < 0.0001) in the atorvastatin 20 mg group and from 142.6 to 92.3 mg/dl (−33.5%, p < 0.0001) in the atorvastatin 10 mg group after the 12-week treatment period. In the intergroup comparison, atorvastatin 20 mg resulted in a statistically significant reduction of LDL-C levels after 12 weeks of treatment from the baseline level compared to the atorvastatin 10 mg (group difference: 8.85%, p < 0.0001). Moreover, there were significant improvements in the levels of non-HDL-C, TC, HDL-C, and Apo-B in both groups after the 12-week treatment period. The percentage changes in non-HDL-C, TC, and Apo B levels were significantly greater in the atorvastatin 20 mg group than in the atorvastatin 10 mg group. The changes in HDL-C and ApoA1 levels showed no significant differences between the two groups. Comparative analysis of lipid ratios revealed significant improvements in LDL-C/HDL-C ratio, non-HDL-C/HDL ratio, TC/HDL-C ratio, and Apo-B/Apo-A1 ratio in both groups after the 12-week treatment period, with a significantly greater improvement in these lipid ratios in the atorvastatin 20 mg group. The PP set analysis results were similar to those of the FA set analysis (**S4 Table**).

**Table 2:** Percent change from baseline in lipid parameters after treatment (FA set).

Variable	Visit	Atorvastatin 10mg (n = 125)		Atorvastatin 20mg (n = 119)		Group difference
		Mean (SD)	p-value ^a^	Mean (SD)	p-value ^a^	p-value ^b^
LDL-C	Baseline	142.6 (33.7)		142.1 (34.7)		<0.0001
(mg/dL)	12 Week	92.3 (25.7)		80.3 (21.4)		
	% Change	-33.5	<0.0001	-42.4	<0.0001	
Non-HDL-C	Baseline	160.3 (34.3)		157.6 (36.4)		0.0002
(mg/dL)	12 Week	105.3 (25.8)		92.6 (22.5)		
	% Change	-33.0	<0.0001	-40.2	<0.0001	
TC	Baseline	203.5 (35.7)		202.2 (39.0)		0.0005
(mg/dL)	12 Week	152.1 (26.0)		139.7 (24.3)		
	% Change	-24.1	<0.0001	-29.9	<0.0001	
TG	Baseline	186.9 (90.7)		166.9 (76.6)		0.5687
(mg/dL)	12 Week	154.4 (82.0)		147.0 (76.5)		
	% Change	-7.4	0.0829	-4.1	0.2874	
HDL-C	Baseline	43.2 (12.2)		44.6 (11.4)		0.1502
(mg/dL)	12 Week	46.8 (12.2)		47.1 (12.3)		
	% Change	10.8	<0.0001	7.0	<0.0001	
Apo-A1	Baseline	130.2 (27.0)		130.6 (22.3)		0.2542
(mg/dL)	12 Week	133.6 (24.4)		132.0 (23.0)		
	% Change	4.0	0.0024	2.0	0.1012	
Apo-B	Baseline	120.2 (24.0)		119.6 (25.8)		0.0004
(mg/dL)	12 Week	83.2 (19.7)		75.1 (16.6)		
	% Change	-29.9	<0.0001	-36.2	<0.001	
LDL-C	Baseline	3.5 (1.1)		3.3 (0.9)		0.0022
/ HDL-C	12 Week	2.1 (0.8)		1.8 (0.6)		
ratio (%)	% Change	-38.3	<0.0001	-45.1	<0.0001	
Non-HDL-C	Baseline	4.0 (1.3)		3.7 (1.2)		0.0145
/ HDL-C	12 Week	2.4 (1.0)		2.1 (0.7)		
ratio (%)	% Change	-37.1	<0.0001	-42.7	<0.0001	
TC	Baseline	5.0 (1.3)		4.7 (1.2)		0.0390
/ HDL-C	12 Week	3.4 (1.0)		3.1 (0.7)		
ratio (%)	% Change	-29.4	<0.0001	-33.3	<0.0001	
Apo-B	Baseline	1.0 (0.3)		0.9 (0.2)		0.0101
/Apo-A1	12 Week	0.7 (0.2)		0.6 (0.2)		
ratio (%)	% Change	-31.4	<0.0001	-36.7	<0.0001	

%Change: {(12 Week-Baseline)/ Baseline}*100

^a^ p-value of paired t-test for the changes from baseline.

^b^ p-value of Independent t-test for comparison between groups

In the very high-risk group (n = 207), significantly more patients achieved the target LDL-C level (<70 mg/dL) at the 12^th^ week in the atorvastatin 20 mg group (35.0%, 36 of 103 patients) than in the atorvastatin 10 mg group (17.3%, 18 of 104 patients, p = 0.0038, **Table 3**). Also, significantly more very high-risk patients achieved the target non-HDL-C level (<100 mg/dL) at the 12^th^ week in the atorvastatin 20 mg group than in the atorvastatin 10 mg group (44.2%, 46 of 104 patients in the atorvastatin 10 mg group versus 59.2%, 61 of 103 patients in the atorvastatin 20 mg group, p = 0.0309, **Table 3**). In the analysis of the high-risk group patients (n = 37), more patients in the atorvastatin 20 mg group achieved the target LDL-C level (<100 mg/dL) at the 12^th^ week. However, it was not statistically significant (66.7%, 14 of 21 patients in atorvastatin 10 mg group versus 75.0%, 12 of 16 patients in the atorvastatin 20 mg group, p = 0.7228). There was no significant difference between the two groups in achieving the target non-HDL-C level (<130mg/dL) in the high-risk group patients (90.5%, 19 of 21 patients in the atorvastatin 10 mg group versus 87.5%, 14 of 16 patients in the atorvastatin 20 mg group, p = 1.0000). The achievement rate of target LDL-C level, irrespective of the risk group among all patients, was also significantly higher (p = 0.0142) in the atorvastatin 20 mg group (40.3%, 48 of 119 patients) than in the atorvastatin 10 mg group (25.6%, 32 of 125 patients). The achievement rate of target non-HDL-C level, irrespective of the risk group among all patients, was also higher in the atorvastatin 20 mg group (63.0%, 75 of 119 patients) than in the atorvastatin 10 mg (52.0%, 65 of 125 patients). Still, the difference was not statistically significant (p = 0.0817). The achievement rate of target LDL-C and non-HDL-C levels in the PP set were similar to those of the FA set analysis (**S5 Table**).

**Table 3:** Rate of achievement of LDL-C and non-HDL-C target at 12^th^ week (FA set).

Variable	Atorvastatin 10mg		Atorvastatin 20mg		p-value
	n	(%)	n	(%)	
**Very high risk group**^**a**^	n = 104		n = 103		
**LDL-C <70mg/dL**	18	17.3%	36	35.0%	0.0038^c^
**LDL-C ≥ 70mg/dL**	86	82.7%	67	65.1%	
**Non-HDL-C <100mg/dL**	46	44.2%	61	59.2%	0.0309^c^
**Non-HDL-C ≥100mg/dL**	58	55.8%	42	40.8%	
**High risk group**^**b**^	n = 21		n = 16		
**LDL-C <100mg/dL**	14	66.7%	12	75.0%	0.7228^d^
**LDL-C ≥ 100mg/dL**	7	33.3%	4	25.0%	
**Non-HDL-C <130mg/dL**	19	90.5%	14	87.5%	1.0000^d^
**Non-HDL-C ≥130mg/dL**	2	9.5%	2	12.5%	
**All patient**	n = 125		n = 119		
**Target LDL-C achieved**	32	25.6%	48	40.3%	0.0142^c^
**Target LDL-C not achieved**	93	74.4%	71	59.7%	
**Target Non-HDL-C achieved**	65	52.0%	75	63.0%	0.0817^c^
**Target Non-HDL-C not achieved**	60	48.0%	44	37.0%	

^a^ Patients with coronary artery disease, ischemic Stroke, transient ischemia attack, peripheral arterial disease

^b^ Patients with carotid artery disease, abdominal aneurysm, diabetes

^c^ p-value of Chi-square test for comparison between groups

^d^ p-value of Fisher’s exact test for comparison between groups

### Safety

**Fig 4:**
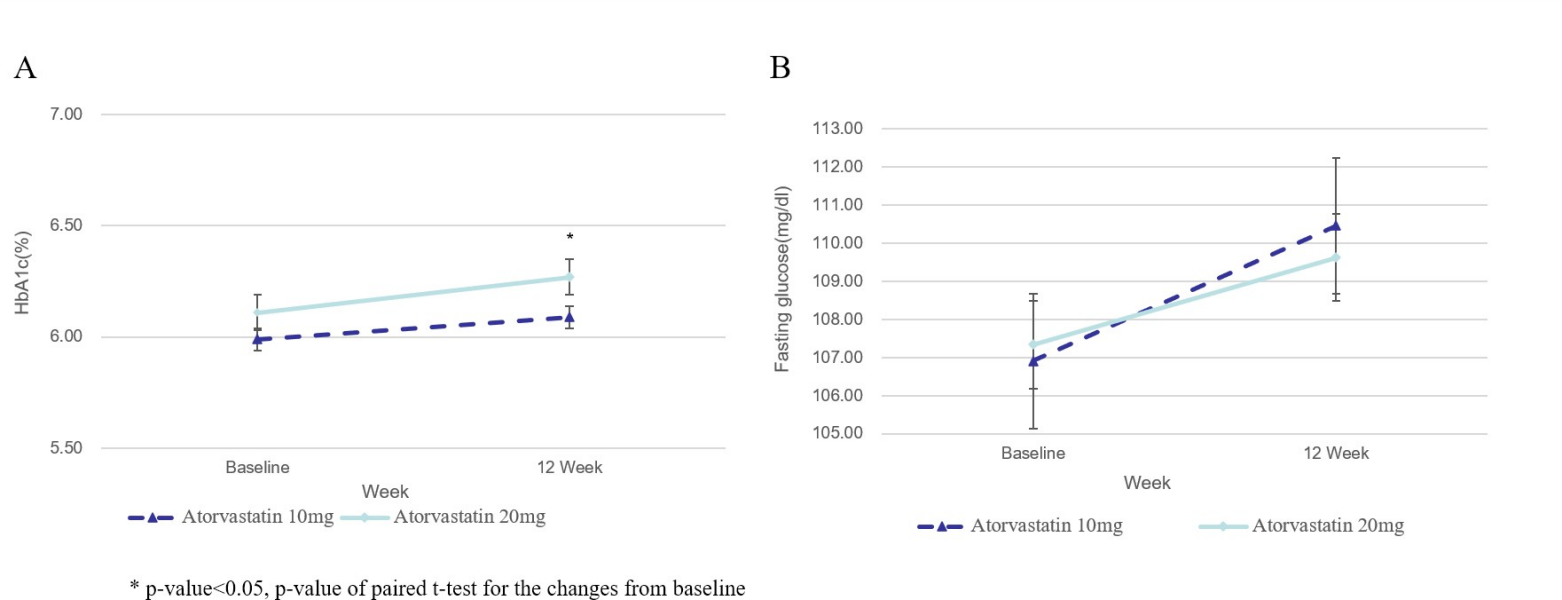
Changes from baseline in blood glucose after treatment. (A) Change of HbA1c after treatment. (B) Change of fasting glucose after treatment.

After 12 weeks of atorvastatin treatment, the HbA1c level was slightly, but statistically significantly, increased in both treatment groups (**Fig 4**, **Table 4**). In the atorvastatin 20 mg group, the HbA1c level increased from a baseline value of 6.1% to 6.3% after 12 weeks (p = 0.0149). Similarly, in the atorvastatin 10 mg group, the HbA1c level was increased from a baseline value of 6.0% to 6.1% after 12 weeks (p = 0.0057). No intergroup differences were detected in the 12^th^ week between the two groups (p = 0.4525). There were also increments in fasting glucose levels in both groups at the 12^th^ week, although not statistically significant, and there were no intergroup differences. The results of the PP set analysis for HbA1c and fasting glucose levels were similar to those of the FA set analysis (**S6 Table**). In patients with low risk of diabetes defined as a body mass index of <30 kg/m^2^, a fasting glucose level of <100 mg/dL, and an HbA1c level of <6%, both the atorvastatin 20 and 10 mg groups showed small but significant increases in HbA1c levels. There was no difference between the two groups (**S7 Table**). The levels of creatine kinase were increased slightly but, significantly after 12 weeks of atorvastatin treatment compared to baseline in both the atorvastatin 10mg and 20mg group without exceeding the normal range in both groups. And there was no difference between the two groups in levels of creatine kinase (**S8 Table**).

**Table 4:** Changes from baseline in HbA1c and fasting blood glucose after treatment (FA set).

Variable	Visit	Atorvastatin 10mg		Atorvastatin 20mg		Group difference
		(n = 125)		(n = 119)		
		Mean (SD)	p-value ^a^	Mean (SD)	p-value ^a^	p-value ^b^
HbA1c (%)	Baseline	6.0 (0.7)		6.1 (0.9)		0.4525
	12 Week	6.1 (0.8)		6.3 (1.1)		
	Change	0.1	0.0057	0.2	0.0149	
Fasting glucose	Baseline	106.9 (24.5)		107.3 (26.5)		0.7013
(mg/dL)	12 Week	110.5 (34.1)		109.6(32.7)		
	Change	3.5	0.0998	2.3	0.3544	

Change: 12 Week-Baseline

^a^ p-value of paired t-test for the changes from baseline.

^b^ p-value of Independent t-test for comparison between groups

During this clinical trial, eight ADRs in seven patients had a causal relationship with the study drug in the safety set (2.8%, 7 of 249 patients). Two ADRs were reported in two patients in the atorvastatin 10 mg group (1.6%, 2 of 126 patients), and six ADRs were reported in five patients in the atorvastatin 20 mg group (4.1%, 5 of 123 patients). The most frequent ADRs were myalgia (two patients in the atorvastatin 20 mg group) and hepatic enzyme elevation (two patients in the atorvastatin 20 mg group). There was no statistically significant difference in the incidence of ADRs between the two groups (**Table 5**).

**Table 5:** Comparison of adverse drug reactions between atorvastatin 10mg and 20mg (safety set analysis).

Preferred Term	Total	Atorvastatin 10mg	Atorvastatin 20mg	p-value
	(n = 249)	(n = 126)	(n = 123)	
Myalgia	2 (0.8%)	-	2 (1.6%)	0.2430^a^
Hepatic enzyme elevation	2 (0.8%)	-	2 (1.6%)	0.2430 ^a^
Fatigue	1 (0.4%)	1 (0. 8%)	-	1.0000 ^a^
Dizziness	1 (0.4%)	1 (0.8%)	-	1.0000 ^a^
Chromaturia	1 (0.4%)	-	1 (0.8%)	0.4940 ^a^
Pruritus	1 (0.4%)	-	1 (0.8%)	0.4940 ^a^

^a^ p-value of Fisher’s exact test for comparison between groups

### Cost-effectiveness analysis

ACER and ICER were used to analyze the cost-effectiveness of atorvastatin 10 and 20 mg. The ACER value was significantly lower in the atorvastatin 20 mg group than in the atorvastatin 10 mg group. The costs required to reduce 1% of LDL-C levels were Korean Won (₩) 2604 in the atorvastatin 10 mg group and ₩2074 in the atorvastatin 20 mg group (**Table 6**). The ICER value analysis showed that atorvastatin 20 mg had an 8.9% greater LDL-C reduction effect than atorvastatin 10 mg, and the total cost was reduced to as much as ₩3480. Consequently, the cost required to reduce 1% of LDL-C levels was lower at ₩393, with atorvastatin 20 mg than with atorvastatin 10 mg (**Table 6**).

**Table 6:** Comparison of cost-effectiveness of atorvastatin 10mg and 20mg.

Variable	Atorvastatin 10mg (n = 125)			Atorvastatin 20mg (n = 119)			Group difference		ICER ^b^
	Cost	LDL reduction	ACER^a^	Cost	LDL reduction	ACER*	Δ Cost	ΔLDL reduction	
	(₩)	(%)	(₩/%)	(₩)	(%)	(₩/%)		(%)	
**Mean (SD)**	56,834.5	-33.5	2,604.0	52,357.9	-42.4	2074.0	-3,480.7	-8.85	393.3
	(7,516.8)			(8,545.0)					
**Median**	58,302.0	-38.4	1,499.5	54,202.0	-44.9	1218.0			
**Min**	8,532.0	-62.3	275.7	9,915.0	-72.0	188.9			
**Max**	71,100.0	77.4	65,923.9	66,100.0	22.4	76,742.1			

^a^ ACER; average cost-effectiveness ratio

^b^ ICER; incremental cost-effectiveness ratio

## Discussion

High-quality evidence, from several randomized controlled trials, supports the benefits of statin therapy for the primary and secondary prevention of CVD. Although there are some differences in the criteria for initiating statin therapy and in the treatment goals according to the guideline used, the target of LDL-C is getting lower and more potent statins are recommended. However, the response to a statin in a particular individual is so unpredictable that it is not easy for physicians to choose the optimal statin for a specific patient. Thus, physicians need to refine the statin therapy beyond the general guideline recommendations on a case-by-case basis in their practice. However, in some cases, several factors such as unawareness of guideline recommendations, concern over adverse reactions, and therapeutic inertia can lead physicians to prescribe a suboptimal dose of statin [24, 25]. In particular, in the Asian population, the use of suboptimal dose statins and low achievement rates of target LDL-C levels are widespread issues. Both previous and recent studies report that a substantial proportion of individuals fail to achieve the recommended LDL-C target even among high-risk patients. In the REALITY-Asia study, conducted in 2622 patients from China, Korea, Malaysia, Singapore, Taiwan, and Thailand in 2008, only 38% of high-risk patients achieved ATP III target levels for LDL-C (<100 mg/dL) [26]. In a recent study published in 2020, 69,942 Korean patients with dyslipidemia were stratified according to the risk, based on the 2015 Korean guidelines as done in our research, and the achievement rate of target LDL-C levels was analyzed. In that study, similar to the previous research published 12 years ago, the achievement rate of target LDL-C levels was very suboptimal as only 17.6% of very high-risk patients and 47.2% of high-risk patients achieved the target [27]. Also, investigations conducted in other Asian countries reported low LDL-C target achievement rates [17, 19, 28]. This phenomenon is a bit disappointing since the Asian population has a lower baseline level of LDL-C and greater statin responsiveness than the Western population [29–31]. However, the importance of optimal LDL-C goal attainment is no matter of debate to prevent future CVD. Attainment of the suboptimal LDL-C target is related to the increased risk of developing future CVD, and the benefit of statin treatment is more evident in high-risk patients [20, 32, 33]. Therefore, achieving the target LDL-C level is essential irrespective of ethnicity, and the most critical determinants for this achievement are lipid-lowering treatment with statin and adherence to the lipid-lowering treatment [24, 28]. But unconditional usage of high-dose statin cannot always be the best choice because even if the dose of statin is doubled, the additional LDL-C lowering effect is only 5%–7%. Furthermore, the risk of adverse reactions [34] and the costs increase along with the statin dose.

In the present study, treatments with atorvastatin 10 and 20 mg over a 12-week period were compared to explore the dose of atorvastatin that is more appropriate in high-risk Asian patients in terms of efficacy, safety, and cost-effectiveness. As reported by earlier studies regarding the effectiveness and safety of atorvastatin in Asian patients even at a high dose [35, 36], the present study also demonstrated that atorvastatin effectively improved various lipid profiles without increasing the rate of ADRs. The percentage change in LDL-C levels after atorvastatin therapy was comparable to that reported by previous studies [35, 37, 38]. The reduction of LDL-C, non-HDL-C, TC, and Apo-B levels was significantly higher in the atorvastatin 20 mg group than in the atorvastatin 10 mg group. Regarding the levels of TG, HDL-C, and Apo A-I, both groups displayed a tendency to improve after 12 weeks compared to the baseline levels. Still, there were no statistically significant differences between the groups. These relatively small and nonsignificant changes in TG, HDL-C, and Apo-A1 levels were also similar to those of previous studies [35, 37, 38]. Atorvastatin 20 was more efficient than atorvastatin 10 mg in for the very high-risk group patients in achieving target LDL-C and non-HDL-C levels. During the clinical trial, there were only a few ADRs, with no difference in the incidence of ADRs between the two groups. Overall, atorvastatin 20 mg exhibited a dose-dependent effect in improving lipid profiles without increasing the incidence of ADRs compared with atorvastatin 10 mg in the high-risk Asian patients.

Furthermore, atorvastatin 20 mg was more cost-effective than atorvastatin 10 mg in our study. Atorvastatin 20 mg has a low ACER value, implying that it costs less to reduce a certain amount of LDL-C when using atorvastatin 20 mg than using atorvastatin 10 mg. However, ACER alone may not be the best indicator for choosing a more cost-effective medicine in real practice because a low ACER value can arise in both cases when a medication has a small effect at low cost and a high effect at a high cost. Therefore, it is important to consider the size of the impact along with the cost. Hence, the ICER is another good indicator for choosing a more cost-effective medicine between two medications as it can compare the cost according to the size of the effect. In our study’s ICER analysis, atorvastatin 20 mg demonstrated a more significant LDL-C-reducing effect at a lower cost, and the cost required to reduce 1% of LDL-C levels was found to be lower with atorvastatin 20 mg than with atorvastatin 10 mg. These results are remarkable, considering that the dose-effect relationship of statin is not linear, and that the LDL-C reduction effect is prominent at lower doses. In other words, although the administration of more moderate-dose statins is relatively advantageous in terms of cost, this could not offset the higher LDL-C reduction effect due to a higher dose of statin administration. Furthermore, using a lower dose of statin can increase the total cost because of repetitive monitoring and visiting that would be necessary due to the low achievement rate of target LDL-C levels. Therefore, avoiding the use of lower dose atorvastatin can be a cost-effective alternative to prevent CVD in a longer-term perspective.

In the present study, the HbA1c level increased significantly after 12 weeks of atorvastatin treatment. However, the mean elevation of the HbA1c level was not prominent, which was only around 0.1% irrespective of the atorvastatin dose. The elevated HbA1c level was commonly found in all patients as well as in nondiabetic patients with low risk of diabetes (BMI <30 kg/m^2^, fasting glucose level <100 mg/dL, and HbA1c level <6%). Although statins increase blood glucose levels and the risk of diabetes mellitus in a dose-dependent manner [39], there was no significant difference between the atorvastatin 10 and 20 mg groups. Also, our results revealed the elevation of HbA1c levels after a relatively short-term statin treatment of 12 weeks. As shown in our study, an increase in blood glucose level induced by a statin is not just a chronic, long-term change. Other studies on statin conducted for a relatively short period also demonstrated elevated blood glucose levels, and statin treatment for only a few days in the early phase of acute myocardial infarction also reduced insulin sensitivity [35, 40]. Therefore, although the benefit of LDL-C reduction is known to outweigh the harm from the elevation of blood glucose [41], it is necessary to monitor blood glucose levels from the early days of the statin treatment even in patients with low risk of diabetes.

### Limitations

The present study has some limitations. First, high-dose statins recommended for high-risk patients in the latest guidelines were not used in this study. Although the atorvastatin dose used in this study may be closer to the dose frequently used in real-world practice, further investigation using higher doses of atorvastatin would be necessary. Second, as the proportion of patients with CAD was too high, the composition of the study patients was relatively homogenous, and most of the patients were considered very high risk. Consequently, the number of high-risk patients was too small for statistical analysis. Third, statins can affect the inflammatory process in atherosclerosis, which is one of the important effects of statin, but inflammatory marker such as high sensitivity C-reactive protein was not measured in this study. Fourth, the cost-effectiveness results cannot be generalized to other countries as it was calculated under the Korean healthcare system. Fifth, the incidence of AEs caused by statin treatment was lower than that in previous studies. This may be due to the small sample size neglecting the mild symptoms.

## Conclusion

Atorvastatin 20 mg was more effective in reducing LDL-C levels in Asian patients with high risk for CVD than atorvastatin 10 mg and also resulted in statistically significant improvement in most of the lipid profiles. Atorvastatin 20 mg did not increase the incidence of ADRs and was more cost-effective than atorvastatin 10 mg. Therefore, atorvastatin 20 mg can be a more appropriate dose of choice than atorvastatin 10 mg in Asian patients with a high risk for CVD.

## Supporting information

S1 Table Further exclusion criteria. (DOCX)

S2 Table List of centers and local principal investigators. (DOCX)

S3 Table Baseline assessments. (DOCX)

S4 Table Changes from baseline in lipid parameters after treatment (PP set). (DOCX)

S5 Table Rate of achievement of LDL-C and non-HDL-C target at 12^th^ week (PP set). (DOCX)

S6 Table Changes from baseline in HbA1c and fasting blood glucose after treatment (PP set). (DOCX)

S7 Table Changes from baseline in HbA1c and fasting blood glucose after treatment in non-diabetic patient with low risk of diabetes (BMI <30kg/m^2^ & fasting glucose <100mg/dL & HbA1c <6%, FA set). (DOCX)

S8 Table Changes from baseline in creatine kinase levels after treatment (safety set). (DOCX)

S1 File Clinical study protocol (English version). (PDF)

S2 File Clinical study protocol (Korean version). (PDF)
