# Correction: A randomized, open-label, parallel, multi-center Phase IV study to compare the efficacy and safety of atorvastatin 10 and 20 mg in high-risk Asian patients with hypercholesterolemia

**DOI:** 10.1371/journal.pone.0259072

**Published:** 2021-10-20

**Authors:** Ji Bak Kim, Woo Hyuk Song, Jong Sung Park, Tae-Jin Youn, Yong Hyun Park, Shin-Jae Kim, Sung Gyun Ahn, Joon-Hyung Doh, Yun-Hyeong Cho, Jin Won Kim

The following information is missing from the Funding statement: This work was supported by the National Research Foundation of Korea (NRF-2018R1A2B3002001 to J.W.K and NRF-2019M3A9E2066882 to J.W.K).
