# Supplementary material for: A randomized, open-label, parallel, multi-center Phase IV study to compare the efficacy and safety of atorvastatin 10 and 20 mg in high-risk Asian patients with hypercholesterolemia
Source: PLoS One. 2021 Jan 22;16(1):e0245481. doi: 10.1371/journal.pone.0245481 (PMC7822387; doi:10.1371/journal.pone.0245481)
Supplement: S1 Table — (DOCX) [file pone.0245481.s001.docx]

**S1 Table**. **Further exclusion criteria**

| ▶ Patients who are in a medical or surgical condition that can affect the absorption, distribution, metabolism, and excretion of investigational products  -Patients with a history of major gastrointestinal surgery such as gastrectomy, gastrointestinal bypass surgery or anastomosis (except simple blind or hernia surgery)  -Patients with a history of active inflammatory bowel syndrome within the last 12 months  -Patients who need treatment due to Crohn’s disease, pancreatic dysfunction such as pancreatitis, biliary stasis, etc. or gastrointestinal/rectal bleeding.  ▶ Those who have a history of hypersensitivity or allergies to the study drug.  ▶ Patients with a history of drug or alcohol abuse within the last 6 months  ▶ Patients who do not agree to contraception (however, for women who have been amenorrhea for at least 12 months or more, it is considered as menopause)  ▶ Patients who have taken another clinical trial drug within the past 30 days  ▶ Patients who are difficult to discontinue taking other medications that affect lipid levels during the clinical trial  ▶ Patients who need to take contraindicated medication specified in the protocol.  ▶ Patients with genetic problems such as galactose intolerance, Lapp lactase deficiency, or glucose-galactose malabsorption  ▶ Patients who are legally unable to participate in clinical trials or who are unable to participate in clinical trials based on investigator’s decision |
| --- |
