# Supplementary material for: A randomized, open-label, parallel, multi-center Phase IV study to compare the efficacy and safety of atorvastatin 10 and 20 mg in high-risk Asian patients with hypercholesterolemia
Source: PLoS One. 2021 Jan 22;16(1):e0245481. doi: 10.1371/journal.pone.0245481 (PMC7822387; doi:10.1371/journal.pone.0245481)
Supplement: S2 Table — (DOCX) [file pone.0245481.s002.docx]

**S2 Table**. **List of centers and local principal investigators**

| No. | Principal investigator | Affiliation |
| --- | --- | --- |
| 1 | Jin Won Kim | Cardiovascular Center, Korea University Guro Hospital, Seoul, Korea |
| 2 | Woo Hyuk Song | Division of Cardiology, Department of Internal Medicine, Korea University Ansan Hospital, Ansan, Korea. |
| 3 | Jong Sung Park | Department of Cardiology, Dong-A University Hospital, Busan, Korea |
| 4 | Tae-Jin Youn | Division of Cardiology, Department of Internal Medicine, College of Medicine, Seoul National University and Cardiovascular Center, Seoul National University Bundang Hospital, Seongnam, Korea |
| 5 | Ji Bak Kim | Division of Cardiology, Sejong General Hospital, Bucheon, Korea. |
| 6 | Yong Hyun Park | Cardiovascular Center, Division of Cardiology, Department of Internal Medicine, Pusan National University Yangsan Hospital, Yangsan, Korea |
| 7 | Shin-Jae Kim | Department of Cardiology, Ulsan University Hospital, University of Ulsan College of Medicine, Ulsan, Korea |
| 8 | Sung Gyun Ahn | Division of Cardiology, Department of Internal Medicine, Wonju Severance Christian Hospital, Wonju, Korea |
| 9 | Joon-Hyung Doh | Department of Cardiology, Inje University Ilsan Paik Hospital, Goyang, Korea |
| 10 | Yun-Hyeong Cho | Department of Internal Medicine, Myongji Hospital, Goyang, Korea. |
