# Supplementary material for: A randomized, open-label, parallel, multi-center Phase IV study to compare the efficacy and safety of atorvastatin 10 and 20 mg in high-risk Asian patients with hypercholesterolemia
Source: PLoS One. 2021 Jan 22;16(1):e0245481. doi: 10.1371/journal.pone.0245481 (PMC7822387; doi:10.1371/journal.pone.0245481)
Supplement: S3 Table — (DOCX) [file pone.0245481.s003.docx]

**S3 Table. Baseline assessments**

| Blood tests | RBC, Hb, Hct,  WBC with differential count  (Neutrophil, Lymphocyte, Monocyte,  Eosinophil, Basophil)  Platelet  Ca, P, Uric acid  Glucose, HbA1c  BUN, Creatinine  Total Protein, Albumin,  Total Bilirubin, Direct Bilirubin, AST, ALT, ALP,  LDH, γ-GT, CPK,  Na, K, Cl  TSH, Free T4  LDL-C, HDL-C, TG, Total cholesterol,  Apo-A1, Apo-B, Non-HDL-C/HDL-C ratio,  Total Cholesterol/HDL-C ratio, LDL-C/HDL-C ratio, Apo-B/Apo-A1 |
| --- | --- |
| Urine analysis | Specific Gravity, PH, Protein(Albumin), Glucose, Ketone, Occult Blood, Urobilinogen, Nitrite |

Laboratory tests were performed on fasting state (at least 9 hours of fasting) to rule out dietary effects.
