# Supplementary material for: A randomized, open-label, parallel, multi-center Phase IV study to compare the efficacy and safety of atorvastatin 10 and 20 mg in high-risk Asian patients with hypercholesterolemia
Source: PLoS One. 2021 Jan 22;16(1):e0245481. doi: 10.1371/journal.pone.0245481 (PMC7822387; doi:10.1371/journal.pone.0245481)
Supplement: S4 Table — (DOCX) [file pone.0245481.s004.docx]

**S4 Table. Changes from baseline in lipid parameters after treatment (PP set)**

| **Variable** | **Visit** | **Atorvastatin 10mg (n= 118)** | | | | | **Atorvastatin 20mg (n=109)** | | | | | **Group difference** |
| --- | --- | --- | --- | --- | --- | --- | --- | --- | --- | --- | --- | --- |
|  |  | Mean (SD) | Median | Min | Max | P-value* | Mean (SD) | Median | Min | Max | P-value* | P-value** |
| **LDL-C (mg/dL)** | Baseline | 144.2 (33.5) | 143.5 | 74.0 | 239.0 |  | 141.6 (34.8) | 140.0 | 76.0 | 248.0 |  | 0.0005 |
|  | 12 Week | 91.9 (24.6) | 90.5 | 43.0 | 188.0 |  | 80.5 (21.3) | 76.0 | 43.0 | 145.0 |  |  |
|  | % Change | -34.9 | -39.4 | -62.3 | 32.6 | <0.0001 | -42.0 | -44.0 | -72.0 | 22.4 | <0.0001 |  |
| **Non-HDL-C (mg/dL)** | Baseline | 162.3 (33.6) | 160.5 | 87.0 | 242.0 |  | 157.2 (36.8) | 154.0 | 94.0 | 277.0 |  | 0.0014 |
|  | 12 Week | 105.2 (25.4) | 101.0 | 51.0 | 188.0 |  | 92.7 (22.3) | 88.0 | 44.0 | 163.0 |  |  |
|  | % Change | -34.3 | -36.5 | -59.8 | 28.3 | <0.0001 | -40.0 | -40.9 | -66.5 | 22.3 | <0.0001 |  |
| **TC**  **(mg/dL)** | Baseline | 204.8 (35.5) | 199.5 | 132.0 | 283.0 |  | 201.5 (39.6) | 200.0 | 123.0 | 329.0 |  | 0.0058 |
|  | 12 Week | 151.5 (25.4) | 151.0 | 95.0 | 224.0 |  | 139.9 (24.3) | 142.0 | 82.0 | 210.0 |  |  |
|  | % Change | -25.1 | -27.2 | -47.7 | 31.8 | <0.0001 | -29.5 | -29.9 | -57.3 | 25.2 | <0.0001 |  |
| **TG**  **(mg/dL)** | Baseline | 186.9 (85.6) | 175.5 | 52.0 | 456.0 |  | 165.8 (77.0) | 156.0 | 62.0 | 412.0 |  | 0.7254 |
|  | 12 Week | 156.5 (82.8) | 134.0 | 57.0 | 504.0 |  | 145.0 (75.6) | 128.0 | 51.0 | 601.0 |  |  |
|  | % Change | -6.8 | -12.1 | -80.1 | 185.7 | 0.1277 | -4.7 | -12.9 | -75.6 | 136.5 | 0.2483 |  |
| **HDL-C**  **(mg/dL)** | Baseline | 42.6 (12.0) | 40.0 | 25.0 | 89.0 |  | 44.3 (11.5) | 43.0 | 23.0 | 71.0 |  | 0.2579 |
|  | 12 Week | 46.2 (11.9) | 45.0 | 20.0 | 90.0 |  | 47.2 (12.4) | 45.0 | 25.0 | 90.0 |  |  |
|  | % Change | 11.2 | 7.3 | -41.9 | 100.0 | <0.0001 | 8.1 | 5.6 | -26.8 | 79.4 | <0.0001 |  |
| **Apo-A1 (mg/dL)** | Baseline | 129.0 (26.6) | 123.0 | 85.0 | 254.0 |  | 129.9 (22.7) | 127.0 | 81.0 | 202.0 |  | 0.4289 |
|  | 12 Week | 132.8 (23.9) | 131.0 | 73.0 | 219.0 |  | 132.5 (23.4) | 130.0 | 87.0 | 243.0 |  |  |
|  | % Change | 4.4 | 2.7 | -42.6 | 50.6 | 0.0016 | 2.9 | 1.8 | -34.6 | 36.2 | 0.0233 |  |
| **Apo-B**  **(mg/dL)** | Baseline | 121.5 (23.8) | 120.5 | 74.0 | 186.0 |  | 119.4 (26.0) | 116.0 | 73.0 | 199.0 |  | 0.0014 |
|  | 12 Week | 83.1 (19.2) | 82.0 | 45.0 | 144.0 |  | 74.9 (16.3) | 73.0 | 48.0 | 128.0 |  |  |
|  | % Change | -30.9 | -32.5 | -53.4 | 15.4 | <0.0001 | -36.2 | -36.7 | -64.0 | 7.2 | <0.0001 |  |
| **LDL-C / HDL-C ratio (%)** | Baseline | 3.6 (1.0) | 3.5 | 1.1 | 6.5 |  | 3.3(0.9) | 3.4 | 1.3 | 5.6 |  | 0.0076 |
|  | 12 Week | 2.1(0.8) | 2.0 | 0.7 | 5.7 |  | 1.8(0.6) | 1.7 | 0.6 | 3.5 |  |  |
|  | % Change | -39.8 | -43.2 | -72.1 | 26.7 | <0.0001 | -45.4 | -48.2 | -72.2 | 7.4 | <0.0001 |  |
| **Non-HDL-C / HDL-C ratio (%)** | Baseline | 4.1(1.2) | 4.1 | 1.1 | 7.2 |  | 3.8(1.2) | 3.7 | 1.3 | 6.7 |  | 0.0359 |
|  | 12 Week | 2.5(1.0) | 2.3 | 0.7 | 6.6 |  | 2.1(0.7) | 2.0 | 0.5 | 3.6 |  |  |
|  | % Change | -38.5 | -41.5 | -74.8 | 35.7 | <0.0001 | -43.1 | -43.6 | -69.8 | 3.3 | <0.0001 |  |
| **TC / HDL-C ratio (%)** | Baseline | 5.1(1.2) | 5.1 | 2.1 | 8.2 |  | 4.8(1.2) | 4.7 | 2.3 | 7.7 |  | 0.0943 |
|  | 12 Week | 3.5(1.0) | 3.3 | 1.7 | 7.6 |  | 3.1(0.7) | 3.0 | 1.5 | 4.6 |  |  |
|  | % Change | -30.6 | -33.5 | -65.1 | 29.4 | <0.0001 | -33.6 | -34.5 | -56.8 | 2.5 | <0.0001 |  |
| **Apo-B /Apo-A1 ratio (%)** | Baseline | 1.0(0.3) | 1.0 | 0.4 | 1.8 |  | 0.9(0.2) | 0.9 | 0.5 | 1.5 |  | 0.0192 |
|  | 12 Week | 0.7(0.2) | 0.6 | 0.3 | 1.6 |  | 0.6 (0.2) | 0.6 | 0.2 | 1.0 |  |  |
|  | % Change | -32.6 | -35.2 | -58.6 | 57.4 | <0.0001 | -37.2 | -38.7 | -60.5 | 27.8 | <0.0001 |  |

%Change: {(12 Week-Baseline)/ Baseline}*100

*: P-value of paired t-test for the changes from baseline.

**: P-value of Independent t-test for comparison between groups
