# Supplementary material for: A randomized, open-label, parallel, multi-center Phase IV study to compare the efficacy and safety of atorvastatin 10 and 20 mg in high-risk Asian patients with hypercholesterolemia
Source: PLoS One. 2021 Jan 22;16(1):e0245481. doi: 10.1371/journal.pone.0245481 (PMC7822387; doi:10.1371/journal.pone.0245481)
Supplement: S5 Table — (DOCX) [file pone.0245481.s005.docx]

**S5 Table.** **Rate of achievement of LDL-C and non-HDL-C target at 12^th^ week (PP set)**

| **Variable** | **Atorvastatin 10mg (n=118)** | | **Atorvastatin 20mg (n=109)** | | **P-value** |
| --- | --- | --- | --- | --- | --- |
|  | **n** | **(%)** | **n** | **(%)** |  |
|  |  | |  | |  |
| **Very high risk group*** | n=97 | | n=94 | |  |
| **LDL-C <70mg/dL** | 16 | 16.5 | 32 | 34.0 | 0.0052^†^ |
| **LDL-C ≥ 70mg/dL** | 81 | 83.5 | 62 | 66.0 |  |
|  |  | |  | |  |
| **Non-HDL-C <100mg/dL** | 41 | 42.3 | 56 | 59.6 | 0.0168^†^ |
| **Non-HDL-C ≥100mg/dL** | 56 | 57.7 | 38 | 40.4 |  |
|  |  | |  | |  |
| **High risk group**** | n=21 | | n=15 | |  |
| **LDL-C <100mg/dL** | 14 | 66.7 | 12 | 80.0 | 0.4682^‡^ |
| **LDL-C ≥ 100mg/dL** | 7 | 33.3 | 3 | 20.0 |  |
|  |  | |  | |  |
| **Non-HDL-C <130mg/dL** | 19 | 90.5 | 14 | 93.3 | 1.0000^‡^ |
| **Non-HDL-C ≥130mg/dL** | 2 | 9.5 | 1 | 6.7 |  |

*Patients with coronary artery disease, ischemic Stroke, transient ischemia attack, peripheral arterial disease

**Patients with carotid artery disease, abdominal aneurysm, diabetes

^†^ P-value of Chi-square test for comparison between groups

^‡^ P-value of Fisher’s exact test for comparison between groups
