# Supplementary material for: A randomized, open-label, parallel, multi-center Phase IV study to compare the efficacy and safety of atorvastatin 10 and 20 mg in high-risk Asian patients with hypercholesterolemia
Source: PLoS One. 2021 Jan 22;16(1):e0245481. doi: 10.1371/journal.pone.0245481 (PMC7822387; doi:10.1371/journal.pone.0245481)
Supplement: S6 Table — (DOCX) [file pone.0245481.s006.docx]

**S6 Table.** **Changes from baseline in HbA1c and fasting blood glucose after treatment (PP set)**

| **Variable** | **Visit** | **Atorvastatin 10mg (n= 118)** | | | | | **Atorvastatin 20mg (n=109)** | | | | | **Group difference** |
| --- | --- | --- | --- | --- | --- | --- | --- | --- | --- | --- | --- | --- |
|  |  | **Mean (SD)** | **Median** | **Min** | **Max** | **P-value*** | **Mean (SD)** | **Median** | **Min** | **Max** | **P-value*** | **P-value**** |
|  |  |  |  |  |  |  |  |  |  |  |  |  |
| **HbA1c (%)** | Baseline | 6.0(0.7) | 5.9 | 4.7 | 8.8 |  | 6.1(0.8) | 5.9 | 4.8 | 8.9 |  | 0.8011 |
|  | 12 Week | 6.1(0.8) | 5.9 | 4.9 | 9.3 |  | 6.2(0.9) | 6.0 | 5.0 | 10.1 |  |  |
|  | Change | 0.1 | 0.05 | -1.4 | 1.5 | 0.0076 | 0.12 | 0.1 | -2.4 | 2.1 | 0.0210 |  |
|  |  |  |  |  |  |  |  |  |  |  |  |  |
| **Fasting  glucose  (mg/dL)** | Baseline | 106.9 (24.8) | 100.0 | 68.0 | 249.0 |  | 107.1 (24.6) | 102.0 | 74.0 | 225.0 |  | 0.4327 |
|  | 12 Week | 111.0 (34.9) | 99.0 | 75.0 | 356.0 |  | 108.8 (31.2) | 103.0 | 73.0 | 257.0 |  |  |
|  | Change | 4.1 | 2.0 | -32.0 | 209.0 | 0.0665 | 1.7 | -1.0 | -58.0 | 129.0 | 0.4285 |  |

Change: 12 Week-Baseline

*: P-value of paired t-test for the changes from baseline.

**: P-value of Independent t-test for comparison between groups
