# Supplementary material for: A randomized, open-label, parallel, multi-center Phase IV study to compare the efficacy and safety of atorvastatin 10 and 20 mg in high-risk Asian patients with hypercholesterolemia
Source: PLoS One. 2021 Jan 22;16(1):e0245481. doi: 10.1371/journal.pone.0245481 (PMC7822387; doi:10.1371/journal.pone.0245481)
Supplement: S7 Table — (DOCX) [file pone.0245481.s007.docx]

**S7 Table. Changes from baseline in HbA1c and fasting blood glucose after treatment in
non-diabetic patient with low risk of diabetes (BMI <30kg/m^2^ & fasting glucose <100mg/dL & HbA1c <6%, FA set)**

| **Variable** | **Visit** | **Atorvastatin 10mg (n= 47)** | | | | | **Atorvastatin 20mg (n=49)** | | | | | **Group difference** |
| --- | --- | --- | --- | --- | --- | --- | --- | --- | --- | --- | --- | --- |
|  |  | **Mean (SD)** | **Median** | **Min** | **Max** | **P-value*** | **Mean (SD)** | **Median** | **Min** | **Max** | **P-value*** | **P-value**** |
|  |  |  |  |  |  |  |  |  |  |  |  |  |
| **HbA1c (%)** | Baseline | 5.5 (0.3) | 5.5 | 4.9 | 6.0 |  | 5.5(0.3) | 5.6 | 4.8 | 5.9 |  | 0.9711 |
|  | 12 Week | 5.6(0.3) | 5.6 | 4.9 | 6.4 |  | 5.5(0.3) | 5.6 | 5.0 | 6.2 |  |  |
|  | Change | 0.1 | 0.1 | -0.3 | 0.6 | 0.0243 | 0.1 | 0.1 | -0.3 | 0.5 | 0.0137 |  |
|  |  |  |  |  |  |  |  |  |  |  |  |  |
| **Fasting  glucose  (mg/dL)** | Baseline | 90.6(5.4) | 92.0 | 79.0 | 99.0 |  | 89.1(5.9) | 89.0 | 74.0 | 99.0 |  | 0.9618 |
|  | 12 Week | 92.7(6.2) | 94.0 | 81.0 | 107.0 |  | 91.2(11.0) | 90.0 | 77.0 | 129.0 |  |  |
|  | Change | 2.1 | 1.0 | -10.0 | 20.0 | 0.0530 | 2.02 | 0.0 | -13.0 | 41.0 | 0.1679 |  |

Change: 12 Week-Baseline

*: P-value of paired t-test for the changes from baseline.

**: P-value of Independent t-test for comparison between groups
