# Supplementary material for: A randomized, open-label, parallel, multi-center Phase IV study to compare the efficacy and safety of atorvastatin 10 and 20 mg in high-risk Asian patients with hypercholesterolemia
Source: PLoS One. 2021 Jan 22;16(1):e0245481. doi: 10.1371/journal.pone.0245481 (PMC7822387; doi:10.1371/journal.pone.0245481)
Supplement: S8 Table — (DOCX) [file pone.0245481.s008.docx]

**S8 Table. Changes from baseline in creatine kinase levels after treatment (safety set)**

| Variable | Visit | Total | | Atorvastatin 10mg | | Atorvastatin 20mg | | p-value** |
| --- | --- | --- | --- | --- | --- | --- | --- | --- |
|  |  | (n=249) | | (n=126) | | (n=123) | |  |
|  |  | Mean(SD) | p-value* | Mean(SD) | p-value* | Mean(SD) | p-value* |  |
| Creatine kinase | Baseline | 101.3(57.5) |  | 102.9(64.9) |  | 99.8(49.1) |  | 0.5386 |
|  | 12 Week | 110.1(59.1) |  | 110.3(64.0) |  | 111.6(53.6) |  |  |
|  | Change | 9.5(61.3) | 0.0165 | 7.1(69.7) | 0.2550 | 11.9(51.2) | 0.0123 |  |

Change: 12 Week-Baseline

*: p-value of paired t-test for the changes from baseline.

**: p-value of Independent t-test for comparison between groups
