# Supplementary material for: A randomized, open-label, parallel, multi-center Phase IV study to compare the efficacy and safety of atorvastatin 10 and 20 mg in high-risk Asian patients with hypercholesterolemia
Source: PLoS One. 2021 Jan 22;16(1):e0245481. doi: 10.1371/journal.pone.0245481 (PMC7822387; doi:10.1371/journal.pone.0245481)
Supplement: S1 File — (PDF) [file pone.0245481.s009.pdf]

Clinical Study Protocol

A Randomized, Open-label, Parallel, Multi-Center Phase IV study to Compare of the Efficacy and Safety of Lipilou 20 mg and Lipilou 10 mg in High-risk Patients with Hypercholesterolemia

**Name of Sponsor:**	Chong Kun Dang Pharmaceutical Corp.
**Study Drug:**	Lipilou
**Protocol No.:**	124HL17003
**Version No.:**	1.2 (written date: 02 Apr 2018)
**Effective date**	02 Apr 2018
**Study Phase:**	Phase IV

Since all information related to this document is confidential and proprietary to Chong Kun Dang Pharmaceutical Corp., unauthorized access is prohibited.

Revision History

Version No. (Before change)	Version No. (After change)	Author	Changes and rationales
1.0	1.1	Wooju Lee	Corrected errors and clarified texts
1.1	1.2	Wooju Lee	Changed the study sites

Table of Contents

Abbreviations and Definition of Terms 6
Summary of Study Schedule 7
1 Protocol Synopsis 9
2 Title and Phase of the Study 14
3 Introduction 14
3.1 Background 14
3.2 Rationale 14
3.2.1 Efficacy and Pharmacology Study of Atorvastatin 14
3.2.2 Mechanism of Action of Atorvastatin 15
3.3 Assessment of Benefits[5] 15
3.4 Rationale for the Selection of Doses 15
4 Study Objective 16
5 Study Population 17
5.1 Number of Subjects 17
5.2 Inclusion Criteria 17
5.3 Exclusion Criteria 17
6 Study Design 18
6.1 Study Duration 18
6.2 Group Assignment 18
6.2.1 Randomization 18
6.3 Study Flow Diagram 18
7 Criteria for Completion of Study, Termination of Study, and Early Discontinuation 19
7.1 Criteria for Completion of Study 19
7.2 Criteria for Early Discontinuation 19
7.3 Criteria for Termination of Study 19
8 Identity and Management of the Investigational Products 20
8.1 Identity of the Investigational Products 20
8.1.1 Study Drug: Lipilou Tablet 20 mg 20
8.1.2 Control Drug: Lipilou Tablet 10 mg 20
8.2 Labeling 20
8.3 Packaging 20
8.4 Accountability 20
8.5 Returns and Destruction 20
9 Study Method, Administration Schedule, Etc. 21
9.1 Administration and Treatment Schedule 21
9.1.1 Screening and Wash Out Period: from Weeks -9 to -5 21
9.1.2 Run-in Period: Week -1 21
9.1.3 Treatment period: Weeks 0 to 12 21

9.2 Concomitant Medications 21
9.3 Prohibited Medications 21
9.4 Treatment Compliance 22
10 Study Procedures and Assessments 23
10.1 Visit Schedule and Study Schedule Table 23
10.2 Efficacy Endpoints and Assessments 25
10.2.1 Primary Efficacy Endpoint 25
10.2.2 Secondary Efficacy Endpoints 25
10.2.3 Exploratory Endpoint 25
10.3 Safety Endpoints and Assessments 25
10.3.1 Adverse Event 25
10.3.2 Vital signs 25
10.3.3 Laboratory tests 25
10.4 Adverse Event Reporting 25
10.4.1 Definitions 25
10.4.2 Recording of Adverse Events 26
10.4.3 Assessment of Severity 26
10.4.4 Assessment of Causal Relationship to the Drug 26
10.4.5 Actions Taken for Adverse Events 26
10.4.6 Outcome of Adverse Events 27
10.4.7 Reporting of Serious Adverse Events/Adverse Drug Reactions 27
10.4.8 Follow-Up of Adverse Events 27
10.4.9 Pregnancy during the Study 27
11 Data Analysis and Statistical Considerations 29
11.1 Analysis Sets 29
11.1.1 Full Analysis Set (FAS) 29
11.1.2 Per Protocol Set (PP Set) 29
11.1.3 Safety Analysis Set 29
11.2 Statistical Analysis Method 29
11.2.1 General Principles of Analysis 29
11.2.2 Demographic Information and Other Pre-Treatment Characteristics 29
11.2.3 Statistical Analysis of Efficacy Data 29
11.2.4 Statistical Analysis of Exploratory Data 30
11.2.5 Statistical Analysis of Safety Data 30
11.3 Timing of Analysis and Assessment Criteria 30
11.4 Rationale for Determination of Sample Size 30
12 Data Management 32
12.1 Recording and Collection of Data 32
12.2 Data Access 32
12.3 Protection and Retention of Data 32

13 Ethical Considerations and Administrative Procedures 32
13.1 Korean Good Clinical Practice (KGCP) 32
13.2 Informed Consent Procedure 32
13.3 Ethical Compliance 32
13.4 Measures to Protect the Safety of Subjects 32
13.5 Publication of Results 32
13.6 Confidentiality of Patient Records 32
13.7 Quality Control and Quality Assurance 32
13.7.1 Quality Control 32
13.7.2 Quality Assurance 32
14 Sponsor Information, and Name and Title of the Principal Investigator 33
14.1 Sponsor 33
14.2 Name and Title of the Principal Investigator 33
14.2.1 Coordinating Investigator 33
14.2.2 Principal Investigator 33
14.3 Contract Research Organization (CRO, Analysis Center, CRF, and Central Lab) 33
14.3.1 CRO 33
14.3.2 Analysis Center 33
14.3.3 CRF Vendor 33
14.3.4 Central Lab 33
15 References 34

Abbreviations and Definition of Terms

ADR	Adverse Drug Reaction
AE	Adverse Event
ALP	Alkaline Phosphatase
ALT	Alanine Transaminase
AN	Allocation Number
Apo-A1	Apolipoprotein A1
Apo-B	Apolipoprotein B
AST	Aspartate Transaminase
BUN	Blood Urea Nitrogen
CPK(CK)	Creatine Phosphokinase
e-CRF	Electronic Case Report Form
Hb	Hemoglobin
HbA1c	Glycosylated Hemoglobin
Hct	Hematocrit
HDL-C	High Density Lipoprotein Cholesterol
HMG-CoA reductase	3-hydroxy-3-methylglutaryl-coenzyme A reductase
IRB	Institutional Review Board
IWRS	Interactive Web Response System
LDH	Lactate Dehydrogenase
LDL-C	Low Density Lipoprotein Cholesterol
MFDS	Ministry of Food and Drug Safety
NCEP	National Cholesterol Educational Program
RBC	Red Blood Cell
SADR	Serious Adverse Drug Reaction
SAE	Serious Adverse Event
SN	Screening Number
TG	Triglycerides
TLC	Therapeutic Lifestyle Change
TSH	Thyroid Stimulating Hormone
Urine-hCG	Urine Human Chorionic Gonadotropin
WBC	White Blood Cell
γ-GT(GGT)	Gamma Glutamyl Transpeptidase

Summary of Study Schedule

Visit No. / Week (Day) / Action	Screening & Wash out Period	Run-in Period	Treatment Period		
	Visit 1[2-①]	Visit 2[2-②]	Visit 3[2-③]	Visit 4[2-④]	Unscheduled Visit
	From Weeks -9 to -5 (Day -63 to Day -35)	Week -1 (Day -7 to Day -3)	Week 0 (Day 0)	Week 12 Day 84 (±4)	
Informed consent and assignment of screening number (SN)[1]	●				
Discontinuation of lipid lowering agents[2]	●	●			
Demographic information	●				
Medical history and treatment history[3]	●				
Vital signs[4]	●	●		●	○
Physical examination and body weight	●	●		●	○
Height	●				
Pregnancy test (urine-hCG)[5]	●	●	●	●	○
Laboratory tests[6]	●	●		●	○
Electrocardiography (ECG)	○			○	○
Inclusion/exclusion criteria	●	●			
Assignment of allocation number (AN)[7]			●		
Dispensing of investigational products (IPs)			●		
Collection of returned drugs and assessment of treatment compliance				●	
Adverse event (AE) assessment		●	●	●	●
Concomitant medications[8]	●	●	●	●	●
Training on Therapeutic Lifestyle Change (TLC)[9]	●	●	●	●	○

○: may be performed if deemed necessary by the investigator

1	An SN will be assigned in the order of informed consent.
2	Visit window and duration of discontinuation of lipid lowering agents<br>① Visit 1: Subjects who meet the inclusion/exclusion criteria and require a wash out will have a wash out period as follows.<br>– For fibrates, subjects will have a wash out period of ≥ 8 weeks prior to Visit 2<br>– For agents other than fibrates, subjects will have a wash out period of ≥ 4 weeks prior to Visit 2<br>② Visit 2: After completion of the wash out, subjects will have tests scheduled for Visit 2.<br>③ Visit 3: Subjects who meet the inclusion/exclusion criteria at Visit 2 (or subjects who are drug-naïve at Visit 1 or have not taken fibrates for ≥ 8 weeks or agents other than fibrates for ≥ 4 weeks at screening and meet the inclusion/exclusion criteria) will be dispensed with the IP and take the IP starting from the day of Visit 3.<br>If drug-naïve subjects or subjects who have not taken fibrates for ≥ 8 weeks or agents other than fibrates for ≥ 4 weeks at screening meet the inclusion/exclusion criteria without a wash out period, Visit 2 will be skipped and randomization will be performed at Visit 3 to dispense the IPs.<br>④ Visit 4: The allowed visit window will be 4 days (±4 days) before and after the date specified relative to Visit 3.
3	Medical history (past medical history, surgical history, and current medical history) within 1 year from the date of informed consent will be examined.
4	For vital signs, blood pressure and pulse rate will be measured.
5	A pregnancy test (urine hCG) will be performed only for women of childbearing potential who are not postmenopausal (who have been amenorrheic for at least 12 months) or are not surgically sterile. All pregnancy tests will be performed at the central lab except for Visit 3, where pregnancy will be verified immediately using a pregnancy test kit provided by the central lab and the result will be recorded in the source document.
6	Hematology, blood chemistry, urinalysis, thyroid function test, and lipid parameters<br>All tests will be performed at the central lab in a fasting state (fasting for at least 9 hours) to exclude food effect. Subjects who are not in a fasting state should revisit to have the laboratory tests.<br>Also, one re-test will be allowed only at Visits 1 and 2. If a re-test is performed at Visit 2, the run-in period may last until Week -2 (Day -14).<br>1. Hematology: WBC with differential count (neutrophil, lymphocyte, monocyte, eosinophil, basophil), RBC, Hb, Hct, platelet<br>2. Blood chemistry: Ca, P, glucose, HbA1c, BUN, uric acid, creatinine, total protein, albumin, total bilirubin, direct bilirubin, AST, ALT, ALP, LDH, γ-GT (GGT), CPK (CK), Na, K, Cl<br>3. Urinalysis: specific gravity, PH, protein (albumin), glucose, ketone, occult blood, urobilinogen, nitrite<br>4. Thyroid function test: TSH, free T4<br>5. Lipid parameters: LDL-C, HDL-C, TG, total cholesterol, Apo-A1, Apo-B, Non-HDL-C/HDL-C ratio, total cholesterol/HDL-C ratio, LDL-C/HDL-C ratio, Apo-B/Apo-A1 ratio
7	Subjects who meet the inclusion/exclusion criteria will be assigned an AN by the interactive web response system (IWRS) in the order of randomization.
8	All concomitant medications within 4 weeks from the date of informed consent will be examined. During the study, any change in concomitant medications since last visit will be examined in detail at every visit and administration of prohibited concomitant medications will be checked for.
9	Subjects will be trained on TLC at the time of informed consent and checked for compliance with the TLC instructions and encouraged to follow them at every visit.

1 Protocol Synopsis

<table>
<tr><td>Title</td><td>A Randomized, Open-label, Parallel, Multi-Center Phase IV study to Compare of the efficacy and safety of Lipilou 20 mg and Lipilou 10 mg in high-risk patients with hypercholesterolemia (PEARL study)</td></tr>
<tr><td>Objectives</td><td>► Primary Objective:
To evaluate the superiority of Lipilou 20 mg in percent change in LDL-C to Lipilou 10 mg by comparing percent changes from baseline in LDL-C at Week 12 between Lipilou 20 mg and Lipilou 10 mg
► Secondary Objectives:
1) To evaluate the percent changes from baseline in lipid parameters at Week 12
2) To evaluate the changes from baseline in HbA1c and glucose at Week 12
3) To evaluate the percentage of subjects achieving the target LDL-C by risk group
4) To evaluate the percentage of subjects achieving the target non-HDL-C by risk group
5) To evaluate the safety based on adverse events (AEs), vital signs, laboratory tests, etc.
► Exploratory Objective:
To evaluate pharmacoeconomics of Lipilou 20 mg and 10 mg at Week 12 from baseline</td></tr>
<tr><td>Coordinating Investigator</td><td>Professor Jin-Won Kim, Cardiology, Korea University Guro Hospital</td></tr>
<tr><td>Study Sites and Principal Investigators</td><td>
<table>
<tr><th>No.</th><th>Affiliation</th><th>Department</th><th>Principal investigator</th></tr>
<tr><td>1</td><td>Korea University Guro Hospital</td><td>Cardiology</td><td>Jin-Won Kim</td></tr>
<tr><td>2</td><td>Korea University Ansan Hospital</td><td>Cardiology</td><td>Woo-Hyuk Song</td></tr>
<tr><td>3</td><td>Dong-A University Hospital</td><td>Cardiology</td><td>Jong-Sung Park</td></tr>
<tr><td>4</td><td>Seoul National University Bundang Hospital</td><td>Cardiology</td><td>Tae-Jin Youn</td></tr>
<tr><td>5</td><td>Sejong General Hospital</td><td>Cardiology</td><td>Ji Bak Kim</td></tr>
<tr><td>6</td><td>Pusan National University Yangsan Hospital</td><td>Cardiology</td><td>Yong-Hyun Park</td></tr>
<tr><td>7</td><td>Ulsan University Hospital</td><td>Cardiology</td><td>Shin-Jae Kim</td></tr>
<tr><td>8</td><td>Wonju Severance Christian Hospital</td><td>Cardiology</td><td>Sung Gyun Ahn</td></tr>
<tr><td>9</td><td>Inje University Ilsan Paik Hospital</td><td>Cardiology</td><td>Joon-Hyung Doh</td></tr>
<tr><td>10</td><td>Myongji Hospital</td><td>Cardiology</td><td>Yun-Hyeong Cho</td></tr>
</table>
</td></tr>
<tr><td>Sponsor</td><td>Chong Kun Dang Pharmaceutical Corp.</td></tr>
<tr><td>Studied Indication</td><td>Primary hypercholesterolemia</td></tr>
</table>

Subject

► Inclusion Criteria

1. Adults aged ≥ 19 years
2. Patients in the following high-risk group or above as per the Korean Guidelines for the Management of Dyslipidemia (Committee for Guidelines for Management of Dyslipidemia, 2015, 3rd edition)
   High-risk group:
   1) Carotid artery disease (confirmed carotid artery stenosis of > 50%)
   2) Abdominal aneurysm
   3) Diabetes mellitus

   Very high-risk group:
   1) Coronary artery disease
   2) Ischemic stroke
   3) Transient cerebral ischemic attack
   4) Peripheral vascular disease
3. At Visit 1 (screening),
   1) If a subject is taking a lipid lowering agent, the subject will have a wash out period only if LDL-C is ≤ 250 mg/dl and TG is ≤ 500 mg/dl
      (Subjects who are taking fibrates will have a wash out period of ≥ 8 weeks prior to Visit 2, and subjects who are taking agents other than fibrates will have a wash out period of ≥ 4 weeks prior to Visit 2)
   2) If a subject is drug-naïve or has not taken fibrates for ≥ 8 weeks or agents other than fibrates for ≥ 4 weeks, then subjects who meet the following criteria will proceed with Visit 3 and be randomized without a wash out (Visit 2 will be skipped).
      ① High-risk group: LDL-C ≥ 100 mg/dl and TG ≤ 500 mg/dl
      ② Very high-risk group: LDL-C ≥ 70 mg/dl and TG ≤ 500 mg/dl
4. If a subject (who had a wash out period at Visit 1) has the following test results at Visit 2, the subject will proceed with Visit 3 and be randomized.
   ① High-risk group: LDL-C ≥ 100 mg/dl and TG ≤ 500 mg/dl
   ② Very high-risk group: LDL-C ≥ 70 mg/dl and TG ≤ 500 mg/dl

► Exclusion Criteria

1. Uncontrolled following diseases
   - Diabetes mellitus (HbA1c > 9% at screening)
   - Hypertension (systolic blood pressure ≥ 180 mmHg or diastolic blood pressure ≥ 110 mmHg at screening)
   - Thyroid dysfunction (TSH ≥ 1.5 x upper limit of normal [ULN] at screening)
2. Severe renal or hepatic impairment, active liver diseases, or serum creatinine, AST, or ALT ≥ 2 x ULN
3. Myopathy such as musculoskeletal disorders or rhabdomyolysis, or CPK (CK) level ≥ 2 x ULN
4. Medical or surgical conditions that may affect absorption, distribution, metabolism, and excretion of the investigational product (IP)
   - History of major gastrointestinal surgeries including gastrectomy, gastrointestinal bypass graft, and gastrointestinal anastomosis (excluding simple appendectomy and hernia repair)
   - History of active inflammatory bowel syndrome within the last 12 months
   - Pancreatic dysfunction (e.g., cholestasis) such as Crohn’s disease and pancreatitis currently requiring treatment, or gastrointestinal/rectal bleeding
5. History of hypersensitivity or allergies to any ingredient of the IP
6. History of drug or alcohol abuse within the last 6 months
7. Patients who do not agree to use contraception (Women who have been amenorrheic for at least 12 months will be considered to be postmenopausal)
8. Patients who took other investigational agent within 30 days prior to the first dose of the IP
9. Patients who are difficult to stop drugs that affect the lipid levels after informed consent or require administration of other hypercholesterolemia drugs during the study
10. Patients who require administration of any prohibited concomitant medication specified in this protocol during participation in the study
11. Hereditary disorders such as galactose intolerance, Lapp lactase deficiency, or glucose-galactose malabsorption because this drug contains lactose

<table>
<tr><td></td><td>12. Inability to participate in the study by law or at the judgment of the investigator</td></tr>
<tr><td>Number of Subjects</td><td>248 subjects (124 subjects per group considering a 20% dropout rate)</td></tr>
<tr><td>Study Design</td><td>A randomized, open-label, multi-center Phase IV study

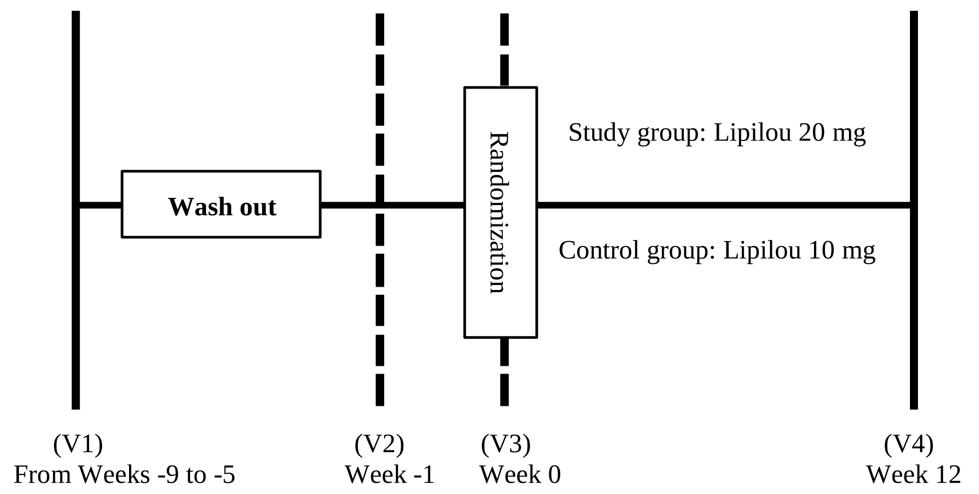

*Wash out is performed only when applicable</td></tr>
<tr><td>Study Duration</td><td>To be completed within 24 months from the date of the IRB approval</td></tr>
<tr><td>Investigational Product</td><td>► Study Drug
- Product name: Lipilou tablet 20 mg
- Ingredient name: Atorvastatin calcium anhydrous
- Manufacturer: Chong Kun Dang Pharmaceutical Corp.
► Control Drug
- Product name: Lipilou tablet 10 mg
- Ingredient name: Atorvastatin calcium anhydrous
- Manufacturer: Chong Kun Dang Pharmaceutical Corp.</td></tr>
<tr><td>Method of Administration and Duration of Administration</td><td>Subjects will be randomized at Visit 3 and take 1 tablet of either the study drug (Lipilou 20 mg) or the control drug (Lipilou 10 mg) as randomized once daily for 12 weeks starting from the day of Visit 3, and are recommended to take the drug at the same time (in the morning) of the day.
*At Visit 4 (out-patient visit), subjects should visit the site without taking the IP. If a subject took the IP, the subject should revisit to have the scheduled tests.</td></tr>
<tr><td>Study Methodology</td><td>1. Screening and wash out period: from Weeks -9 to -5
For subjects who provided informed consent, the following procedures will be performed: assignment of a screening number; examination of demographic information (including risk group assessment), medical history, and treatment history; vital signs (blood pressure and pulse rate) measurement, physical examination, height and body weight measurement, a pregnancy test (urine hCG: only for women of childbearing potential), laboratory tests (hematology, blood chemistry, urinalysis, thyroid function test, and lipid parameters), and ECG (if necessary); assessment of the inclusion/exclusion criteria; discontinuation of lipid lowering agents; checking for concomitant medications; and TLC training.
2. Run-in period: Week -1
For subjects who completed a wash out period, the following procedures will be performed: vital signs (blood pressure and pulse rate) measurement, physical examination, body weight measurement, a pregnancy test (urine hCG: only for women of childbearing potential), laboratory tests (hematology, blood chemistry, urinalysis, thyroid function test, and lipid parameters); checking for concomitant medications and AEs; TLC training; and assessment of the inclusion/exclusion criteria.
* If drug-naïve subjects or subjects who have not taken fibrates for ≥ 8 weeks or agents other than fibrates for ≥ 4 weeks at screening meet the inclusion/exclusion criteria without a wash out period, Visit 2 will be skipped and the IP will be dispensed at Visit 3 and then training on the method of administration will be provided.
3. Treatment period: Weeks 0 to 12</td></tr>
</table>

	3-1. Visit 3 (Week 0): the following procedures will be performed: pregnancy test (urine hCG: only for women of childbearing potential), assignment of allocation number, dispensing of the IPs and training on the method of administration, checking for concomitant medications and AEs, and TLC training<br>3-2. Visit 4 (Week 12): the following procedures will be performed: vital signs (blood pressure and pulse rate) measurement, physical examination, body weight measurement, a pregnancy test (urine hCG: only for women of childbearing potential), laboratory tests (hematology, blood chemistry, urinalysis, thyroid function test, and lipid parameters), and ECG (if necessary); collection of returned drugs and treatment compliance assessment, checking for concomitant medications and AEs, and TLC training
**Endpoints**	► Efficacy<br>1. Primary efficacy endpoint: Percent change in LDL-C at Week 12 from baseline<br>2. Secondary efficacy endpoints:<br>1) Percent changes in lipid parameters at Week 12: LDL-C, HDL-C, TG, total cholesterol, Apo-A1, Apo-B, non-HDL-C/HDL-C ratio, total cholesterol/HDL-C ratio, LDL-C/HDL-C ratio, Apo-B/Apo-A1 ratio<br>2) Changes in HbA1c and glucose at Week 12<br>3) Percentage of subjects achieving the target LDL-C by risk group<br>4) Percentage of subjects achieving the target non-HDL-C by risk group<br>3. Exploratory endpoint: Pharmacoeconomics<br>► Safety<br>AEs, vital signs, laboratory tests, etc.
**Statistical Analysis**	► General Principles of Analysis<br>All statistical analyses in this study will be performed with a two-sided test at a significance level of 5%. If any missing value is found in the efficacy data, the efficacy analysis will be performed applying the last observation carried forward (LOCF), and the analysis of the safety data will be performed using raw data without applying the LOCF.<br>► Demographic Information and Other Pre-Treatment Characteristics<br>The subject's demographic information and pre-treatment characteristics of the IP will be summarized and analyzed by treatment group for the full analysis set (FA Set). For continuous data such as age, descriptive statistics (mean, standard deviation, median, minimum, and maximum) will be presented and the independent samples t-test will be performed for comparison between the treatment groups. For categorial data such as gender and age group, the number and percentage (%) of subjects will be presented and the chi-square test or the Fisher's exact test will be performed for comparison between the treatment groups.<br>Concomitant medications will be classified into prior medications (administration started before the IP administration) and concomitant medications (administration started after the IP administration) and coded using the WHO-ATC Index. The number and percentage (%) of subjects by 1st level of the WHO-ATC Index will be presented, and the chi-square test or the Fisher's exact test will be performed for comparison between the groups.<br>In addition, medical history will be coded using MedDRA version 20.0. The number and percentage (%) of subjects by System Organ Class (SOC) will be presented, and the chi-square test or the Fisher's exact test will be performed for comparison between the groups.<br>► Analysis of the Efficacy Endpoints<br>The efficacy endpoints will be analyzed with the FA Set as the primary analysis set and the Per Protocol Set (PP Set) as the secondary analysis set.<br>1. Primary efficacy endpoint<br>The mean percent change from baseline in LDL-C at Week 12 will be compared between the study group and the control group, and to demonstrate that LDL-C lowering effect in the study group is superior to that in the control group, the independent samples t-test or the Wilcoxon rank sum test will be performed.<br>2. Secondary efficacy endpoints<br>1) The mean percent changes from baseline in lipid parameters at Week 12 will be compared between the study group and the control group using the independent samples t-test or the Wilcoxon rank sum test.<br>* Lipid parameters: HDL-C, TG, total cholesterol, Apo-A1, Apo-B, non-HDL-C/HDL-C ratio, total cholesterol/HDL-C ratio, LDL-C/HDL-C ratio, Apo-B/Apo-A1 ratio

2) The mean change from baseline in HbA1c and glucose at Week 12 will be compared between the study group and the control group using the independent samples t-test or the Wilcoxon rank sum test.

3) The percentage of subjects achieving the target LDL-C by risk group at Week 12 after administration will be set as follows and compared between the study group and the control group using the chi-square test or the Fisher's exact test.

   - Achievement of LDL-C in high-risk group: Percentage (%) of subjects achieving LDL-C < 100 mg/dl
   - Achievement of LDL-C in very high-risk group: Percentage (%) of subjects achieving LDL-C < 70 mg/dl

4) The percentage of subjects achieving the target non-HDL-C by risk group at Week 12 after administration will be set as follows and compared between the study group and the control group using the chi-square test or the Fisher's exact test.

   - Achievement of non-LDL-C in high-risk group: Percentage (%) of subjects achieving non-HDL-C < 130 mg/dl
   - Achievement of non-LDL-C in very high-risk group: Percentage (%) of subjects achieving non-HDL-C < 100 mg/dl

3. Exploratory objective
To evaluate pharmacoeconomics of the study group, an average cost effectiveness ratio (ACER) will be calculated for the percent change from baseline in LDL-C at Week 12 to determine how much cost is added or saved per unit of an improved effect of the study group compared to the control group.

► Analysis of Safety Endpoints

1. Adverse event

Adverse events that occur after administration of the IP will be coded using MedDRA version 20.0. The number of subjects and incidence (%) will be presented by SOC, and the chi-square test or the Fisher's exact test will be performed for comparison between the groups. The AEs that occur in the subjects will be classified into adverse drug reactions (ADRs), serious adverse events (SAEs), and serious adverse drug reactions (SADRs) and analyzed in the same way.

In addition, for unexpected AEs that are not reflected in the labeled information, the number of subjects and incidence (%) will be presented by SOC and Preferred Terms (PT), and the chi-square test or the Fisher's exact test will be performed for comparison between the groups. As with the analysis of the AEs, unexpected AEs will be classified into ADRs, SAEs, and SADRs and analyzed in the same way.

2. Vital signs
Descriptive statistics will be presented by time point, and change from baseline at Week 12 after administration will be compared between the treatment groups using the independent samples t-test or the Wilcoxon rank sum test. The comparison within the treatment group will be performed using the paired samples t-test or Wilcoxon signed rank test.

3. Laboratory tests
The number and percentage (%) of subjects determined to be clinically significant abnormal based on laboratory test results will be presented by treatment group, and comparison between before and after administration will be performed using the McNemar test, and comparison between the treatment groups will be performed using the chi-square test or the Fisher's exact test.

2 Title and Phase of the Study

A. Title: A Randomized, Open-label, Parallel, Multi-Center Phase IV study to Compare of the efficacy and safety of Lipilou 20 mg and Lipilou 10 mg in high-risk patients with hypercholesterolemia (PEARL study)

B. Phase: Phase IV study

3 Introduction

3.1 Background

Dyslipidemia, which is a disease caused by metabolic disorder of lipoprotein, refers to a condition where total cholesterol, LDL-cholesterol, and triglyceride in blood are increased from the reference value or HDL-cholesterol in blood is decreased from the reference value. Dyslipidemia itself does not cause particular symptoms, but if left untreated, cholesterol builds up on the lining of the blood vessels and inflammatory response is caused, leading to formation of plaques around the inflamed area. As a consequence, it causes atherosclerosis, narrowing of the blood vessels. Moreover, if atheromatous plaques become larger in blood vessels with small inner diameter such as carotid artery, which supplies blood to the brain, or coronary artery, which supplies blood to the heart muscles, the risk of vascular stenosis or occlusion increases significantly. When vascular stenosis occurs, symptoms of ischemia first occur in hypoxia-sensitive organs. Ultimately, atherosclerosis causes ischemic cerebrovascular diseases such as cerebral infarction and ischemic heart diseases such as angina pectoris or myocardial infarction.

With regard to the treatment of such dyslipidemia, NCEP ATP III guideline[1] specifies that LDL-C should be controlled primarily for dyslipidemia and that LDL-C treatment goal for the very-high risk group per the risk group classification should be LDL-C < 70 mg/dL at most. The 2013 American College of Cardiology (ACC)/American Heart Association (AHA) Guideline on Dyslipidemia recommends using the percent reduction in LDL-C as an indication of response and adherence to statin therapy instead of the recommendation for LDL-C treatment targets and aims to lower LDL-C by up to ≥ 50% as the guideline on the treatment of blood cholesterol to reduce the risk of atherosclerotic cardiovascular diseases (ASCVDs) in adults.[2] Ultimately, the treatment of dyslipidemia is aiming at lowering LDL-C more intensely.

Atorvastatin, a typical treatment used for dyslipidemia, inhibits HMG-CoA reductase, which is the enzyme that acts on the process of converting HMG-CoA into mevalonic acid, in the rate-limiting step of cholesterol synthesis. This reduces the production of cholesterols in the liver cells, which causes a decreased amount of cholesterols within the cells. As a result, the expression of LDL receptors on the liver cell surface is increased, which in turn greatly reduces cholesterols in the blood and lower the concentration of serum cholesterol.[3]

Commercially available atorvastatin varies in dose, but there are not many researches done to study the cost-effective aspect of different doses,[4] and in Korea, there is a tendency of using atorvastatin 10 mg as the first-line treatment more than atorvastatin 20 mg. A study conducted in Korean dyslipidemia patients demonstrated administration of atorvastatin 20 mg for a daily dose of statin as a first-line treatment as the most successful profile in lowering the LDL-C level to an ideal level, unless high-risk Korean patients have a very high baseline LDL-C level of > 160 mg/dL.4 This result becomes more concrete when the analysis was performed considering the effect as well as the cost, and administration of atorvastatin 20 mg a day is relatively most cost-effective.[4]

Chong Kun Dang Pharmaceutical Corp. intends to provide a beneficial drug to high-risk patients with dyslipidemia by demonstrating that Lipilou 20 mg is more reasonable and cost-effective than Lipilou 10 mg particularly in high-risk patients who need a long-term statin therapy and that there is no significant difference in the incidence of adverse events (AEs), based on comparison between Lipilou 10 mg and 20 mg, atorvastatin agents.

3.2 Rationale

3.2.1 Efficacy and Pharmacology Study of Atorvastatin

1) Efficacy:

In a multi-center, placebo-controlled, dose-finding study in hyperlipidemia patients, it was confirmed that total cholesterol, LDL-C, Apo B, and triglyceride decreased significantly when atorvastatin is administered at a single dose (placebo, 10, 20, 40, and 80 mg) for ≥ 6 weeks.

2) Pharmacology:

- Rhabdomyosarcoma and fibrosarcoma were observed in the muscles of rats administered a high dose of atorvastatin, but the dose was equivalent to approximately 16 times the exposure with oral administration of 80 mg to humans.
- In the high dose groups of male and female mice, an increase in liver adenomas was observed, but the dose was equivalent to approximately 6 times the exposure with oral administration of 80 mg to humans.
- The results of the genetic toxicity test using microorganisms and the micronucleus test using mice were confirmed to be negative.
- When atorvastatin 100 mg/kg/day (approximately 16 times the exposure with oral administration of 80 mg to humans) was administered to rats for 3 months, azoospermia was found in 2 out of 10 rats. When the same

dose was administered to rats for 11 weeks, the movement of sperms and the concentration of sperm heads decreased and the number of abnormal sperms increased.

- When atorvastatin 10, 40, and 120 mg/kg were administered to dogs for 2 years, there was no histopathological AEs in semen parameters and reproductive organs.

3.2.2 Mechanism of Action of Atorvastatin

Atorvastatin inhibits HMG-CoA reductase, which is the enzyme that acts on the process of converting HMG-CoA into mevalonic acid, in the rate-limiting step of cholesterol synthesis. This reduces the production of cholesterols in the liver cells, which causes a decreased amount of cholesterols within the cells. As a result, the expression of LDL receptors on the liver cell surface is increased, which in turn greatly reduces cholesterols in the blood and lower the concentration of serum cholesterol.

3.3 Assessment of Benefits[5]

1. Reduced risk of the following cardiovascular diseases
   1) For adult patients with multiple risk factors for coronary artery heart disease (≥ 55 years of age, smoking, hypertension, low HDL-cholesterol, family history of premature coronary artery heart disease, etc.) in spite of no clinical evidence of coronary artery heart disease,
      (1) Reduced risk of myocardial infarction
      (2) Reduced risk of stroke
      (3) Reduced risk of revascularization and chronic stable angina
   2) For patients with type 2 diabetes mellitus with multiple risk factors of coronary artery heart disease (retinopathy, albuminuria, smoking, hypertension, etc.) in spite of no clinical evidence of coronary artery heart disease,
      (1) Reduced risk of myocardial infarction
      (2) Reduced risk of stroke
   3) For adult patients with clinical evidence of coronary artery heart disease,
      (1) Reduced risk of nonfatal myocardial infarction
      (2) Reduced risk of fatal and nonfatal stroke
      (3) Reduced risk of revascularization
      (4) Reduced risk of hospitalization for congestive heart failure
      (5) Reduced risk of angina pectoris
2. Hyperlipidemia
   1) As an adjunct to diet to reduce elevated total cholesterol, LDL cholesterol, Apo B, and triglyceride levels and to increase HDL cholesterol in adult patients with primary hypercholesterolemia (heterozygous familial and nonfamilial) and mixed dyslipidemia (Fredrickson Types IIa and IIb);
   2) For the treatment of adult patients with primary dysbetalipoproteinemia (Fredrickson Type III) who do not respond adequately to diet
   3) As an adjunct to diet for the treatment of adult patients with elevated serum TG levels (Fredrickson Type IV)
   4) To reduce total cholesterol and LDL cholesterol in patients with homozygous familial hypercholesterolemia as an adjunct to other lip-lowering treatments (e.g., LDL-apheresis) or if such treatments are unavailable

3.4 Rationale for the Selection of Doses

The investigational products (IPs) of this study have been approved by the Ministry of Food and Drug Safety (MFDS). Doses of Lipilou approved by the MFDS are shown below.

Item	Republic of Korea
Strength	10, 20, 40, and 80 mg
Maximum dose	80 mg

This study will use 10 mg and 20 mg of Lipilou among the doses approved by the MFDS to compare and evaluate the efficacy and safety.

4 Study Objective

Only the subjects who meet the inclusion/exclusion criteria will be randomized to either 1 tablet of the study drug (Lipilou 20 mg) or 1 tablet of the control drug (Lipilou 10 mg) and take the doses for 12 weeks for the following objectives.

A. Primary objective:
To evaluate the percent change from baseline in LDL-C at Week 12

B. Secondary objectives:

① To evaluate the percent changes from baseline in lipid parameters at Week 12

② To evaluate the changes from baseline in HbA1c and glucose at Week 12

③ To evaluate the percentage of subjects achieving the target LDL-C by risk group

④ To evaluate the percentage of subjects achieving the target non-HDL-C by risk group

⑤ To evaluate the safety based on AEs, vital signs, laboratory tests, etc.

C. Exploratory objective:
To evaluate pharmacoeconomics of Lipilou 20 mg and 10 mg at Week 12 from baseline

5 Study Population

5.1 Number of Subjects

248 subjects (124 subjects per group)

5.2 Inclusion Criteria

1. Adults aged ≥ 19 years
2. Patients in the following high-risk group or above as per the Korean Guidelines for the Management of Dyslipidemia (Committee for Guidelines for Management of Dyslipidemia, 2015, 3rd edition)
   High-risk group:
   1) Carotid artery disease (confirmed carotid artery stenosis of > 50%)
   2) Abdominal aneurysm
   3) Diabetes mellitus
   Very high-risk group:
   1) Coronary artery disease
   2) Ischemic stroke
   3) Transient cerebral ischemic attack
   4) Peripheral vascular disease
3. At Visit 1 (screening),
   1) If a subject is taking a lipid lowering agent, the subject will have a wash out period only if LDL-C is ≤ 250 mg/dl and TG is ≤ 500 mg/dl
      (Subjects who are taking fibrates will have a wash out period of ≥ 8 weeks prior to Visit 2, and subjects who are taking agents other than fibrates will have a wash out period of ≥ 4 weeks prior to Visit 2)
   2) If a subject is drug-naïve or has not taken fibrates for ≥ 8 weeks or agents other than fibrates for ≥ 4 weeks, then subjects who meet the following criteria will proceed with Visit 3 and be randomized without a wash out (Visit 2 will be skipped).
      ① High-risk group: LDL-C ≥ 100 mg/dl and TG ≤ 500 mg/dl
      ② Very high-risk group: LDL-C ≥ 70 mg/dl and TG ≤ 500 mg/dl
4. If a subject (who had a wash out period at Visit 1) has the following test results at Visit 2, the subject will proceed with Visit 3 and be randomized.
   ① High-risk group: LDL-C ≥ 100 mg/dl and TG ≤ 500 mg/dl
   ② Very high-risk group: LDL-C ≥ 70 mg/dl and TG ≤ 500 mg/dl

5.3 Exclusion Criteria

1. Uncontrolled following diseases
   - Diabetes mellitus (HbA1c > 9% at screening)
   - Hypertension (systolic blood pressure ≥ 180 mmHg or diastolic blood pressure ≥ 110 mmHg at screening)
   - Thyroid dysfunction (TSH ≥ 1.5 x upper limit of normal [ULN] at screening)
2. Severe renal or hepatic impairment, active liver diseases, or serum creatinine, AST, or ALT ≥ 2 x ULN
3. Myopathy such as musculoskeletal disorders or rhabdomyolysis, or CPK (CK) level ≥ 2 x ULN
4. Medical or surgical conditions that may affect absorption, distribution, metabolism, and excretion of the investigational product (IP)
   - History of major gastrointestinal surgeries including gastrectomy, gastrointestinal bypass graft, and gastrointestinal anastomosis (excluding simple appendectomy and hernia repair)
   - History of active inflammatory bowel syndrome within the last 12 months
   - Pancreatic dysfunction (e.g., cholestasis) such as Crohn’s disease and pancreatitis currently requiring treatment, or gastrointestinal/rectal bleeding
5. History of hypersensitivity or allergies to any ingredient of the IP
6. History of drug or alcohol abuse within the last 6 months
7. Patients who do not agree to use contraception (Women who have been amenorrheic for at least 12 months will be considered to be postmenopausal)
8. Patients who took other investigational agent within 30 days prior to the first dose of the IP
9. Patients who are difficult to stop drugs that affect the lipid levels after informed consent or require administration of other hypercholesterolemia drugs during the study
10. Patients who require administration of any prohibited concomitant medication specified in this protocol during participation in the study
11. Hereditary disorders such as galactose intolerance, Lapp lactase deficiency, or glucose-galactose malabsorption because this drug contains lactose
12. Inability to participate in the study by law or at the judgment of the investigator

6 Study Design

6.1 Study Duration

To be completed within 24 months from the date of the IRB approval

6.2 Group Assignment

6.2.1 Randomization

In this study, only the subjects who meet the inclusion criteria will be randomized in a 1:1 ratio. In order to assign subjects to each group in balance, a restricted block randomization method that include study site and risk group result as strata, will be applied, and the block size will be selected among multiples of 2 (e.g., 2, 4, 6, etc.) A randomization table will be created by the biostatistician in charge of randomization using the IWRS, SAS, etc. In order to randomize subjects who meet the inclusion/exclusion criteria and are considered to be eligible to participate in the study based on the risk group information, a stratification variable, randomization will be automatically performed by the IWRS, and the randomization result can be found on the electronic case report form (e-CRF).

When conducting a multi-center study, it is important to standardize the study procedures according to the shared protocol so that the study can be conducted in a similar manner at all sites. Thus, the distribution of subjects should be the same among the study sites to assume that the therapeutic effect would be the same among the study sites.

Once data required to assign an allocation number (AN) are entered on the e-CRF, subjects will be randomized to either the study group or control group by the IWRS. If an AN is erroneously assigned due to errors in the data entry, etc., the already assigned AN cannot be corrected, and the study will proceed as randomized. Subjects enrolled afterwards will be automatically assigned an AN in the order of numbers set in the system.

6.3 Study Flow Diagram

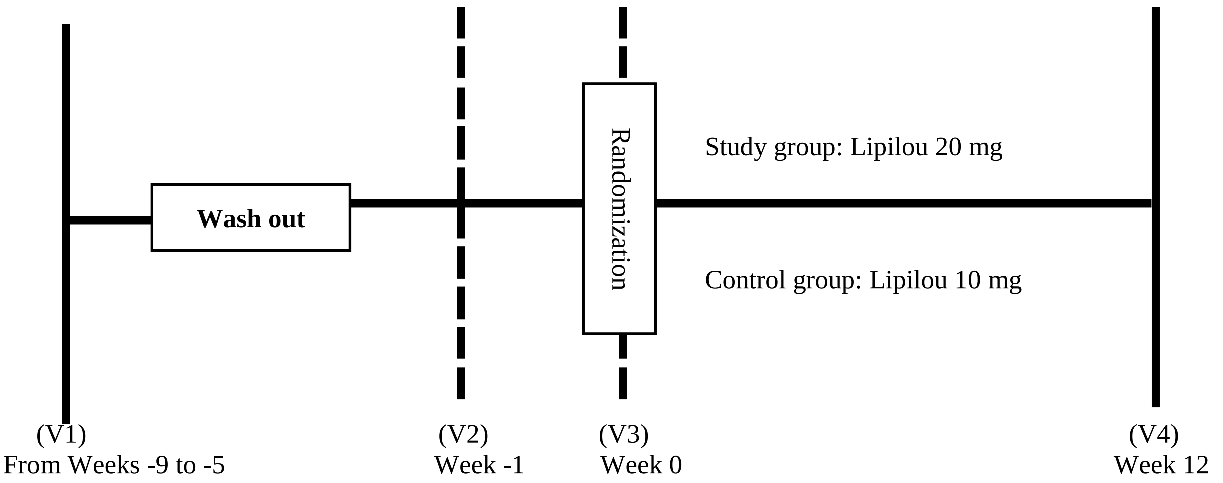

*Wash out is performed only when applicable

7 Criteria for Completion of Study, Termination of Study, and Early Discontinuation

7.1 Criteria for Completion of Study

Subjects who complete all scheduled activities including protocol-specific treatments, tests, and visits will be defined as having completed the study.

7.2 Criteria for Early Discontinuation

If a subject meets any of the following, the subject may be terminated from the study early. In principle, in case of early discontinuation, all tests scheduled for the final visit (Visit 4) will be performed preferably at the last visit date. All abnormalities found in the physical examination and laboratory test results of the subject will be followed up, if possible.

1. A subject is considered difficult to receive further doses of the IP due to AEs
2. A subject is not cooperative during the study
3. A subject or legally acceptable representative voluntarily withdraws consent to continue participation in the study
4. A major protocol deviation is found
5. A subject becomes pregnant during the study
6. A subject is considered ineligible to participate in the study by the investigator for other reasons
7. A subject is lost to follow-up

A subject who experiences any unexpected change during the study must immediately notify the investigator by phone, etc. and be examined by the investigator, if necessary. If an undesirable change occurs to a subject, the investigator may exclude the subject from the study at any time by the subject's voluntary decision or at the judgment of the investigator.

7.3 Criteria for Termination of Study

If a subject shows significant toxicity that is unacceptable to the investigator, the study may be completely terminated for the subject.

8 Identity and Management of the Investigational Products

8.1 Identity of the Investigational Products

8.1.1 Study Drug: Lipilou Tablet 20 mg

1) Generic name: Atorvastatin calcium anhydrous
2) Product name: Lipilou tablet 20 mg
3) Drug substance and its content: Atorvastatin calcium anhydrous 20.720 mg
4) Appearance and dosage form: White, round-shaped film-coated tablet
5) Storage condition: Store in a tight container at room temperature (1 - 30℃)

8.1.2 Control Drug: Lipilou Tablet 10 mg

1) Generic name: Atorvastatin calcium anhydrous
2) Product name: Lipilou tablet 10 mg
3) Drug substance and its content: Atorvastatin calcium anhydrous 10.360 mg
4) Appearance and dosage form: White, round-shaped film-coated tablet
5) Storage condition: Store in a tight container at room temperature (1 - 30℃)

8.2 Labeling

Chong Kun Dang Pharmaceutical Corp. will label the IPs manufactured (or purchased) with a label as shown below (refer to Annex 4-2 [Paragraph 8.4] of the Regulation on the Safety of Pharmaceuticals, etc. and Paragraph 7.7 of the Good Manufacturing Practice for Medicinal Products).

IP No. [Protocol No: 124HL17003]
Investigational Product
1. Product name:<br>2. Batch number:<br>3. Expiry date:<br>4. Storage condition:<br>5. Company name, address, and contact information of the IND holder: Chong Kun Dang Pharmaceutical Corp., 8, Chungjeong-ro, Seodaemun-gu, Seoul, Republic of Korea/Tel: 82-80-6776-080<br>6. Keep away from children.<br>7. Instructions for use:
Instructions for use: Take 1 tablet once daily preferably at the same time (in the morning) of the day

8.3 Packaging

The IPs will be manufactured and packaged by Chong Kun Dang Pharmaceutical Corp. and supplied to the study pharmacists at the study sites in accordance with the 'Regulation on the Safety of Pharmaceuticals, etc.' This study will be an open-label study. The IPs provided during the study will be packaged in a tight container and provided in an open-label manner.

8.4 Accountability

Chong Kun Dang Pharmaceutical Corp. will supply the packaged IPs to the study pharmacists at the study sites. The study pharmacists at the study sites will inspect the IPs upon receipt and record the relevant information in an IP accountability log. Chong Kun Dang Pharmaceutical Corp. should make sure that the IPs are adequately supplied in a timely manner through active communication with the study pharmacists and periodic monitoring.

The pharmacists who manage the IPs (hereinafter, 'study pharmacists') are responsible for receipt, storage, preparation, management, and return of the IPs.

The study pharmacists will acknowledge the receipt and quantity of the IPs in writing by signing and manage them properly. They should ensure that the IPs are administered to the subjects in accordance with the protocol, and accurately record the management of all the IPs provided to each subject.

Once data required to assign an IP number (item number or product number) are entered on the e-CRF, an IP number will be automatically assigned by the IWRS. If an IP number is erroneously assigned due to errors in the data entry, the assignment may be canceled as long as the IP has not been dispensed to the subjects, and an appropriate preventive training will be provided, if necessary.

8.5 Returns and Destruction

Unused IPs will be stored until the sponsor makes a decision on destruction or collection. Upon the end of study, all unused IPs, used/unused containers, labels, and copies of the IP management records will be submitted to the responsible monitor.

In principle, unused IPs or IPs returned by the subjects will be collected and destroyed by Chong Kun Dang Pharmaceutical Corp., and detailed procedures will follow the company's SOP.

9 Study Method, Administration Schedule, Etc.

9.1 Administration and Treatment Schedule

9.1.1 Screening and Wash Out Period: from Weeks -9 to -5

If a subject is taking a lipid lowering agent at screening, the subject will have a wash out period as follows only if LDL-C is ≤ 250 mg/dl and TG is ≤ 500 mg/dl.

- For fibrates, subjects will have a wash out period of ≥ 8 weeks.
- For agents other than fibrates, subjects will have a wash out period of ≥ 4 weeks.

If a subject drug-naïve or has not taken fibrates for ≥ 8 weeks or agents other than fibrates for ≥ 4 weeks at screening, the subject will proceed with Visit 3 and be randomized without a wash out according to the following criteria (Visit 2 will be skipped).

- High-risk group: LDL-C ≥ 100 mg/dl and TG ≤ 500 mg/dl
- Very high-risk group: LDL-C ≥ 70 mg/dl and TG ≤ 500 mg/dl

9.1.2 Run-in Period: Week -1

Subjects with LDL-C ≥ 100 mg/dl and TG ≤ 500 mg/dl in the high-risk group and subjects with LDL-C ≥ 70 mg/dl and TG ≤ 500 mg/dl in the very high-risk group after the wash out will be randomized at Visit 3 and dispensed with the IP.

If drug-naïve subjects or subjects who have not taken fibrates for ≥ 8 weeks or agents other than fibrates for ≥ 4 weeks at screening meet the inclusion/exclusion criteria without a wash out period, Visit 2 will be skipped and randomization will be performed at Visit 3 to dispense the IPs.

9.1.3 Treatment period: Weeks 0 to 12

Subjects will take 1 tablet of either Lipilou 10 mg or Lipilou 20 mg as randomized once daily for 12 weeks and are recommended to take the drug preferably at the same time (in the morning) of the day.

At Visit 4 (Week 12; out-patient visit), subjects should visit the site without having taken the IP and complete all tests scheduled for that visit. If a subject took the IP, the subject should revisit to have the scheduled tests.

9.2 Concomitant Medications

Drugs that are not specified in Section 9.3 Prohibited Medications and are considered to have no effect on the interpretation of the study results may be taken or administered at the judgment of the investigator, considering the subject's condition. Concomitant medications taken or administered during the study must be recorded on the e-CRF.

9.3 Prohibited Medications

The following medications will be prohibited during the study:

1) Drugs that lower lipid levels
   ① Statins, fibrates, ezetimibe, bile acid sequestrants, niacin, anti-obesity drugs, systemic steroids, fish oil, colestine products, fiber-based laxatives, phytosterol margarines, etc. (topical/external steroids may be concomitantly used)
2) Drugs that increase the frequency of side effects or cause abnormal plasma concentration when used concomitantly
   ① CYP3A4 inhibitors (cyclosporine, macrolide antibiotics, azole antifungals, and protease inhibitors): erythromycin, atazanavir, clarithromycin, indinavir, itraconazole, ketoconazole, nefazodone, telaprevir, tipranovir+ritonavir combination, lopinavir+ritonavir combination, saquinavir+ritonavir combination, fosamprenavir, darunavir+ritonavir combination, fosamprenavir+ritonavir combination, etc.
   ② Drugs that affect thyroid function (administration of anti-thyroid agents or thyroxin: subjects on replacement therapy are not applicable)
   ③ CYP3A4 inducers: rifampin, carbamazepine, efavirenz, etc.
   ④ P-glycoprotein inhibitors: cyclosporine, verapamil, and diltiazem
   ⑤ Colestipol
   ⑥ Oral contraceptives containing norethisterone and ethinyl estradiol
   ⑦ Antacids
   ⑧ Grapefruit juice: intake of 1.2 L or more per day is not allowed
   ⑨ Nicotinic acid
   ⑩ Gemfibrozil
3) Other investigational agents

If criteria for temporary or stable use for each agent are met, the following drugs may be used concomitantly. For temporary use, a short-term use within 7 days is allowed, but they must not be taken or administered within 4 days

prior to a scheduled visit. For stable use, they may be taken or administered as long as their dosage is not changed from the time of informed consent until the end of the study.

- Drugs allowed for temporary use: systemic steroids and antacids
- Drugs allowed for stable use: P-glycoprotein inhibitors, oral contraceptives, antacids, and drugs that affect thyroid function (administration of anti-thyroid agents or thyroxin: subjects on replacement therapy are not applicable)

Subjects who sign the informed consent form should be trained to follow guidelines on TLC for the treatment of hypercholesterolemia, as much as possible.

9.4 Treatment Compliance

Subjects should bring all the IPs and containers when visiting the study site during the study. The number of returned IPs will be checked at each visit, and the dose taken will be recorded by checking the number of drugs actually taken. If a subject’s dose taken does not correspond with the number of returned IPs, the reason should be recorded. The overall treatment compliance during the treatment period should be ≥ 80%, and subjects with treatment compliance of < 80% or > 120% will be excluded from the Per Protocol Set (PP Set).

$$\text{Treatment compliance (\%)} = \frac{\text{Number of drugs actually taken}}{\text{Number of drugs to be taken during the relevant period}} \times 100$$

10 Study Procedures and Assessments

10.1 Visit Schedule and Study Schedule Table

1) Visit 1: Screening (from Weeks -9 to -5)

① Informed consent and SN assignment:

Patients selected as potential subjects will be provided with the study-related information through the subject information sheet, and, if they agree, they will provide informed consent by writing down their name, signature, and date as a subject.
An SN will be assigned in the order of informed consent in the form of a 5-digit number. The first 2 digits will represent the site number and the rest 3 digits will represent the order of informed consent.
For example, the 5th subject to provide informed consent at site number 8 will be assigned ‘SN08-005’.

② Demographic information and height:

Demographic information (including risk group assessment) such as initials, age, and gender will be examined and height (in cm; rounded to one decimal place) will be measured.

③ Medical history and treatment history, and concomitant medications:

Medical history (past medical history, surgical history, and current medical history) within 1 year and all concomitant medications within 4 weeks from the date of informed consent will be examined.

④ Vital signs: blood pressure and purse rate

⑤ Physical examination and body weight

Head, neck, heart, lung, abdomen, liver, skin, extremities, etc. will be examined and body weight (in kg; rounded to one decimal place) will be measured.

⑥ Pregnancy test

A pregnancy test (urine hCG) will be performed only for women of childbearing potential who are not postmenopausal (who have been amenorrheic for at least 12 months) or are not surgically sterile, and appropriate contraceptive methods will be used throughout the study.

⑦ Laboratory tests – using central lab

Subjects should have laboratory tests in a fasting state (fasting for at least 9 hours) to exclude food effect. Subjects who are not in a fasting state should revisit to have the laboratory tests.

Laboratory tests	Items
Hematology	WBC with differential count (neutrophil, lymphocyte, monocyte, eosinophil, basophil), RBC, Hb, Hct, platelet
Blood chemistry	Ca, P, glucose, HbA1c, BUN, uric acid, creatinine, total protein, albumin, total bilirubin, direct bilirubin, AST, ALT, ALP, LDH, γ-GT (GGT), CPK (CK), Na, K, Cl
Urinalysis	Specific gravity, PH, protein (albumin), glucose, ketone, occult blood, urobilinogen, nitrite
Thyroid function test	TSH, free T4
Lipid parameters	LDL-C, HDL-C, TG, total cholesterol, Apo-A1, Apo-B, non-HDL-C/HDL-C ratio, total cholesterol/HDL-C ratio, LDL-C/HDL-C ratio, Apo-B/Apo-A1 ratio

* One re-test is allowed.

⑧ Electrocardiography (ECG) - will be performed if deemed necessary by the investigator.

12-lead ECG will be used.

⑨ Assessment of inclusion/exclusion criteria and discontinuation of lipid lowering agents

Subjects determined eligible will stop using current antihyperlipidemic agents.

⑩ Training on TLC:

Subjects will be trained on TLC for the treatment of hypercholesterolemia during the study.

2) Visit 2: Run-in period (Week -1)

*If drug-naïve subjects or subjects who have not taken fibrates for ≥ 8 weeks or agents other than fibrates for ≥ 4 weeks at screening meet the inclusion/exclusion criteria without a wash out period, Visit 2 will be skipped.

① Vital signs

Blood pressure and pulse rate will be measured.

② Physical examination and body weight

Head, neck, heart, lung, abdomen, liver, skin, extremities, etc. will be examined and body weight (in kg; rounded to one decimal place) will be measured.

③ Pregnancy test

A pregnancy test (urine hCG) will be performed only for women of childbearing potential who are not postmenopausal (who have been amenorrheic for at least 12 months) or are not surgically sterile, and appropriate contraceptive methods will be used throughout the study.

④ Laboratory tests – using central lab

Subjects should have laboratory tests in a fasting state (fasting for at least 9 hours) to exclude food effect. Subjects who are not in a fasting state should revisit to have the laboratory tests.
* One re-test is allowed. If a re-test is performed, the run-in period may last until Week -2 (Day -14).

⑤ Assessment of AEs and concomitant medications

AEs that occurred and concomitant medications taken since last visit will be examined.

⑥ Training on TLC:

Subjects will be checked if they have been implementing TLC for the treatment of hypercholesterolemia since last visit.

⑦ Discontinuation of lipid lowering agents

Subjects will continue to stop using antihyperlipidemic agents.

⑧ Assessment of inclusion/exclusion criteria

3) Visit 3: Treatment period (Week 0)

① Pregnancy test

A pregnancy test (urine hCG) will be performed only for women of childbearing potential who are not postmenopausal (who have been amenorrheic for at least 12 months) or are not surgically sterile, and appropriate contraceptive methods will be used throughout the study.
* At Visit 3, pregnancy will be verified immediately using a pregnancy test kit provided by the central lab and the result will be recorded in the source document.

② AN assignment

Subjects who finally meet the inclusion/exclusion criteria will be randomized by the IWRS. An AN is a 5-digit number, and the first 2 digits will represent the site number and the rest 3 digits will represent the order of randomization.
For example, the 5th subject to be assigned an AN at site number 8 will be assigned 'AN08-005'.

③ Dispensing of IPs

The IPs to be taken for 12 weeks (doses for 100 days with overage for 16 days included) will be dispensed, and subjects will be recommended to take 1 tablet of the IP once daily at the same time of the day and will take the drug starting from the day of dispensing. Subjects will be instructed not to take the IP on the day of the next visit (Visit 4), and if they took the IP, they should revisit to have the scheduled tests. Subjects will be instructed to bring unused IPs when visiting the site.

④ Assessment of AEs and concomitant medications

AEs that occurred and concomitant medications taken since last visit will be examined.

⑤ Training on TLC:

Subjects will be checked if they have been implementing TLC for the treatment of hypercholesterolemia since last visit.

4) Visit 4: End of study visit (Week 12)

① Vital signs

Blood pressure and pulse rate will be measured.

② Physical examination and body weight

Head, neck, heart, lung, abdomen, liver, skin, extremities, etc. will be examined and body weight (in kg; rounded to one decimal place) will be measured.

③ Pregnancy test

A pregnancy test (urine hCG) will be performed only for women of childbearing potential who are not postmenopausal (who have been amenorrheic for at least 12 months) or are not surgically sterile, and appropriate contraceptive methods will be used throughout the study.

④ Laboratory tests – using central lab

Subjects should have laboratory tests in a fasting state (fasting for at least 9 hours) to exclude food effect. Subjects who are not in a fasting state should revisit to have the laboratory tests.

⑤ ECG - will be performed if deemed necessary by the investigator.
12-lead ECG will be used.

⑥ Collection of returned drugs and assessment of treatment compliance
The IPs dispensed to the subjects at Visit 3 will be collected and treatment compliance will be assessed.

⑦ Assessment of AEs and concomitant medications
AEs that occurred and concomitant medications taken since last visit will be examined.

⑧ Training on TLC:
Subjects will be checked if they have been implementing TLC for the treatment of hypercholesterolemia since last visit.

5) Unscheduled Visit

During the study, subjects may visit the study site and have necessary tests, if necessary (i.e., when an AE occurs or is suspected). The investigator will remind the subjects about this at regular visits and instruct them to contact the investigator immediately if an AE occurs. Unscheduled visits will be recorded on the e-CRF.

10.2 Efficacy Endpoints and Assessments

10.2.1 Primary Efficacy Endpoint

Percent change from baseline in LDL-C at Week 12

10.2.2 Secondary Efficacy Endpoints

1) Percent changes from baseline in lipid parameters at Week 12
   * Lipid parameters: HDL-C, TG, total cholesterol, Apo-A1, Apo-B, non-HDL-C/HDL-C ratio, total cholesterol/HDL-C ratio, LDL-C/HDL-C ratio, Apo-B/Apo-A1 ratio
2) Changes from baseline in HbA1c and glucose at Week 12
3) Percentage of subjects achieving the target LDL-C by risk group
4) Percentage of subjects achieving the target non-HDL-C by risk group

10.2.3 Exploratory Endpoint

Average cost effectiveness ratio (ACER) for LDL-C at Week 12 from baseline

10.3 Safety Endpoints and Assessments

10.3.1 Adverse Event

1) Incidence of AEs, adverse drug reactions (ADRs), serious adverse events (SAEs), and serious adverse drug reactions (SADRs) that occur throughout the study
2) Incidence of unexpected AEs, ADRs, SAEs, and SADRs

10.3.2 Vital signs

Vital signs results assessed by visit time point

10.3.3 Laboratory tests

Percentage of clinically significant abnormal laboratory results

10.4 Adverse Event Reporting

10.4.1 Definitions

1) An "adverse event (AE)" refers to any unfavorable and unintended sign (including abnormal laboratory test results), symptom, or disease in a subject who received the IP which does not necessarily have to have a causal relationship with the IP.
2) An "adverse drug reaction (ADR)" refers to any unfavorable and unintended response to the IP of any dose, for which causal relationship to the IP cannot be ruled out.
3) A "serious adverse event/adverse drug reaction (SAE/SADR)" refers to an AE or an ADR at any dose of the IP that:
   A. Results in death or is life-threatening
   B. Requires or prolongs hospitalization
   However, hospitalization due to the following reasons is not considered as an SAE:
   - Hospitalization or prolongation of hospitalization for planned surgeries for diagnoses or existing diseases
   - Hospitalization or prolongation of hospitalization required to measure the effect of the study
   - Hospitalization or prolongation of hospitalization for planned treatment of the studied indication
   - Emergency room visit for less than 24 hours
   - Hospitalization for health examination
   C. Results in persistent or significant disability/incapacity
   D. Results in congenital anomalies or birth defects
   E. Important medical events such as the development of drug dependency or drug abuse or blood dyscrasia

4) An "unexpected adverse drug reaction" is any reaction of which the nature or severity of the reaction is not consistent with the available product-related information including the Investigator's Brochure or the package insert of the product.

10.4.2 Recording of Adverse Events

It is the principal investigator's and study staff's responsibility to record all AEs occurring in the study. AEs should be recorded in medical diagnostic terms, and if this is not possible, they should be recorded using the terms for signs and symptoms observed by the principal investigator or the study staff, or reported by subjects.

10.4.3 Assessment of Severity

Severity of AEs will be determined by the principal investigator or the study staff based on their clinical judgment. It will be based on the maximal intensity and can be classified by the criteria below.

Grade	Description	
1	Mild	No special treatment required.<br>Abnormal laboratory test or radiography results, etc. without any symptoms
2	Moderate	Simple, local, or non-invasive treatment, etc.
3	Severe	Hospitalization or invasive treatment such as transfusion, therapeutic endoscopy, or surgery required

10.4.4 Assessment of Causal Relationship to the Drug

The correlation between the administration of the IP and onset of the event will be determined by the principal investigator or the study staff based on their clinical judgment. The criteria for assessment of causal relationship to the IP will be classified as below in accordance with Annex 4-3. Post-marketing Safety Control Standards for Medicinal Products, etc. of the Regulation on the Safety of Pharmaceuticals, etc., and all events except ones assessed as "Unlikely" will be considered to have causal relationship to the IP. Also, for events assessed as "Unlikely", the basis of the decision will be recorded on the e-CRF.

Causal relationship		Basis of decision
1	Certain	The AE follows a reasonable temporal sequence from administration of the IP, not explained by other drugs, chemicals, or accompanying diseases, follows a clinically reasonable response on withdrawal, and is pharmacologically or phenomenologically decisive when such drug, etc. was re-administered as needed
2	Probable/Likely	The AE follows a reasonable temporal sequence from administration of the IP, not explained by other drugs, chemicals, or accompanying diseases, follows a clinically reasonable response on withdrawal (no re-administration information)
3	Possible	The AE follows a reasonable temporal sequence from administration of the IP, but it is also explained by other drugs, chemicals, or accompanying diseases. Also, information about the drug discontinuation is insufficient or unclear
4	Unlikely	It is only a temporary case that does not seem to have a causal relationship with administration of the IP, and it can also be explained by other drugs, chemicals, or accompanying diseases
5	Conditional/Unclassified	For appropriate evaluation, more information is required, or additional data are under review
6	Unassessable/Unclassifiable	A judgment cannot be made because the information is insufficient or contradictory, and the information cannot be supplemented or verified.
7	Not applicable	

10.4.5 Actions Taken for Adverse Events

1) **Action taken with the IP**

No.	Classification
1	Dose increased
2	Dose not changed
3	Dose reduced

4	Drug withdrawn
5	Unknown
6	Not applicable

2) **Other actions**

No.	Classification
1	Concomitant administration of treatment medications
2	Non-medication treatment
3	Not applicable
4	Unknown

10.4.6 Outcome of Adverse Events

No.	Classification
1	Recovered
2	Recovering
3	Not recovered
4	Recovered with sequelae
5	Fatal injury
6	Unknown
7	Death possibly correlated with the AE
8	Death unrelated to the AE

10.4.7 Reporting of Serious Adverse Events/Adverse Drug Reactions

During this study, the principal investigator and the study staff should ensure the safety of subjects and minimize AEs by taking an appropriate measure promptly in the event of any SAEs.
If an SAE occurs during the study, the obligations of each staff member are as follows.

1) Obligations of the principal investigator

If an SAE occurs during the study, it must be reported to the Institutional Review Board (IRB) in accordance with the standard operating procedures (SOP) of the IRB at each study site. The principal investigator or the study staff must report it to Chong Kun Dang Pharmaceutical Corp. within 24 hours or no later than the next business day, regardless of its relationship to the IP. If necessary, a further report including detailed information should be submitted within 7 days to the PV team of Chong Kun Dang Pharmaceutical Corp.

- Tel: 82-2-2194-0425
- Fax: 82-2-2194-0479
- Address: 8, Chungjeong-ro, Seodaemun-gu, Seoul, Republic of Korea
- E-mail address of the PV responsible person at Chong Kun Dang Pharmaceutical Corp.: drugsafety2@ckdpharm.com

2) Obligations of the study staff

The study staff must report to the principal investigator immediately if an SAE occurs during the study.

3) Obligations of the sponsor

The sponsor must report all AEs that occur during the study to the investigators, the IRB, and the president of Korea Institute of Drug Safety & Risk Management within the following timeframes.

① SADRs: within 15 days from the sponsor’s receipt of the report or awareness of the event

② All SAEs that do not constitute ①: within 1 month from the end of each quarter

③ All AEs that do not constitute ① and ②: within 1 month from the end of the quarter where clinical study report submission is due

10.4.8 Follow-Up of Adverse Events

The principal investigator or the study staff should follow the subjects with AEs until the symptoms subside and the abnormal laboratory test results return to the reference value, or a satisfactory explanation for the observed changes is provided. In addition, they should report on the course of the AEs to the staff in charge at Chong Kun Dang Pharmaceutical Corp.

10.4.9 Pregnancy during the Study

Pregnancy during the study will not be considered as an AE, and hospitalization for an elective abortion (excluding

therapeutic abortion) without any complications or normal live birth of a healthy newborn will not be considered as an SAE.

If a subject (or a spouse/partner) becomes pregnant during the study (from the first dose of the IP to 28 days after the last dose), a pregnancy report should be prepared and submitted to the sponsor within 24 hours of becoming aware of the pregnancy.

To collect pregnancy information, however, the patient's signature on the informed consent form for collection of pregnancy information should be obtained first. If a subject becomes pregnant, administration of the study drug will be discontinued immediately, and the subject will be withdrawn from the study.

Even if the subject discontinues participation in the study or is terminated from the study, the sponsor should follow up on and document the progress of pregnancy for both mother and baby until birth.

11 Data Analysis and Statistical Considerations

11.1 Analysis Sets

11.1.1 Full Analysis Set (FAS)

The full analysis set (FAS) means an analysis set that includes all randomized subjects in the analysis and is closest and fully applicable to the intention-to-treat (ITT) principle. Therefore, the FAS will include the subjects that receive at least one dose of the IP after randomization in this study and have at least one efficacy assessment after the IP administration.

11.1.2 Per Protocol Set (PP Set)

The per protocol set (PP Set) will exclude the following subjects considered to have committed a major protocol deviation from the randomized set.

1) Subjects who are terminated from the study early before the protocol-specific time point
2) Subjects who are considered to have committed a major protocol deviation
3) Subjects with overall treatment compliance of < 80% or > 120%

11.1.3 Safety Analysis Set

The safety analysis set will include subjects who are randomized in this study and take at least one dose of the IP after its receipt.

11.2 Statistical Analysis Method

11.2.1 General Principles of Analysis

All statistical analyses in this study will be performed with a two-sided test at a significance level of 5%. If any missing value is found in the efficacy data, the efficacy analysis will be performed applying the last observation carried forward (LOCF), and the analysis of the safety data will be performed using raw data without applying the LOCF.

11.2.2 Demographic Information and Other Pre-Treatment Characteristics

The subject's demographic information and pre-treatment characteristics of the IP will be summarized and analyzed by treatment group for the FA set. For continuous data such as age, descriptive statistics (mean, standard deviation, median, minimum, and maximum) will be presented and the independent samples t-test will be performed for comparison between the treatment groups. For categorial data such as gender and age group, the number and percentage (%) of subjects will be presented and the chi-square test or the Fisher's exact test will be performed for comparison between the treatment groups.

Concomitant medications will be classified into prior medications (administration started before the IP administration) and concomitant medications (administration started after the IP administration) and coded using the WHO-ATC Index. The number and percentage (%) of subjects by 1st level of the WHO-ATC Index will be presented, and the chi-square test or the Fisher's exact test will be performed for comparison between the groups.

In addition, medical history will be coded using MedDRA version 20.0. The number and percentage (%) of subjects by System Organ Class (SOC) will be presented, and the chi-square test or the Fisher's exact test will be performed for comparison between the groups.

11.2.3 Statistical Analysis of Efficacy Data

The efficacy endpoints will be analyzed with the FA Set as the primary analysis set and the PP Set as the secondary analysis set, and for different trends in the analysis results between the two sets, the cause should be identified.

1) Primary efficacy endpoint

   For the mean percent change from baseline in LDL-C at Week 12, descriptive statistics (mean, standard deviation, median, minimum, and maximum) will be presented, and to demonstrate that LDL-C lowering effect in the study group is superior to that in the control group, the independent samples t-test or the Wilcoxon rank sum test will be performed.

2) Secondary efficacy endpoints

   ① For the mean percent changes from baseline in lipid parameters at Week 12, descriptive statistics (mean, standard deviation, median, minimum, and maximum) will be presented, and the mean percent changes will be compared between the study group and the control group using the independent samples t-test or the Wilcoxon rank sum test.

   * Lipid parameters: HDL-C, TG, total cholesterol, Apo-A1, Apo-B, non-HDL-C/HDL-C ratio, total cholesterol/HDL-C ratio, LDL-C/HDL-C ratio, Apo-B/Apo-A1 ratio

   ② For the mean changes from baseline in HbA1c and glucose at Week 12, descriptive statistics (mean, standard deviation, median, minimum, and maximum) will be presented, and the mean changes will be compared between the study group and the control group using the independent samples t-test or the Wilcoxon rank sum test.

   ③ The percentage of subjects achieving the target LDL-Cby risk group at Week 12 after administration will be set as follows and compared between the study group and the control group using the chi-square test or the Fisher's exact test.

- Achievement of LDL-C in high-risk group: Percentage (%) of subjects achieving LDL-C < 100 mg/dl
- Achievement of LDL-C in very high-risk group: Percentage (%) of subjects achieving LDL-C < 70 mg/dl

④ The percentage of subjects achieving the target non-HDL-C by risk group at Week 12 after administration will be set as follows and compared between the study group and the control group using the chi-square test or the Fisher's exact test.

- Achievement of non-LDL-C in high-risk group: Percentage (%) of subjects achieving non-HDL-C < 130 mg/dl
- Achievement of non-LDL-C in very high-risk group: Percentage (%) of subjects achieving non-HDL-C < 100 mg/dl

11.2.4 Statistical Analysis of Exploratory Data

By calculating an ACER using the formula below, how much cost is added or saved per unit of an improved effect of the study group compared to the control group will be explored.

$$\text{ACER} = \frac{mean(C_T - C_N)}{mean(E_T - E_N)}$$

ΔC: difference in the average cost between the study group and the control group,

ΔE: difference in the average effect on LDL−C between the study group and the control group

11.2.5 Statistical Analysis of Safety Data

The safety endpoints will be analyzed on the safety analysis set.

1) Adverse event (AE)
AEs will be coded using MedDRA version 20.0. The number of subjects and incidence (%) will be presented by SOC, and the chi-square test or the Fisher's exact test will be performed for comparison between the groups. The AEs that occur in the subjects will be classified into ADRs, SAEs, and SADRs and analyzed in the same way.
In addition, for unexpected AEs that are not reflected in the labeled information, the number of subjects and incidence (%) will be presented by SOC and Preferred Terms (PT), and the chi-square test or the Fisher's exact test will be performed for comparison between the groups. As with the analysis of the AEs, unexpected AEs will be classified into ADRs, SAEs, and SADRs and analyzed in the same way.
2) Vital signs
For vital signs results, descriptive statistics will be presented by time point, and change will be compared between the treatment groups using the independent samples t-test or the Wilcoxon rank sum test. The comparison within the treatment group will be performed using the paired samples t-test or Wilcoxon signed rank test.
3) Laboratory tests
The number and percentage (%) of subjects determined to be clinically significant abnormal based on laboratory test results will be presented by treatment group, and comparison between before and after administration will be performed using the McNemar test, and comparison between the treatment groups will be performed using the chi-square test or the Fisher's exact test.

11.3 Timing of Analysis and Assessment Criteria

No interim analysis will be performed in this study, and the statistical analysis will be performed after end of the study.

11.4 Rationale for Determination of Sample Size

This study aims to demonstrate that the study group (Lipilou 20 mg) is superior in lowering the percent change in LDL-C at Week 12 to the control group (Lipilou 10 mg) in patients with primary hypercholesterolemia, and to that end, the following hypothesis was set.

<Hypothesis>

$$H_0: \mu_t - \mu_c = 0 \; vs. \; H_1: \mu_t - \mu_c \neq 0$$

μ_t: Percent change from baseline in LDL-C at Week 12 in the study group

μ_c: Percent change from baseline in LDL-C at Week 12 in the control group

To test the above hypothesis, the FDA approval data on rosuvastatin was reviewed, which showed that the percent changes in LDL-C at Week 12 after administration were -50% (±13) for rosuvastatin 20 mg and -44% (±12) for rosuvastatin 10 mg. Thus, the difference between the groups was assumed to be -6%.

% change, mean±sd	Rosuvastatin 10 mg (n=44)	Rosuvastatin 20 mg (n=44)
Baseline	229±45	237±48
Week 12	-44±12	-50±13

Based on the result above, the difference in the percent change between Lipilou 20 mg and Lipilou 10 mg and the standard deviation were assumed and the number of subjects was calculated using the formula below. The difference between the groups and the standard deviation were assumed to be -6 and 13, respectively, and a two-sided significance level of 5% (2.5% for one-sided) with a test power of 90% was applied. The number of subjects calculated was 99 subjects per group, and considering a 20% dropout rate, a total of 248 subjects (124 subjects X 2 groups) need to be enrolled.

$$n = \frac{2 \times (Z_{1-\alpha/2} + Z_{1-\beta})^2 \times \sigma^2}{\epsilon^2} = \frac{2 \times (1.96 + 1.28)^2 \times 13^2}{(-6)^2} = 2 \times \frac{99}{(1-0.2)} \cong 248$$

ϵ = Difference between the groups (−6)

σ^2 = Variance (13^2)

12 Data Management

12.1 Recording and Collection of Data

The investigator should record all data collected in the study on the e-CRF provided by the sponsor. The investigator should record a reasonable explanation for missing data.

The completed CRFs will be finally signed by the principal investigator.

12.2 Data Access

The study monitor, auditors, the IRB, and the Minister of Food and Drug Safety may directly access the data to verify reliability of the study procedures and the data within the limits set by applicable regulations without violating the privacy of subjects.

12.3 Protection and Retention of Data

The investigator should retain the records or documents related to the conduct of this study (e.g., CRFs, informed consent form, clinical study report, other relevant literature, etc.) for 3 years from the date of marketing approval (or the date of study completion for studies unrelated to approval).

13 Ethical Considerations and Administrative Procedures

13.1 Korean Good Clinical Practice (KGCP)

This study will be conducted ethically and scientifically in compliance with the Korean Good Clinical Practice (KGCP) and the basic principles of the Declaration of Helsinki.

13.2 Informed Consent Procedure

Informed consent will be obtained in a separate space in order to protect the subject's identity. The study staff will explain the nature, scope, expected results, etc. of the study to subjects participating in the study in advance by using words that are easy to understand, and sincerely answer various questions from subjects. After having a sufficient time for questions and answers, subjects or their legally acceptable representatives will sign the prepared informed consent form, and so will the study staff.

13.3 Ethical Compliance

This protocol and its amendments will be submitted to the IRB at the study site organized for official approval of the conduct of the study in accordance with the national regulations, and the IRB's decision to on the conduct of the study will be notified to the investigator and the sponsor prior to study initiation in writing.

13.4 Measures to Protect the Safety of Subjects

1) Tests will be performed prior to treatment to strictly determine if subjects are eligible for this study.
2) The study will be conducted according to the protocol, and onset and severity of AEs and ADRs will be assessed through periodic tests and examinations, followed by appropriate measures, during the study.
3) The study sites should be equipped with facilities and specialized personnel necessary for the study as specified in this protocol to properly conduct the study and make every effort to protect the safety of subjects.

13.5 Publication of Results

The study results may be published in an academic journal or presented at an academic conference. However, the sponsor has the right to review the content prior to publication or submission of the study results.

13.6 Confidentiality of Patient Records

The sponsor must provide the right to the protection of personal privacy to subjects. Subject records should be accessible only with subject code numbers and initials, and the data related to the study should be stored in a locked place. However, the principal investigator should give access to the clinical records related to the study in some cases such as a request from a government agency or the sponsor, and if necessary, provide copies of the records, etc. The principal investigator will store the signed informed consent forms until the end of the study and make a list of subject numbers and names to easily find the subjects related records in the future.

13.7 Quality Control and Quality Assurance

13.7.1 Quality Control

The sponsor will monitor the study to ensure that the study is conducted in accordance with the KGCP and recognized when registered at in and outside Korea. During the monitoring, CRFs should be checked for completeness and accuracy and reviewed through comparison in the presence of the study staff, and the study staff should agree that the study monitor or a designee of this task can access to the place for preparation and storage of the IPs and the documents related to the study, and be cooperative. All unused IPs will be collected after the end of the study.

13.7.2 Quality Assurance

The sponsor may perform an audit as part of quality assurance to check if the study was conducted in accordance with the protocol, SOP, and applicable regulations.

14 Sponsor Information, and Name and Title of the Principal Investigator

14.1 Sponsor

1) Name of sponsor: Chong Kun Dang Pharmaceutical Corp.
2) Address: 8, Chungjeong-ro, Seodaemun-gu, Seoul, 03742, Republic of Korea

14.2 Name and Title of the Principal Investigator

14.2.1 Coordinating Investigator

Professor Jin-Won Kim, Cardiology, Korea University Guro Hospital

14.2.2 Principal Investigator

No.	Affiliation	Department	Principal investigator
1	Korea University Guro Hospital	Cardiology	Jin-Won Kim
2	Korea University Ansan Hospital	Cardiology	Woo-Hyuk Song
3	Dong-A University Hospital	Cardiology	Jong-Sung Park
4	Seoul National University Bundang Hospital	Cardiology	Tae-Jin Youn
5	Sejong General Hospital	Cardiology	Ji Bak Kim
6	Pusan National University Yangsan Hospital	Cardiology	Yong-Hyun Park
7	Ulsan University Hospital	Cardiology	Shin-Jae Kim
8	Wonju Severance Christian Hospital	Cardiology	Sung Gyun Ahn
9	Inje University Ilsan Paik Hospital	Cardiology	Joon-Hyung Doh
10	Myongji Hospital	Cardiology	Yun-Hyeong Cho

14.3 Contract Research Organization (CRO, Analysis Center, CRF, and Central Lab)

14.3.1 CRO

Seoul CRO

14.3.2 Analysis Center

Not applicable

14.3.3 CRF Vendor

CRScube

14.3.4 Central Lab

Seoul Clinical Laboratories (SCL)

15 References

1) National Cholesterol Education Program Adult Treatment Panel III (NCEP ATP III) guideline
2) 2013 ACC/AHA Guideline on the Treatment of Blood Cholesterol to Reduce Atherosclerotic Cardiovascular Risk in Adults
3) Clin Pharmacokinet 2003; 42(13): 1141-1160, Clinical Pharmacokinetics of Atorvastatin.
4) Cost-effectiveness of the Use of Statins in the Korean Population 2012; 1(2): 87-94, Journal of Lipid and Atherosclerosis
5) Efficacy and Effects from Lipilou 20 mg Label
6) Guidelines for Management of Dyslipidemia (Committee for Guidelines for Management of Dyslipidemia, 2015, 3rd edition)
7) Guidance on the Clinical Trials Evaluation of Medicinal Products for Hyperlipidemia (February 2008)
8) Guideline on the Clinical Trial of Medicinal Products for Hyperlipidemia (September 2015)
9) Precautions for Use of Atorvastatin
