# Supplementary material for: A randomized, open-label, parallel, multi-center Phase IV study to compare the efficacy and safety of atorvastatin 10 and 20 mg in high-risk Asian patients with hypercholesterolemia
Source: PLoS One. 2021 Jan 22;16(1):e0245481. doi: 10.1371/journal.pone.0245481 (PMC7822387; doi:10.1371/journal.pone.0245481)
Supplement: S2 File — (PDF) [file pone.0245481.s010.pdf]

임상시험계획서

이상지질혈증 고위험군 환자에서 리피로우 20 mg 정 또는 리피로우 10 mg 정 투여할 때의 유효성과 안전성을 평가하기 위한 다기관, 무작위배정, 공개, 평행, 4상 임상시험

A Randomized, Open-label, Parallel, Multi-Center Phase IV study to compare of the efficacy and safety of lipilou 20 mg and lipilou 10 mg in high-risk patients with hypercholesterolemia

**의 뢰 자 명 :**	㈜종근당
**시 험 약 물 명 :**	리피로우
**Protocol No.:**	124HL17003
**Version No.:**	1.2(written date: 2018.04.02)
**Effective date**	2018.04.02
**임상시험단계:**	제 4 상

본 문서와 관련된 모든 정보는 종근당 측의 기밀사항(CONFIDENTIAL)이므로, 관계자외의 접근을 금지합니다.

개정 이력

Version No. (변경전)	Version No. (변경후)	작성자	개정 내용 및 사유
1.0	1.1	이우주	오기정정 및 문구 명확화
1.1	1.2	이우주	실시기관 변경

임상시험계획서 목차

약어 및 용어의 정의 6
임상시험일정 요약 8
1 임상시험 계획서 요약 10
2 임상시험의 제목 및 단계 16
3 서론 16
3.1 배경 16
3.2 이론적 근거 17
3.2.1 Atorvastatin 약효·약리시험 17
3.2.2 Atorvastatin 작용기전 17
3.3 유익성 평가 17
3.4 용량 설정 근거 18
4 시험의 목적 18
5 시험모집단 18
5.1 시험대상자 수 18
5.2 선정기준 18
5.3 제외기준 19
6 시험 설계 내용 20
6.1 임상시험의 기간 20
6.2 군 배정 20
6.2.1 무작위 배정 20
6.3 임상시험의 흐름도 20
7 시험완료, 시험종료 및 조기중단 기준 21
7.1 시험완료 기준 21
7.2 조기중단 기준 21
7.3 시험종료 기준 21
8 임상시험용의약품의 정보 및 관리 21
8.1 임상시험용의약품의 정보 21
8.1.1 시험약: 리피로우정 20 mg 21
8.1.2 대조약: 리피로우정 10 mg 21
8.2 표시기재 21
8.3 포장 22
8.4 수불관리 22
8.5 회수 및 폐기 22
9 시험의 방법 및 투여계획 등 22
9.1 투여 및 치료일정 22
9.1.1 스크리닝 (Screening Period; Wash out): - 8주/-4주 22
9.1.2 치료 전 단계(Run-in Period): -1주 23

9.1.3 치료기(Treatment Period): 0~12주 23
9.2 병용약물 23
9.3 투여금지 약물 23
9.4 치료순응도 24
10 시험의 절차 및 평가 24
10.1 방문일정 및 시험일정표 24
10.2 유효성 평가변수와 평가 27
10.2.1 일차 유효성 평가변수(Primary Endpoint) 27
10.2.2 이차 유효성 평가변수(Secondary Endpoint) 27
10.2.3 탐색적 평가 변수 (Exploratory Endpoint) 27
10.3 안전성 평가변수와 평가 27
10.3.1 이상반응 27
10.3.2 활력징후 27
10.3.3 실험실 검사 28
10.4 이상반응 보고 28
10.4.1 용어의 정의 28
10.4.2 이상반응의 기록 28
10.4.3 심각한 정도의 평가 28
10.4.4 약물과의 인과관계 평가 29
10.4.5 이상반응과 관련하여 취해진 조치 29
10.4.6 이상반응의 결과 30
10.4.7 중대한 이상반응 / 약물이상반응의 보고 30
10.4.8 이상반응의 추적 관찰 31
10.4.9 임상시험 기간 중의 임신 31
11 자료분석 및 통계학적 고려사항 31
11.1 분석군 31
11.1.1 모든 분석 대상자군(Full Analysis Set, 이하 FAS) 31
11.1.2 계획서 순응 임상시험대상자군(Per Protocol Set) 31
11.1.3 안전성 분석 대상자군(Safety Analysis Set) 32
11.2 통계분석방법 32
11.2.1 분석의 일반적 원칙 32
11.2.2 인구학적 정보 및 기타 치료 전 특성 32
11.2.3 유효성 자료의 분석(Statistical Analysis of Efficacy Data) 32
11.2.4 탐색적 평가변수의 분석(Statistical Analysis of Exploratory Data) 33
11.2.5 안전성 자료의 분석(Statistical Analysis of Safety Data) 33
11.3 분석시기 및 판정 기준 33
11.4 시험대상자수 설정근거 33
12 자료관리 34
12.1 자료의 기록 및 수집 34
12.2 자료의 접근 34
12.3 자료의 보호 및 보관 34
13 윤리적 고려사항 및 행정적 절차 34

13.1 임상시험관리기준(KGCP) 34
13.2 시험대상자 동의절차 35
13.3 윤리준수 35
13.4 시험대상자 안전보호 대책 35
13.5 결과발표 35
13.6 환자기록 비밀 유지 35
13.7 품질관리 및 신뢰성 보증 35
13.7.1 품질관리 35
13.7.2 신뢰성 보증 35
14 임상시험 의뢰자의 정보 및 시험책임자의 성명 및 직책 35
14.1 임상시험 의뢰자 35
14.2 시험책임자의 성명 및 직책 36
14.2.1 시험조정자 36
14.2.2 시험책임자 36
14.3 수탁기관(CRO, 분석기관, CRF, Central lab) 36
14.3.1 CRO 36
14.3.2 분석기관 36
14.3.3 CRF 업체 36
14.3.4 Central lab 36
15 참고문헌 37

약어 및 용어의 정의

ADR	Adverse Drug Reaction, 약물이상반응
AE	Adverse Event, 이상반응
ALP	Akaline phosphatase, 혈청 알칼리성 인산분해효소
ALT	Alanine Transaminase, 알라닌 아미노 전달효소
AN	Allocation Number, 무작위배정 번호
Apo-A1	Apolipoprotein A1, 아포지단백 A1
Apo-B	Apolipoprotein B, 아포지단백 B
AST	Aspartate transaminase, 아스파라진산 아미노 전이효소
BUN	Blood Urea Nitrogen, 혈중 요소 질소
CPK(CK)	Creatine Phosphokinase, 크레아틴인산효소
e-CRF	Electronic Case Report Form, 전자증례기록서
Hb	Hemoglobin, 혈색소
HbA1c	Glycosylated hemoglobin, 당화혈색소
Hct	Hematocrit, 적혈구 용적율
HDL-C	High Density Lipoprotein Cholesterol, 고밀도 지단백 콜레스테롤
HMG-CoA reductase	3-hydroxy-3-methylglutaryl-coenzyme A reductase, HMG-CoA 환원효소
IRB	Institutional Review Board, 임상시험심사위원회
IWRS	Interactive Web Response System
LDH	Lactate Dehydrogenase, 젖산 탈수소 효소
LDL-C	Low Density Lipoproteinipid Choesterol, 저밀도 지단백 콜레스테롤
MFDS	Ministry of Food and Drug Safety, 식품의약품 안전처
NCEP	National Cholesterol Educational Program, 미국 콜레스테롤 교육 프로그램
RBC	Red Blood Cell, 적혈구
SADR	Serious Adverse Drug Reaction, 중대한 약물이상반응
SAE	Serious Adverse Event, 중대한 이상반응
SN	Screening Number, 스크리닝 번호
TG	Triglycerides, 중성지방
TLC	Therapeutic Lifestyle Change, 치료적 생활습관 교정
TSH	Thyroid Stimulating Hormone, 갑상선 자극 호르몬
Urine-hCG	Urine Human Chorionic Gonadotropin, 뇨 중 사람 융모성 성선자극호르몬
WBC	White Blood Cell, 백혈구

γ-GT(GGT)	Gamma Glutamyl Transpeptidase, γ-글루타밀 트랜스펩티드가수분해효소

임상시험일정 요약

Action / Visit No. / Week(day)	스크리닝 (Screening & Wash out Period)	치료 전 단계 (Run-in Period)	치료기(Treatment period)		
	Visit 1[2-①]	Visit 2[2-②]	Visit 3[2-③]	Visit 4[2-④]	Unscheduled Visit
	-9~-5Week (D-63 ~ D-35)	-1Week (D-7 ~ D-3)	0Week (D0)	12Week D84(±4)	
문서 동의 및 Screening 번호(SN) 부여[1]	●				
지질저하제 복용 중지[2]	●	●			
인구학적 정보 조사	●				
병력 및 치료력 조사[3]	●				
활력징후[4]	●	●		●	O
신체검사 및 체중	●	●		●	O
신장	●				
임신 반응 검사(Urine-hCG)[5]	●	●	●	●	O
실험실 검사[6]	●	●		●	O
심전도 검사(ECG)	O			O	O
선정/제외기준 확인	●	●			
배정 번호(AN) 부여[7]			●		
임상시험용의약품 처방			●		
반납약 회수 및 순응도 평가				●	
이상반응 평가		●	●	●	●
병용약물 확인[8]	●	●	●	●	●
생활습관교정(TLC) 교육[9]	●	●	●	●	O

O: 연구자 판단에 따라 필요한 경우 실시 가능한 항목

1	문서 동의 순서에 따라 스크리닝 번호를 부여한다.
2	Visit window 및 지질저하제 복용 중단 기간<br>① Visit 1: 선정 및 제외기준을 만족한 대상자 중 Wash out 필요한 대상자는 다음의 기준에 따라 Wash out 기간을 거치도록 한다.<br>–Fibrate 제제의 경우, Visit 2 이전 최소 8 주 이상의 Wash out 기간을 진행한다.<br>–Fibrate 외 제제의 경우, Visit 2 이전 최소 4 주 이상의 Wash out 기간을 진행한다.<br>② Visit 2: Wash out 을 실시하고 Visit 2 에 해당하는 검사를 시행한다.<br>③ Visit 3: Visit 2 선정/제외기준을 만족한 시험대상자의 한하여 (또는 Visit 1 에서 Drug Naïve 이거나 스크리닝 검사 시점에서 Fibrate 제제 8 주 이상, Fibrate 외 제제 4 주 이상 복용 하지 않은 시험대상자 중 선정/제외기준을 만족하는 경우) 임상시험용의약품을 처방하고 Visit 3

	방문일 당일부터 복용한다.<br>단, Drug Naïve 이거나 스크리닝 검사 시점에서 Fibrate 제제 8 주 이상, Fibrate 외 제제 4 주 이상 복용 하지 않은 시험대상자의 경우 Wash out 을 진행하지 않고 선정/제외 기준을 만족하면 Visit 2 를 생략하고 바로 Visit 3 의 무작위배정을 실시하여 임상시험용의약품을 처방한다.<br>④ Visit 4: Visit 3 기준으로 지정된 날짜 전·후 4 일(±4 일)을 Visit window 으로 허용한다.
3	동의 서명일을 기준으로 1 년 이내의 병력(과거 병력, 수술력 및 현재 병력)을 조사한다.
4	활력징후는 혈압과 맥박을 측정한다.
5	월경이 없는 상태가 12개월 이상 지속된 폐경 또는 외과적 불임상태가 아닌 가임기 여성에 한하여 임신반응검사 (Urine hCG)를 시행한다. 모든 임신반응검사는 Central Lab 으로 진행하며, Visit 3만 Central lab에서 제공한 임신반응검사 kit를 사용하여 바로 결과를 확인하고 근거문서에 기록한다.
6	혈액학검사, 혈액화학검사, 뇨검사, 갑상선 기능검사, Lipid parameters<br>모든 검사는 Central Lab으로 진행하며, 식이 영향을 배제하기 위해 공복 상태(최소 9시간 이상의 금식)에서 실시한다. 공복 상태가 아닌 경우 다시 방문하도록 하여 실험실 검사를 실시한다.<br>또한, Visit 1과 Visit 2에 한해 재검 1회를 허용한다. Visit 2 재검을 하는 경우 치료 전 단계가 -2 week (D-14)까지 연장 될 수 있다.<br>1. 혈액학 검사: WBC with differential count(Neutrophil, Lymphocyte, Monocyte, Eosinophil, Basophil), RBC, Hb, Hct, Platelet<br>2. 혈액화학 검사: Ca, P, Glucose, HbA1c, BUN, Uric acid, Creatinine, Total Protein, Albumin, Total Bilirubin, Direct Bilirubin, AST, ALT, ALP, LDH, γ-GT(GGT), CPK(CK), Na, K, Cl<br>3. 뇨검사: Specific Gravity, PH, Protein(Albumin), Glucose, Ketone, Occult Blood, Urobilinogen, Nitrite<br>4. 갑상선기능검사: TSH, Free T4<br>5. Lipid parameters: LDL-C, HDL-C, TG, Total cholesterol, Apo-A1, Apo-B, Non-HDL-C/HDL-C ratio, Total Cholesterol/HDL-C ratio, LDL-C/HDL-C ratio, Apo-B/Apo-A1 ratio
7	선정/제외기준에 적합한 대상자는 무작위 배정 순서대로 IWRS 를 통해 AN 을 부여 한다.
8	동의 서명일을 기준으로 4주 이내의 모든 병용약물을 조사한다. 임상시험 진행 동안, 매 방문 시마다 이전 방문 이후 변화된 병용약물에 대해 자세히 조사하고, 병용금지 약물의 투약 여부를 확인한다.
9	문서 동의를 받으면서 생활습관교정(TLC)에 대해 교육을 시키고, 매 방문마다 교육사항을 잘 이행하고 있는지 확인하며 권고한다.

임상시험 계획서 요약

<table>
<tr><td>제 목</td><td>이상지질혈증 고위험군 환자에서 리피로우 20 mg 정 또는 리피로우 10 mg 정 투여할 때의 유효성과 안전성을 평가하기 위한 다기관, 무작위배정, 공개, 평행, 제 4 상 임상시험

A Randomized, Open-label, Parallel, Multi-Center Phase Ⅳ study to Compare of the efficacy and safety of lipilou 20 mg and lipilou 10 mg in high-risk patients with hypercholesterolemia(PEARL study)</td></tr>
<tr><td>목 적</td><td>► 1 차 목적:
Baseline 으로부터 12 주 후의 LDL-C 의 변화율을 리피로우 20 mg 와 리피로우 10 mg 를 비교하여 리피로우 20 mg 의 LDL-C 의 변화율이 리피로우 10 mg 의 LDL-C 의 변화율에 비해 우월함을 평가하고자 함
► 2 차 목적:
1) Lipid parameters의 Baseline으로부터 12주 후의 변화율 평가
2) HbA1c, Glucose의 Baseline으로부터 12주 후의 변화량 평가
3) 위험군별 LDL-C 목표 도달 비율 평가
4) 위험군별 Non-HDL-C 목표 도달 비율 평가
5) 이상반응, 활력징후, 실험실 검사 등을 통하여 약물의 안전성 평가
►탐색적 목적:
Baseline 으로부터 12 주 후의 리피로우 20 mg 와 10 mg 의 약제 경제성 평가</td></tr>
<tr><td>임상시험 조정자</td><td>고려대학교구로병원 순환기내과 김진원 교수</td></tr>
<tr><td>임상시험실시기관
및
시 험 책 임 자</td><td>
<table>
<tr><th>No.</th><th>소속</th><th>진료과</th><th>시험 책임자</th></tr>
<tr><td>1</td><td>고려대학교구로병원</td><td>순환기내과</td><td>김진원</td></tr>
<tr><td>2</td><td>고려대학교안산병원</td><td>순환기내과</td><td>송우혁</td></tr>
<tr><td>3</td><td>동아대학교병원</td><td>순환기내과</td><td>박종성</td></tr>
<tr><td>4</td><td>분당서울대학교병원</td><td>순환기내과</td><td>연태진</td></tr>
<tr><td>5</td><td>세종병원</td><td>심장내과</td><td>김지박</td></tr>
<tr><td>6</td><td>양산부산대학교병원</td><td>순환기내과</td><td>박용현</td></tr>
<tr><td>7</td><td>울산대학교병원</td><td>심장내과</td><td>김신재</td></tr>
<tr><td>8</td><td>원주세브란스기독병원</td><td>심장내과</td><td>안성균</td></tr>
<tr><td>9</td><td>인제대학교 일산백병원</td><td>순환기내과</td><td>도준형</td></tr>
<tr><td>10</td><td>명지병원</td><td>심장내과</td><td>조윤형</td></tr>
</table>
</td></tr>
<tr><td>의 뢰 자</td><td>㈜종근당</td></tr>
<tr><td>대 상 질 환</td><td>원발성 이상지질혈증 (Hypercholesterolemia)</td></tr>
<tr><td>시 험 대 상 자</td><td>► 선정기준
1. 만 19세 이상 성인
2. 다음과 같은 이상지질혈증 치료지침 (이상지질혈증 치료지침 제정위원회,</td></tr>
</table>

	2015년 제3판 전체본) 에서 정한 고위험군 혹은 그 이상에 해당하는 환자<br>고위험군:<br>1) 경동맥질환 (50%가 넘는 경동맥 협착이 확인된 경우)<br>2) 복부동맥류<br>3) 당뇨병<br>초고위험군:<br>1) 관상동맥질환<br>2) 허혈성 뇌졸중<br>3) 일과성 뇌허혈발작<br>4) 말초혈관질환<br>3. Visit 1(스크리닝 시점)에서 ,<br>1) 지질저하제를 복용하고 있는 경우→ LDL-C ≤ 250 mg/dl, TG ≤ 500 mg/dl 인 환자에 한하여 Wash out 기간 진행<br>(Fibrate 제제는 Visit 2 이전 최소 8 주 이상, Fibrate 외 제제는 Visit 2 이전 최소 4 주 이상 Wash out 진행)<br>2) Drug Naïve 또는 Fibrate 제제 8 주 이상, Fibrate 외 제제 4 주 이상 복용 하지 않은 경우 →<br>① 고위험군: LDL-C ≥ 100 mg/dl, TG ≤ 500 mg/dl 인 환자<br>② 초고위험군: LDL-C ≥ 70 mg/dl, TG ≤ 500 mg/dl 인 환자<br>에 한하여 Wash out 진행하지 않고 Visit 3 및 무작위배정 실시 (Visit 2 생략)<br>4. Visit 2(Visit 1 에서 Wash out 적용한 환자) 검사 결과에서<br>① 고위험군: LDL-C ≥ 100 mg/dl, TG ≤ 500 mg/dl 인 환자<br>② 초고위험군: LDL-C ≥ 70 mg/dl, TG ≤ 500 mg/dl 인 환자에 한하여 Visit 3 및 무작위배정 실시<br>► 제외기준<br>1. 조절되지 않는 아래의 질환이 확인된 환자<br>- 당뇨병(스크리닝 시 HbA1c > 9%)<br>- 고혈압(스크리닝 시 수축기 ≥ 180 mmHg 또는 이완기 ≥ 110 mmHg)<br>- 갑상선 기능 이상(스크리닝 시 TSH 정상상한치 1.5배 이상)<br>2. 중증의 신장애 또는 간장애, 활동성 간질환 환자 또는 Serum Creatinine 수치가 정상상한치 2배 이상, AST 또는 ALT 수치가 정상상한치 2배 이상인 환자<br>3. 골격근 병증, 횡문근융해증 등의 근질환 환자 또는 CPK(CK) 수치가 정상상한치 2배 이상인 환자<br>4. 임상시험용의약품의 흡수, 분포, 대사, 배설에 영향을 줄 수 있는 내과적 또는 외과적 상태에 있는 환자<br>- 위절제, 위장관 우회술 또는 문합술 등 주요 위장관 수술의 병력이 있는 환자(단, 단순 맹장 수술이나 탈장 수술은 제외)<br>- 최근 12개월 이내 활동성 염증성 대장 증후군의 병력이 있는 환자<br>- 현재 치료를 요하는 크론씨병, 췌장염 등의 췌장기능 이상(담즙 정체

	등)이 있거나, 위장관/직장 출혈이 있는 환자<br>5. 임상시험용의약품 성분에 과민증 또는 알러지의 기왕력이 있는 자<br>6. 최근 6개월 이내에 약물 또는 알코올 남용 병력이 있는 환자<br>7. 피임에 동의하지 않는 환자 (단, 최소 12개월 이상 무월경이 지속된 여성의 경우 폐경으로 간주)<br>8. 임상시험용의약품 투여 개시일 기준으로 과거 30일 이내 다른 임상시험용의약품을 복용한 환자<br>9. 동의 서명 후 지질 수치에 영향을 주는 약물 복용 중단이 어렵거나 임상시험 기간 동안 타 이상지질혈증약 복용이 필요한 환자<br>10. 임상시험 참여 기간 중 본 계획서에서 명시한 병용금지약물의 투여가 필요한 환자<br>11. 이 약은 유당을 함유하고 있으므로, 갈락토오스 불내성(galactose intolerance), Lapp 유당분해효소 결핍증(Lapp lactase deficiency) 또는 포도당-갈락토오스 흡수장애(glucose-galactose malabsorption)등의 유전적인 문제가 있는 환자<br>12. 법적으로 임상시험 참여가 불가능하거나 연구자 판단으로 임상시험 참여가 불가능한 환자
**시험대상자 수**	248 명 (군당 124 명, 중도탈락률 20% 반영)
**임상시험 설계**	무작위 배정, 공개, 다기관, 4 상 임상시험<br>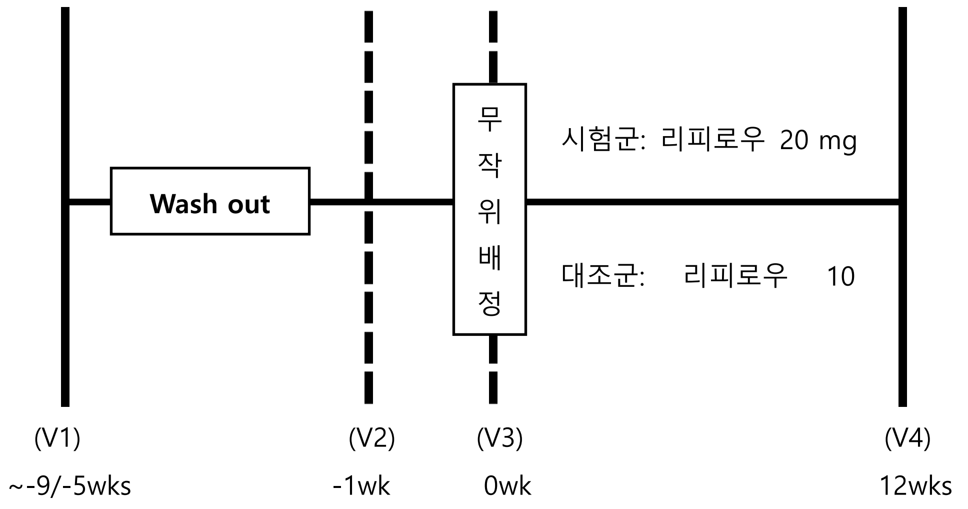<br><br>*Wash out 은 해당하는 경우만 진행
**임상시험 기간**	IRB 승인일로부터 24 개월 이내 종료
**임상시험용의약품**	► 시험약<br>-제품명: 리피로우정 20 mg<br>-성분명: Atorvastatin Calcium Anhydrous<br>-제조사: ㈜종근당<br>► 대조약<br>-제품명: 리피로우정 10 mg<br>-성분명: Atorvastatin Calcium Anhydrous

	-제조사: ㈜종근당
**투여 방법 및 투여 기간**	Visit 3 방문에서 무작위배정이 되며, 무작위배정에 따라 Visit 3 방문 당일부터 12 주 동안 시험약(리피로우 20 mg) 1 정 또는 대조약(리피로우 10 mg) 1 정을 1 일 1 회 투여하며, 동일한 시간(오전 시간)에 투여할 것을 권장함.<br>*단, Visit 4 외래 방문 시에는 임상시험용의약품을 복용하지 않고 방문하도록 한다. 임상시험용의약품을 복용한 경우 다시 방문하도록 하여 계획된 검사를 실시한다.
**시험 방법**	1. 스크리닝 (Screening Period; Wash out): ~-9 주/-5 주<br>문서 동의를 한 시험대상자에게 스크리닝 번호(Screening No., SN)를 부여하고 인구학적 정보(위험군 평가 포함), 병력 및 치료력 조사, 활력징후(혈압, 맥박), 신체검사, 신장 및 체중, 임신반응검사(Urine hCG: 가임여성에 한함), 실험실 검사 (혈액학, 혈액화학, 뇨 검사, 갑상선 기능검사, Lipid parameters), 필요 시 심전도(ECG) 검사, 선정/제외기준 확인, 지질저하제 투여 중지, 병용약물 확인, 생활습관교정(TLC) 교육 실시<br>2. 치료 전 단계 (Run-in Period): -1 주<br>Wash out period 를 진행한 시험대상자는 활력징후(혈압, 맥박), 신체검사, 체중, 임신반응검사(Urine hCG: 가임여성에 한함), 실험실 검사 (혈액학, 혈액화학, 뇨 검사, 갑상선 기능검사, Lipid parameters), 병용약물 및 이상반응 확인, 생활습관교정(TLC) 교육, 선정/제외기준 확인 실시<br>* Drug Naïve 이거나 스크리닝 검사 시점에서 Fibrate 제제 8 주 이상, Fibrate 외 제제 4 주 이상 복용 하지 않은 시험대상자의 경우 Wash out 없이 선정/제외 기준을 만족하면 Visit 2 를 생략하고 바로 Visit 3 에 무작위배정을 실시한 후 임상시험용 의약품 교부 및 투여방법 교육<br>3. 치료기(Treatment Period): 0~12 주<br>3-1. Visit 3 (0 주): 임신반응검사(Urine hCG: 가임여성에 한함), 무작위 배정 번호(Allocation No., AN)부여, 임상시험용 의약품 교부 및 투여방법 교육, 병용약물 및 이상반응 확인, 생활습관교정(TLC) 교육 실시<br>3-2. Visit 4 (12 주): 활력징후(혈압, 맥박), 신체검사, 체중, 임신반응검사(Urine hCG: 가임여성에 한함), 실험실 검사 (혈액학, 혈액화학, 뇨 검사, 갑상선 기능검사, Lipid parameters), 필요 시 심전도(ECG) 검사, 반납약 회수 및 순응도 평가, 병용약물 및 이상반응 확인, 생활습관교정(TLC) 교육 실시
**평가변수**	► 유효성 평가(Efficacy)<br>1. 1차 유효성 평가 변수: LDL-C 변화율<br>2. 2차 유효성 평가 변수:<br>1) Lipid parameters 12주 변화율: LDL-C, HDL-C, TG, Total cholesterol, Apo-A1, Apo-B, Non-HDL-C/HDL-C ratio, Total Cholesterol/HDL-C ratio, LDL-C/HDL-C ratio, Apo-B/Apo-A1 ratio<br>2) HbA1c, Glucose 12주 변화량<br>3) 위험군별 LDL-C 목표 도달 비율<br>4) 위험군별 Non-HDL-C 목표 도달 비율<br>3. 탐색적 평가 변수: 약제 경제성<br>► 안전성 평가(Safety)<br>이상반응, 활력징후, 실험실 검사 등

통 계 분 석	► 분석의 일반적 원칙<br>본 임상시험의 모든 통계분석은 유의수준 5% 하에서 양측검정을 실시함. 유효성 자료에서 결측치가 발생한 경우 Last Observation Carried Forward(이하 LOCF) 방법을 적용하여 유효성 분석을 하고 안전성 자료의 분석은 LOCF 적용 없이 원자료를 그대로 분석함.<br>► 인구학적 정보 및 기타 치료 전 특성<br>시험대상자의 인구학적 정보, 임상시험용의약품 투여 전 특성에 대하여 FA Set 을 대상으로 투여 군별로 요약하고 분석함. 연령 등의 연속형 자료는 평균, 표준편차, 중위수, 최솟값, 최댓값의 기술통계량을 제시하고 투여 군간 비교를 위해 독립 표본 t-검정(Independent Samples t-test)을 실시함. 성별, 연령대와 같은 범주형 자료는 해당하는 시험대상자 수와 비율(%)을 제시하고 투여 군간 비교를 위해 카이제곱검정(Chi-square Test) 또는 피셔의 정확검정(Fisher's Exact Test)을 실시함.<br>병용약물은 임상시험용의약품 투여 이전에 투여 시작한 선행약물과 임상시험용의약품 투여 이후 투여 시작한 병용약물로 구분하여 WHO-ATC Index 에 따라 코딩함. WHO-ATC Index 1st level 에 따라 해당 시험대상자 수와 비율(%)을 제시하고, 카이제곱검정(Chi-square test) 또는 피셔의 정확검정(Fisher's exact test)을 통하여 군간 비교를 확인함.<br>또한, 병력은 MedDRA version20.0 을 사용하여 코딩함. 신체기관계별 분류(SOC; System-Organ Class)에 따라 해당 시험대상자 수와 비율(%)을 제시하고 카이제곱검정(Chi-square test) 또는 피셔의 정확검정(Fisher's exact test)을 통하여 군간 비교를 실시함.<br>► 유효성 평가 변수에 대한 분석<br>유효성 평가변수는 FA Set 을 주 분석 대상군, PP Set 을 보조 분석 대상군으로 설정하여 분석함.<br>1. 1차 유효성 평가 변수<br>Baseline 대비 투여 12주 후의 LDL-C의 평균 변화율에 대하여 시험군과 대조군을 비교하고, 시험군의 LDL-C 강하효과가 대조군의 LDL-C 강하효과에 비해 우월함을 보이기 위해 독립 표본 t-검정(Independent samples t-test) 또는 윌콕슨의 순위합 검정(Wilcoxon rank sum test)를 실시함.<br>2. 2차 유효성 평가 변수<br>1) Baseline 대비 투여 12주 후의 Lipid parameters의 평균 변화율에 대하여 시험군과 대조군의 평균 변화율 비교를 위해 독립 표본 t-검정(Independent samples t-test) 또는 윌콕슨의 순위합 검정(Wilcoxon rank sum test)를 실시함.<br>* Lipid parameters : HDL-C, TG, Total cholesterol, Apo-A1, Apo-B, Non-HDL-C/HDL-C ratio, Total Cholesterol/HDL-C ratio, LDL-C/HDL-C ratio, Apo-B/Apo-A1 ratio<br>2) Baseline 대비 투여 12주 후의 HbA1c, Glucose의 평균 변화량에 대하여 시험군과 대조군의 평균 변화량 비교를 위해 독립 표본 t-검정(Independent samples t-test) 또는 윌콕슨의 순위합 검정(Wilcoxon rank sum test)를 실시함.<br>3) 투여 12주 후 위험군별 LDL-C 도달 비율을 다음과 같이 설정하여 시험군과 대조군간 비교를 위해 카이제곱검정(Chi-square Test) 또는

피셔의 정확검정(Fisher's Exact Test)을 실시함.

- 고위험군 LDL-C 도달: LDL-C 이 100 mg/dl 미만인 시험대상자의 비율(%)

- 초고위험군 LDL-C 도달: 초고위험군 LDL-C이 70 mg/dl 미만인 시험대상자의 비율(%)

4) 투여 12주 후 위험군별 Non-HDL-C 도달 비율을 다음과 같이 설정하여 시험군과 대조군간 비교를 위해 카이제곱검정(Chi-square Test) 또는 피셔의 정확검정(Fisher's Exact Test)을 실시함.

- 고위험군 Non-HDL-C 도달: Non-HDL-C이 130 mg/dl 미만인 시험대상자의 비율(%)

- 초고위험군 Non-HDL-C 도달: Non-HDL-C이 100 mg/dl 미만인 시험대상자의 비율(%)

3. 탐색적 목적

Baseline으로부터 12주 후의 LDL-C 변화율에 대하여 시험군의 약제 경제성 평가를 위하여 ACER(Average Cost Effectiveness Ratio: 비용-효과비)을 산출하여 대조군에 비해 시험군의 증가된 효과 한 단위당 어느 정도의 비용이 추가 또는 절감되는지 확인함.

► 안전성 평가 변수에 대한 분석

1. 이상반응

임상시험용의약품 투여 이후 발현된 이상반응에 대하여 이상반응은 MedDRA version20.0을 사용하여 코딩함. 신체기관계별 분류(SOC; System-Organ Class)에 따라 해당 시험대상자 수와 발현율(%)을 제시하고 카이제곱검정(Chi-square test) 또는 피셔의 정확검정(Fisher's exact test)을 통하여 군간 비교를 실시함. 시험대상자에게 발현한 이상반응은 약물이상반응(ADR), 중대한 이상반응(SAE), 중대한 약물이상반응(SADR)으로 구분하여 동일하게 확인함.

또한, 허가사항에 반영여부를 확인하여 예상하지 못한 이상반응(Unexpected AE)에 대한 신체기관계별 분류(SOC; System-Organ Class)와 우선순위 용어(PT; Preferred Terms)에 따라 해당 시험대상자 수와 발현율(%)을 제시하고 카이제곱검정(Chi-square test) 또는 피셔의 정확검정(Fisher's exact test)을 통하여 군간 비교를 실시함. 이상반응 분석과 동일하게 약물이상반응, 중대한 이상반응, 중대한 약물이상반응으로 구분하여 동일하게 확인함.

2. 활력징후

시점별 기술통계량을 제시하고 baseline 대비 투여 12주 후의 변화량에 대한 투여 군간 비교는 독립 표본 t-검정(Independent Samples t-test) 또는 윌콕슨 순위합 검정(Wilcoxon Rank Sum Test)을 통해 확인함. 투여 군내 비교는 짝지은 표본 t-검정(Paired Samples t-test) 또는 윌콕슨 부호 순위 검정(Wilcoxon Signed Rank Test)을 통해 확인함.

3. 실험실 검사

실험실 검사 결과 임상적으로 의미 있는 비정상으로 판단된 시험대상자 수와 비율(%)을 투여 군별 제시하고 투여 전·후 비교는 맥니마 검정(McNemar Test), 투여 군간 비교는 카이제곱검정(Chi-square Test) 또는 피셔의 정확검정(Fisher's Exact Test)을 통해 확인함

2 임상시험의 제목 및 단계

1) 제목: 이상지질혈증 고위험군 환자에서 리피로우 20 mg 정 또는 리피로우 10 mg 정 투여할 때의 유효성과 안전성을 평가하기 위한 다기관, 무작위배정, 공개, 평행, 제 4 상 임상시험
2) 단계: 4 상 임상시험

3 서론

3.1 배경

이상지질혈증은 지단백의 대사 이상에 의해 발생하는 질환으로, 혈액 중 Total Cholesterol, LDL-Cholesterol, Triglyceride이 기준치보다 증가된 상태거나 HDL-Cholesterol이 기준치보다 감소된 상태를 말한다. 이상지질혈증 자체로 특정 증상이 생기는 것은 아니나, 치료되지 않고 지속된다면 혈관 내벽에 콜레스테롤 등이 침착되고 염증 반응이 일어나게 되어 염증 부위에 플라크가 형성되어 결과적으로 혈관이 협착되는 죽상동맥경화증의 원인이 된다. 더불어 뇌에 혈액을 공급하는 경동맥이나 심장 근육에 혈액을 공급하는 관동맥과 같이 혈관의 내경이 작은 혈관에서 죽상반이 커지게 되면, 혈관 협착이나 폐쇄의 위험성이 유의하게 증가하며, 혈관 협착이 발생하면 저산소증에 민감한 장기에서 허혈 증상이 먼저 발생하므로, 결국 죽상동맥경화증은 뇌경색증과 같은 허혈성 뇌혈관질환 및 협심증 또는 심근경색증과 같은 허혈성 심장질환을 일으키게 된다.

이러한 이상지질혈증의 치료와 관련하여, 미국의 NCEP ATP Ⅲ guideline[1]에서는 이상지질혈증에 1차적으로 LDL-C가 관리되어야 하며, 위험군의 분류에 따른 초고위험군에서는 LDL-C 치료 목표가 최대 LDL-C<70 mg/dL로 관리되어야 한다고 설명하고 있다. 미국심장학회와 심장협회에서 발표한 2013 ACC/AHA 이상지질혈증 가이드라인에서는, 성인의 죽상동맥경화성 심혈관질환 (Atherosclerotic Cardiovascular Disease, ASCVD)의 위험도 감소를 위한 혈중 콜레스테롤 치료 가이드라인으로, LDL-C 치료 목표 수치에 대한 권고안 대신 Statin 치료에 대한 반응이나 순응도를 LDL-C 감소율로 확인하는 것을 권고 하고 있으며, LDL-C 감소폭을 최대 50%이상 감소시키는 것을 목표로 하고 있다.[2] 결과적으로 이상지질혈증의 치료는 보다 강력하게 LDL-C를 감소시키는 방향으로 나아가고 있다.

대표적인 이상지질혈증 치료제로 사용되는 Atorvastatin은 콜레스테롤 합성의 속도조절 단계인 HMG-CoA를 메발론산(mevalonic acid)으로 전환시키는 과정에 작용하는 효소인 HMG-CoA Reductase를 억제 시킨다. 이로 인해, 간세포에서 콜레스테롤의 생산이 감소하여 세포 내의 콜레스테롤 양이 감소하고, 그 결과로 간세포 표면에 LDL 수용체의 발현이 증가하여 혈액 내 콜레스테롤을 많이 제거함으로써 혈청 콜레스테롤 농도를 감소시킨다.[3]

현재 시판중인 Atorvastatin의 용량은 다양하지만, 용량별 비용-효과적인 측면을 검토한 연구는 드물며[4] 한국에서는 Atorvastatin 20 mg보다는 10 mg이 더 많이 최초의 치료로 사용되고 있는 추세이다. 한국인 이상지질혈증 환자를 대상으로 한 연구에서, 고위험군에 속한 한국인 복용자의 LDL-C 기저수치가 160 mg/dL 초과로 매우 높은 경우가 아니라면 Statin의 1일 복용 용량은 Atorvastatin 20 mg을 최초의 치료로 시도되는 것이 LDL-C 수치를 이상적인 수치로 낮추는 데 가장 성공률이 높은 profile로 나타남을 입증하였다.[4] 이러한 결과는 효과뿐만 아니라 비용적인 측면을 고려하여 분석하였을 때 더욱 구체적으로 나타나며, Atorvastatin 1일 20 mg의 복용이 상대적으로 가장 좋은 비용-효과성을 보이고 있다.[4]

(주)종근당에서는 임상시험을 통해 Atorvastatin제제인 리피로우 10 mg과 20 mg을 비교하여, 특히 Statin의 치료가 장기적으로 시행되어야 하는 고위험군 환자에게 리피로우 20 mg이 10 mg보다 합리적이고 비용대비 효과가 좋고, 이상반응 발현율에도 유의한 변화가 없음을 입증하여 고위험군 고지혈증 환자에게 유익한 약제를 제공하고자 한다.

3.2 이론적 근거

3.2.1 Atorvastatin 약효·약리시험

1) 약효:

고지혈증 환자를 대상으로 실시한 다기관, 위약대조, 용량 결정 임상시험에서 Atorvastatin을 단일 용량(placebo, 10, 20, 40, 80 mg)으로 6주 이상 투여 시, Total Cholesterol, LDL-C, Apo B, Triglyceride가 유의하게 감소하는 것을 확인하였다.

2) 약리:

- Atorvastatin을 고용량으로 투여한 랫드의 근육에서 Rhabdomyosarcoma, Fibrosarcoma가 확인 되었으나, 이는 사람에게 80 mg 경구 투여 시, 노출 정도의 약 16배에 해당하는 용량이다.
- 마우스의 암컷과 수컷의 고용량투여군에서 간 선종(liver adenomas)이 증가하는 것으로 관찰 되었으나, 이는 사람에게 80 mg 경구 투여 시, 노출 정도의 약 6배에 해당하는 용량이다.
- 미생물을 이용한 유전 독성 및 마우스를 이용한 소핵시험의 결과는 음성으로 확인 되었다.
- 랫드에 Atorvastatin 100 mg/kg/day(사람에게 80 mg 경구 투여 시, 노출의 약 16배에 해당)를 3개월 투여한 결과 10마리 중 2마리에서 무정자증이 확인 되었고, 동일용량으로 11주간 랫드에 투여 시, 정자의 움직임과 정자 두부의 농도가 감소하고 비정상 정자가 증가하는 것으로 확인 되었다.
- 개에게 2년간 Atorvastatin 10, 40, 120 mg/kg을 투여 했을 때 semen parameters 및 생식기의 조직병리학적 이상반응은 없었다.

3.2.2 Atorvastatin 작용기전

Atorvastatin 은 콜레스테롤 합성의 속도조절 단계인 HMG-CoA 를 메발론산(mevalonic acid)으로 전환시키는 과정에 작용하는 효소인 HMG-CoA Reductase 를 억제 시킨다. 이로 인해, 간세포에서 콜레스테롤의 생산이 감소하여 세포 내의 콜레스테롤 양이 감소하고, 그 결과로 간세포 표면에 LDL 수용체의 발현이 증가하여 혈액 내 콜레스테롤을 많이 제거함으로써 혈청 콜레스테롤 농도를 감소시킨다.

3.3 유익성[5] 평가

1. 다음의 심장혈관 질환에 대한 위험성감소

1) 관상동맥 심장 질환에 대한 임상적 증거는 없으나, 관상동맥 심장 질환의 다중위험요소(55세 이상, 흡연, 고혈압, 낮은 HDL-Cholesterol 또는 조기 관상동맥 심장 질환의 가족력 등)가 있는 성인 환자의

(1) 심근경색증에 대한 위험성 감소

(2) 뇌졸중에 대한 위험성 감소

(3) 혈관재생술 및 만성 안정형 협심증에 대한 위험성 감소

2) 관상동맥 심장 질환에 대한 임상적 증거는 없으나, 관상동맥 심질환의 다중위험요소(망막병증, 알부민뇨, 흡연, 또는 고혈압 등)가 있는 제2형 당뇨병 환자의

(1) 심근경색증에 대한 위험성 감소

(2) 뇌졸중에 대한 위험성 감소

3) 관상동맥 심장 질환에 대한 임상적 증거가 있는 성인 환자의

(1) 비치명적 심근경색증에 대한 위험성 감소

(2) 치명적 및 비치명적 뇌졸중에 대한 위험성 감소

(3) 혈관재생술에 대한 위험성 감소

(4) 울혈심부전으로 인한 입원에 대한 위험성 감소

(5) 협심증에 대한 위험성 감소

2. 고지혈증
   1) 원발성 고콜레스테롤혈증(이형접합 가족형 및 비가족형) 및 혼합형 이상지질혈증(Fredrickson Type Ⅱa 및 Ⅱb형) 환자의 상승된 Total Cholesterol, LDL-Cholesterol, Apo-B, Triglyceride 수치를 감소시키고 HDL-Cholesterol를 증가시키는 식이요법의 보조제
   2) 식이요법에 적절히 반응을 하지 않는 원발성 이상베타리포프로테인혈증 (Fredrickson Type III)
   3) 혈청 Triglyceride가 상승된 환자(Fredrickson Type IV)의 식이요법보조제
   4) 동형접합 가족형 고콜레스테롤혈증 환자의 Total Cholesterol, LDL-Cholesterol를 감소시키기 위해 다른 지질저하제(예, LDL-apheresis)와 병용하거나, 다른 지질저하제로의 치료가 불가능한 경우

3.4 용량 설정 근거

본 임상시험용의약품은 MFDS 에 승인된 의약품들이다. MFDS 에서 승인한 리피로우의 용량은 아래와 같다.

항목	한국
Strength	10, 20, 40, 80 mg
최대용량	80 mg

본 임상시험에서는 MFDS 에서 승인된 리피로우 용량 중 10 mg 과 20 mg 을 사용하여 유효성 및 안전성을 비교 평가할 예정이다.

4 시험의 목적

선정 및 제외 기준을 만족한 시험대상자에 한하여 시험약(리피로우 20 mg) 1 정 또는 대조약(리피로우 10 mg) 1 정에 무작위 배정하여 12 주 동안 투여한 후,

1) 1차 목적:

   Baseline으로부터 12주 후의 LDL-C의 변화율 평가

2) 2차 목적:

   ① Lipid parameters의 Baseline으로부터 12주 후의 변화율 평가
   ② HbA1c, Glucose의 Baseline으로부터 12주 후의 변화량 평가
   ③ 위험군별 LDL-C 목표 도달 비율 평가
   ④ 위험군별 Non-HDL-C 목표 도달 비율 평가
   ⑤ 이상반응, 활력징후, 실험실 검사 등을 통하여 약물의 안전성 평가

3) 탐색적 목적:

   Baseline으로부터 12주 후의 리피로우 20 mg와 10 mg의 약제 경제성 평가

5 시험모집단

5.1 시험대상자 수

248 명(군당 124 명)

5.2 선정기준

1. 만 19세 이상 성인
2. 다음과 같은 이상지질혈증 치료지침 (이상지질혈증 치료지침 제정위원회, 2015년 제3판 전체본)에서 정한 고위험군 혹은 그 이상에 해당하는 환자

고위험군:

1) 경동맥질환 (50%가 넘는 경동맥 협착이 확인된 경우)
2) 복부동맥류
3) 당뇨병

초고위험군:

1) 관상동맥질환
2) 허혈성 뇌졸중
3) 일과성 뇌허혈발작
4) 말초혈관질환

3. Visit 1(스크리닝 시점)에서,
   1) 지질저하제를 복용하고 있는 경우→ LDL-C ≤ 250 mg/dl, TG ≤ 500 mg/dl 인 환자에 한하여 Wash out 기간 진행

      (Fibrate 제제는 Visit 2 이전 최소 8주 이상, Fibrate 외 제제는 Visit 2 이전 최소 4주 이상 Wash out 진행)
   2) Drug Naïve 또는 Fibrate 제제 8 주 이상, Fibrate 외 제제 4 주 이상 복용 하지 않은 경우 →

      ① 고위험군: LDL-C ≥ 100 mg/dl, TG ≤ 500 mg/dl 인 환자

      ② 초고위험군: LDL-C ≥ 70 mg/dl, TG ≤ 500 mg/dl 인 환자

      에 한하여 Wash out 진행하지 않고 Visit 3 및 무작위배정 실시 (Visit 2 생략)
4. Visit 2(Visit 1 에서 Wash out 적용한 환자) 검사 결과에서

   ① 고위험군: LDL-C ≥ 100 mg/dl, TG ≤ 500 mg/dl 인 환자

   ② 초고위험군: LDL-C ≥ 70 mg/dl, TG ≤ 500 mg/dl 인 환자

   에 한하여 Visit 3 및 무작위배정 실시

5.3 제외기준

1. 조절되지 않는 아래의 질환이 확인된 환자
   - 당뇨병(스크리닝 시 HbA1c > 9%)
   - 고혈압(스크리닝 시 수축기 ≥ 180 mmHg 또는 이완기 ≥ 110 mmHg)
   - 갑상선 기능 이상(스크리닝 시 TSH 정상상한치 1.5배 이상)
2. 중증의 신장애 또는 간장애, 활동성 간질환 환자 또는 Serum Creatinine 2배 이상 이상, AST 또는 ALT 수치가 2배 이상인 환자
3. 골격근 병증, 횡문근융해증 등의 근질환 환자 또는 CPK(CK) 수치가 정상상한치 2배 이상인 환자
4. 임상시험용의약품의 흡수, 분포, 대사, 배설에 영향을 줄 수 있는 내과적 또는 외과적 상태에 있는 환자
   - 위절제, 위장관 우회술 또는 문합술 등 주요 위장관 수술의 병력이 있는 환자(단, 단순 맹장 수술이나 탈장 수술은 제외)
   - 최근 12개월 이내 활동성 염증성 대장 증후군의 병력이 있는 환자
   - 현재 치료를 요하는 크론씨병, 췌장염 등의 췌장기능 이상(담즙 정체 등)이 있거나, 위장관/직장 출혈이 있는 환자
5. 임상시험용의약품 성분에 과민증 또는 알러지의 기왕력이 있는 자
6. 최근 6개월 이내에 약물 또는 알코올 남용 병력이 있는 환자

7. 피임에 동의하지 않는 환자 (단, 최소 12개월 이상 무월경이 지속된 여성의 경우 폐경으로 간주)
8. 임상시험용의약품 투여 개시일 기준으로 과거 30일 이내 다른 임상시험용의약품을 복용한 환자
9. 동의 서명 후 지질 수치에 영향을 주는 약물 복용 중단이 어렵거나 임상시험 기간 동안 타 이상지질혈증약 복용이 필요한 환자
10. 임상시험 참여 기간 중 본 계획서에서 명시한 병용금지약물의 투여가 필요한 환자
11. 이 약은 유당을 함유하고 있으므로, 갈락토오스 불내성(galactose intolerance), Lapp 유당분해효소 결핍증(Lapp lactase deficiency) 또는 포도당-갈락토오스 흡수장애(glucose-galactose malabsorption)등의 유전적인 문제가 있는 환자
12. 법적으로 임상시험 참여가 불가능하거나 연구자 판단으로 임상시험 참여가 불가능한 환자

6 시험 설계 내용

6.1 임상시험의 기간

IRB 승인일로부터 24 개월 이내 종료

6.2 군 배정

6.2.1 무작위 배정

본 임상시험은 선정 기준을 만족하는 시험대상자에 한하여 1:1의 비율로 무작위배정을 실시한다. 각 군에 시험대상자를 균형있게 배정하기 위해 실시기관과 위험군 결과를 층으로 한 제한적 블록 무작위배정법 (Restricted Block Randomization)을 적용하고 블록의 크기는 2의 배수 (예: 2, 4, 6 등) 중에서 설정한다. 무작위배정표는 무작위배정 담당 통계학자가 IWRS 또는 SAS등을 이용하여 생성하며, 선정/제외기준을 만족하고 층화변수인 위험군 정보를 확인한 다음 임상시험 참여에 적합하다고 평가된 시험대상자에게 무작위배정을 부여하기 위하여 IWRS를 통하여 무작위배정이 자동으로 이루어지고 배정된 무작위배정은 e-CRF상에서 확인할 수 있다.

다기관으로 임상시험을 진행할 때에는 공통된 임상시험계획서에 따라 임상시험 절차를 표준화하여 전 기관에서 임상시험을 유사하게 진행하는 것이 중요하므로 기관별로 시험대상자의 분포를 같게 하여 치료 효과가 기관별로 동일하다는 것이 가정되어야 한다.

무작위배정은 배정 번호(Allocation No., 이하 AN) 부여를 위한 자료가 e-CRF 에 입력되면 IWRS 를 통하여 시험군 또는 대조군으로 무작위배정된다. 만약 입력 오류 등의 사유로 AN 이 잘못 부여되었을 경우 한번 부여된 AN 은 수정이 불가능하며 무작위배정된 대로 임상시험을 진행한다. 이후 등재되는 시험대상자는 시스템에 설정된 번호순대로 AN 을 자동 부여한다.

6.3 임상시험의 흐름도

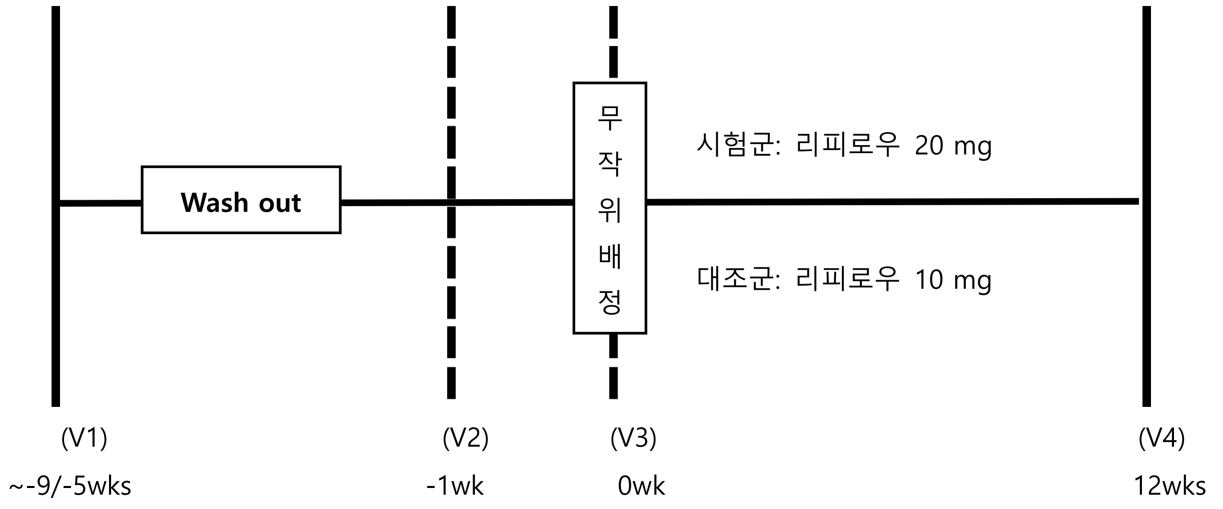

*Wash out 은 해당하는 경우만 진행

7 시험완료, 시험종료 및 조기중단 기준

7.1 시험완료 기준

본 임상시험계획서에서 명시한 모든 투여, 검사, 방문 등 모든 일정을 마친 시험대상자는 임상시험을 완료한 것으로 정의한다.

7.2 조기중단 기준

아래 사항 중 한가지라도 해당되는 시험대상자의 경우 임상시험을 조기종료 할 수 있다. 이전에 임상시험을 중단할 경우, 최종 방문 (Visit 4)에 계획된 모든 검사를 가급적 마지막 방문일에 실시하는 것을 원칙으로 한다. 이 때 해당 시험대상자의 신체 검사, 실험실 검사 등을 통해 발견된 모든 비정상치에 대해서는 가능한 추적 조사한다.

1. 이상반응으로 더 이상 임상약 투여가 힘들다고 판단되는 시험대상자
2. 임상시험 진행 중 비협조적인 시험대상자
3. 시험대상자 또는 대리인이 자발적으로 임상시험 지속 참여를 철회한 경우
4. 중대한 계획서 위반 사항이 발생한 경우
5. 임상시험 진행 중 임신한 시험대상자
6. 기타 연구자의 판단으로 임상시험에 참여가 불가능한 자
7. 임상시험 진행 중 추적 실패한 경우

시험대상자에게 임상시험 도중 예상하지 못했던 변화가 발생하는 경우, 해당 시험대상자는 즉시 시험자에게 전화 등으로 이를 알리고 필요한 경우에 시험자에게 진찰을 받아야 한다. 시험대상자에게 바람직하지 않은 변화가 생겼을 때, 시험자는 해당 시험대상자의 자의 또는 시험자의 판단에 따라 언제라도 시험대상자를 임상시험으로부터 제외시킬 수 있다.

7.3 시험종료 기준

시험자가 받아들일 수 없는 유의한 독성이 나타난 경우 시험을 완전히 종결할 수 있다.

8 임상시험용의약품의 정보 및 관리

8.1 임상시험용의약품의 정보

8.1.1 시험약: 리피로우정 20 mg

1) 일반명: Atorvastatin Calcium Anhydrous
2) 제품명: 리피로우정 20 mg
3) 원료약품 및 그 분량: 무수아토르바스타틴칼슘 20.720 mg
4) 성상 및 제형: 흰색의 원형 필름코팅정제
5) 저장방법: 기밀용기, 실온(1~30℃)보관

8.1.2 대조약: 리피로우정 10 mg

1) 일반명: Atorvastatin Calcium Anhydrous
2) 제품명: 리피로우정 10 mg
3) 원료약품 및 그 분량: 무수아토르바스타틴칼슘 10.360 mg
4) 성상 및 제형: 흰색의 원형필름코팅정제
5) 저장방법: 기밀용기, 실온(1~30℃)보관

8.2 표시기재

㈜종근당은 임상시험용의약품을 제조(또는 구입) 후, 아래의 예시(의약품 등의 안전에 관한 규칙 별표 4 의 2(8.4 항)과 의약품 제조 및 품질관리에 관한 규정의 7.7 항을 참고)와 같은 라벨을 부착한다.

IP번호	[계획서번호: 124HL17003]
임 상 시 험 용 의 약 품	
1. 제품명:	
2. 제조번호:	
3. 사용기한:	
4. 보관조건:	
5. 임상시험계획 승인을 받은 자의 상호와 주소, 전화번호: (주)종근당, 서울특별시 서대문구 충정로 8/ Tel: 080-6776-080	
6. 어린이의 손에 닿지 않는 곳에 보관한다.	
7. 사용방법:	
사용방법: 1일 1회 1정을 가능한 동일한 시간(오전)에 투여	

8.3 포장

임상시험용의약품은 '의약품 등의 안전에 관한 규칙'에 따라 ㈜종근당이 제조, 포장하여 시험기관의 관리약사에게 공급한다. 본 임상시험은 공개(open label)로 진행된다. 임상시험 기간 동안 제공되는 임상시험용의약품은 기밀용기에 포장되어 공개(open label)인 상태로 제공한다.

8.4 수불관리

㈜종근당은 포장한 의약품을 해당 실시기관의 관리약사에게 공급한다. 실시기관의 관리약사는 수령 즉시 임상시험용의약품을 검수하여 확인하고, 해당 내용을 임상시험용의약품 수불기록서에 기재한다. 이때, ㈜종근당은 관리약사와의 원활한 의사소통과 주기적인 모니터링을 통해 임상시험용의약품이 필요한 시기에 적절하게 공급될 수 있도록 한다.

임상시험용의약품 관리약사(이하 '관리약사')는 임상시험용의약품의 인수·보관·조제·관리 및 반납에 대한 책임을 갖는다.

관리약사는 임상시험용의약품의 수령사실 및 수량을 서면으로 확인하고 서명해야 하며, 적절히 관리한다. 임상시험용의약품이 임상시험계획서에 따라서 시험대상자에게 투여되도록 하고, 각 시험대상자에게 지급된 임상시험용의약품 및 관리에 대한 기록을 정확히 한다.

임상시험용의약품 번호(Item No. 또는 제품번호)를 부여하기 위한 필수자료가 e-CRF 에 입력되면 IWRS 를 통하여 임상시험용의약품 번호가 자동으로 부여된다. 만약 자료입력의 오류로 임상시험용의약품 번호가 잘못 배정되었을 경우 시험대상자에게 불출 되지 않은 경우에 한하여 배정을 취소할 수 있고, 필요에 따라 적절한 재발방지 교육을 시행한다.

8.5 회수 및 폐기

사용되지 않은 임상시험용의약품은 파기 또는 회수에 관해 의뢰자가 결정할 때까지 보관하며, 시험 종료 시 모든 미사용 임상시험용의약품, 사용/미사용 용기, 라벨 및 의약품 관리 기록의 복사본을 담당 모니터에게 제출한다.

미사용되거나 시험대상자에게서 반납 받은 임상시험용의약품은 ㈜종근당으로 회수하여 폐기하는 것을 원칙으로 하며, 세부 절차는 ㈜종근당 SOP 의 절차를 따른다.

9 시험의 방법 및 투여계획 등

9.1 투여 및 치료일정

9.1.1 스크리닝 (Screening Period; Wash out): ~- 9 주/-5 주

스크리닝 시 지질저하제를 복용하고 있는 경우 LDL-C ≤ 250 mg/dl, TG ≤ 500 mg/dl 인 환자에 한하여 다음의 기준에 따라 Wash out 기간을 거치도록 한다.

- Fibrate 제제의 경우 8 주 이상의 Wash out 기간을 진행한다.
- Fibrate 외 제제의 경우 4 주 이상의 Wash out 기간을 진행한다.

스크리닝 시 Drug Naïve 또는 Fibrate 제제 8 주 이상, Fibrate 외 제제 4 주 이상 복용 하지 않은 경우 다음의 기준에 따라 Wash out 진행하지 않고 Visit 3 및 무작위배정 실시한다 (Visit 2 생략).

- 고위험군: LDL-C ≥ 100 mg/dl, TG ≤ 500 mg/dl 인 환자
- 초고위험군: LDL-C ≥ 70 mg/dl, TG ≤ 500 mg/dl 인 환자

9.1.2 치료 전 단계(Run-in Period): -1 주

Wash out 후에도 고위험군의 경우 LDL-C ≥ 100 mg/dl, TG ≤ 500 mg/dl 인 시험대상자, 초고위험군의 경우 LDL-C ≥ 70 mg/dl, TG ≤ 500 mg/dl 인 시험대상자에 한하여 Visit 3 에 무작위 배정을 실시하여 임상시험용 의약품을 처방한다.

단, Drug Naïve 이거나 스크리닝 검사 시점에서 Fibrate 제제 8 주 이상, Fibrate 외 제제 4 주 이상 복용 하지 않은 시험대상자의 경우 Wash out 을 진행하지 않고 선정/제외 기준을 만족하면 Visit 2 를 생략하고 바로 Visit 3 에 무작위배정을 실시하여 임상시험용 의약품을 처방한다.

9.1.3 치료기(Treatment Period): 0~12 주

무작위배정에 따라 12 주동안 리피로우 10 mg 1 정 또는 리피로우 20 mg 1 정을 1 일 1 회 투여하며, 가능한 동일한 시간(오전)에 투여할 것을 권고한다.

단, 12 주 Visit 4 외래 방문 시에는 임상시험용의약품을 복용하지 않고 방문하도록 하고, 해당 방문에 계획된 모든 검사를 완료 한다. 임상시험용의약품을 복용한 경우 다시 방문하도록 하여 계획된 검사를 실시한다.

9.2 병용약물

아래 9.3 투여금지약물에 해당되지 않으면서, 시험대상자의 상태를 고려하여 임상시험 결과 해석에 영향을 미치지 않을 것으로 판단되는 의약품은 시험자 판단에 따라 복용 또는 투여가 가능하다. 임상시험 중 복용 또는 투여된 병용 약물은 전자증례기록서(e-CRF)에 반드시 기록한다.

9.3 투여금지 약물

다음의 약물들은 임상시험 기간 동안 투여가 금지된다.

1) 지질 수치 저하에 영향을 미치는 약물

① statins, fibrates, ezetimibe, 담즙산 결합제, niacin, 비만치료제, 전신 작용을 목적으로 하는 스테로이드제제, Fish oil, colestine 제품, fiber-based laxatives, Phytosterol margarines 등
(스테로이드 제제의 경우 국소/외용제제는 병용 가능)

2) 병용 시 부작용의 빈도를 증가시키거나 혈장 농도 이상을 초래하는 경우

① CYP3A4 억제제 (Cyclosporine, 마크로라이드계 항생제, 아졸계 항진균제 및 프로테아제 억제제): Erythromycin, Atazanavir, Clarithromycin, Indinavir, Itraconazole, Ketoconazole, Nefazodone, Telaprevir, Tipranovir+Ritonavir combination, Lopinavir+Ritonavir combination, Saquinavir+, Ritonavir combination, Fosamprenavir, Darunavir+Ritonavir combination, Fosamprenavir+ Ritonavir combination 등

② 갑상선 기능에 영향을 미치는 약물 (갑상선 제제 또는 티록신 투여: 대체요법 중인 사람은 예외)

③ CYP3A4 유도제: Rifampin, Carbamazepine, Efavirenz 등

④ P-글리코프로테인(P-당단백질) 저해제: Cyclosporine, Verapamil, Diltiazem

⑤ 콜레스티폴

⑥ 노르에티스테론 및 에티닐에스트라디올을 함유하는 경구피임제

⑦ 제산제

⑧ 자몽주스: 1일 1.2L 이상 섭취 금지

⑨ 니코틴산

⑩ 겜피브로질

3) 타 임상시험용의약품

단, 제제별로 일시적 또는 안정적 사용 기준을 지킬 경우에는 임상시험 기간 동안 병용할 수 있다. 일시적 사용은 7 일 이내의 단기간 사용은 허용하되 방문 예정일 4 일 이내의 복용 또는 투여는 금지하고, 안정적 사용은 동의서명일부터 임상시험 종료 시점까지 용량의 변경이 없는 경우 복용 또는 투여를 허용한다.

- 일시적 사용 가능 약물: 전신 스테로이드 제제, 제산제
- 안정적 사용 가능 약물: P-당단백질 저해제, 경구피임제, 제산제, 갑상선 기능에 영향을 미치는 약물 (갑상선 제제 또는 티록신 투여: 대체요법 중인 사람은 예외)

자필 동의 서명을 한 모든 시험대상자에게는 가급적 이상지질혈증 치료를 위한 생활습관교정(TLC) 안내문에 따라 생활할 수 있도록 교육해야 한다.

9.4 치료순응도

임상시험 기간동안 시험기관 방문시에는 모든 임상시험용 의약품 및 용기를 가져오도록 한다. 각 방문시마다 반납한 임상시험용 의약품의 개수를 확인하고, 복용량은 시험대상자가 실제로 복용한 약의 개수를 조사하여 기록한다. 시험대상자의 복용량이 반납량과 일치하지 않는 경우 이에 대한 사유를 기록한다. 치료기간 동안 전체 복약 순응도는 80% 이상이어야 하며 복약 순응도가 80% 미만 또는 120% 초과인 경우 해당 시험대상자는 PP 분석군에서 제외한다.

$$\text{복약순응도(\%)} = \frac{\text{실제 복용한 약의 개수}}{\text{해당기간에 복용해야 하는 약의 개수}} \times 100$$

10 시험의 절차 및 평가

10.1 방문일정 및 시험일정표

1) Visit 1: 스크리닝 (~-9/-5 week)

① 문서 동의 및 Screening No.(SN) 부여:

잠정적인 시험대상자로 선정된 환자에게 시험대상자 동의를 위한 설명문을 통해 본 임상시험관련 정보를 제공하고 동의 시, 시험대상자로서 성명, 서명, 서명일을 자필 기재하여 문서 상의 동의서를 확보한다.

Screening No.(SN)은 동의 서명을 확보한 순서대로 부여하며 전체 다섯 자리로, 처음 두 숫자는 기관 번호를 의미하며, 이후 세 자리는 동의 서명 순서를 의미한다.

예를 들어 8 번째 기관에서 5 번째로 동의 서명이 확보된 시험대상자의 SN 는 'SN08-005'로 부여된다.

② 인구학적 정보 및 신장:

이니셜, 연령, 성별 등과 같은 인구학적 정보(위험군 평가 포함)를 비롯하여 신장 (소수점 둘째자리에서 반올림하여 소수점 첫째자리까지 기록, cm)을 확인한다.

③ 병력 및 치료력 조사 및 병용약물 확인:

동의 서명일을 기준으로 1 년 이내의 병력(과거 병력, 수술력 및 현재 병력) 및 4 주 이내의 모든 병용약물을 조사한다.

④ 활력징후(Vital Signs)

혈압, 맥박

⑤ 신체검사 (Physical exam) 및 체중

두부, 경부, 심장, 폐, 복부, 간, 피부, 사지, 기타 등을 검사하며, 체중을 (소수점 둘째자리에서 반올림하여 소수점 첫째자리까지 기록, kg)을 확인한다.

⑥ 임신반응검사

월경이 없는 상태가 12 개월 이상 지속된 폐경 또는 외과적 불임상태가 아닌 가임기 여성에 한하여 임신반응검사 (Urine hCG)를 시행하며 전체 임상시험 기간 동안 적절한 피임 방법을 사용한다.

⑦ 실험실 검사 – Central Lab 사용

식이 영향을 배제하기 위해 공복(최소 9 시간 이상의 금식) 상태에서 실험실 검사를 실시한다. 공복 상태가 아닌 경우 다시 방문하도록 하여 실험실 검사를 실시한다.

임상실험실적검사	세부항목
혈액학 검사	WBC with differential count (Neutrophil, Lymphocyte, Monocyte, Eosinophil, Basophil), RBC, Hb, Hct, Platelet)
혈액화학 검사	Ca, P, Glucose, HbA1c, BUN, Uric acid, Creatinine, Total Protein, Albumin, Total Bilirubin, Direct Bilirubin, AST, ALT, ALP, LDH, γ-GT(GGT), CPK(CK), Na, K, Cl
뇨 검사	Specific Gravity, PH, Protein(Albumin), Glucose, Ketone, Occult Blood, Urobilinogen, Nitrite
갑상선기능검사	TSH, Free T4
Lipid Parameters	LDL-C, HDL-C, TG, Total cholesterol, Apo-A1, Apo-B, Non-HDL-C/HDL-C ratio, Total Cholesterol/HDL-C ratio, LDL-C/HDL-C ratio, Apo-B/Apo-A1 ratio

* 재검 1 회를 허용한다.

⑧ 심전도 검사(ECG) - 연구자의 판단 하에 필요 시 실시한다.

12-lead ECG 사용

⑨ 선정/제외 기준 판정 및 지질저하제 복용 중지

적합하다고 판정된 시험대상자는 투여 중인 항고지혈증약제의 사용을 중단한다.

⑩ 생활습관교정(TLC) 교육:

임상시험 기간 동안 이상지질혈증 치료를 위한 생활습관교정 교육을 실시한다.

2) Visit 2: 치료 전 단계 (-1 week)

*Drug Naïve 이거나 스크리닝 검사 시점에서 Fibrate 제제 8 주 이상, Fibrate 외 제제 4 주 이상 복용 하지 않은 시험대상자의 경우 Wash out 을 진행하지 않고 선정/제외 기준을 만족하면 Visit 2 를 생략한다.

① 활력징후 (Vital Signs)

혈압, 맥박

② 신체검사 (Physical exam) 및 체중

두부, 경부, 심장, 폐, 복부, 간, 피부, 사지, 기타 등을 검사하며, 체중을 (소수점 둘째자리에서 반올림하여 소수점 첫째자리까지 기록, kg)을 확인한다.

③ 임신반응 검사

월경이 없는 상태가 12 개월 이상 지속된 폐경 또는 외과적 불임상태가 아닌 가임기

여성에 한하여 임신반응검사 (Urine hCG)를 시행하며 전체 임상시험 기간 동안 적절한 피임 방법을 사용한다.

④ 실험실검사 – Central Lab 사용

식이 영향을 배제하기 위해 공복(최소 9시간 이상의 금식) 상태에서 실시한다. 공복 상태가 아닌 경우 다시 방문하도록 하여 실험실 검사를 실시한다.

* 재검 1회를 허용한다. 재검을 하는 경우 치료 전 단계가 -2 week(D-14)까지 연장 될 수 있다.

⑤ 이상반응 및 병용약물 평가

지난 방문 이후 발생한 이상반응 및 병용약물에 대해 조사한다.

⑥ 생활습관교정(TLC) 교육:

지난 방문 이후 이상지질혈증 치료를 위한 생활습관교정을 잘 이행하고 있는지 확인한다.

⑦ 지질저하제 복용 중지

항고지혈증약제의 사용 중단을 지속한다.

⑧ 선정/제외기준 판정

3) Visit 3: 치료기 (0 week)

① 임신반응검사

월경이 없는 상태가 12 개월 이상 지속된 폐경 또는 외과적 불임상태가 아닌 가임기 여성에 한하여 임신반응검사 (Urine hCG)를 시행하며 전체 임상시험 기간 동안 적절한 피임 방법을 사용한다.

*Visit 3 만 Central lab 에서 제공한 임신반응검사 kit 를 사용하여 바로 결과를 확인하고, 근거문서에 기록한다.

② 배정 번호 (AN)부여

최종적으로 선정/제외기준에 적합한 시험대상자는 IWRS 무작위배정을 실시한다. 배정번호(AN)은 는 다섯 자리이며 처음 두 자리는 기관번호를 의미하고, 이후 세 자리는 무작위 배정 순서를 의미한다.

예를 들어 8 번째 기관에서 5 번째로 무작위 배정번호를 부여 받은 시험대상자의 AN 는 'AN08-005'로 부여된다.

③ 임상시험용의약품 처방

12 주간 복용할 수 있는 임상시험용의약품을 처방 (16 일 여유분 포함하여 100 일간 복용분)하게 되며, 이 때 시험대상자에게 임상시험용의약품 1 정을 1 일 1 회, 동일한 시간대에 복용하도록 권고하고 처방 당일부터 복용하도록 한다. 다음 방문(Visit 4)당일에는 임상시험용의약품을 복용하지 않으며, 임상시험용의약품을 복용한 경우 다시 방문하도록 하여 계획된 검사를 실시함을 교육한다. 복용하지 않은 임상시험용의약품을 지참하여 기관을 방문하도록 교육한다.

④ 이상반응 및 병용약물 평가

지난 방문 이후 발생한 이상반응 및 병용약물에 대해 조사한다.

⑤ 생활습관교정(TLC) 교육:

지난 방문 이후 이상지질혈증 치료를 위한 생활습관교정을 잘 이행하고 있는지 확인한다.

4) Visit 4: 종료방문 (12 week)

① 활력징후 (Vital Signs)

혈압, 맥박

② 신체검사 (Physical exam) 및 체중

두부, 경부, 심장, 폐, 복부, 간, 피부, 사지, 기타 등을 검사하며, 체중을 (소수점

둘째자리에서 반올림하여 소수점 첫째자리까지 기록, kg)을 확인한다.

③ 임신반응검사

월경이 없는 상태가 12 개월 이상 지속된 폐경 또는 외과적 불임상태가 아닌 가임기 여성에 한하여 임신반응검사 (Urine hCG)를 시행하며 전체 임상시험 기간 동안 적절한 피임 방법을 사용한다.

④ 실험실검사 – Central Lab 사용

식이 영향을 배제하기 위해 공복(최소 9 시간 이상의 금식) 상태에서 실시한다. 공복 상태가 아닌 경우 다시 방문하도록 하여 실험실 검사를 실시한다.

⑤ 심전도검사 (ECG) - 연구자의 판단 하에 필요 시 실시한다.

12-lead ECG 사용

⑥ 반납약 회수 및 순응도 평가

Visit 3 에 시험대상자에게 처방된 임상시험용의약품을 회수하고 순응도를 평가한다.

⑦ 이상반응 및 병용약물 평가

지난 방문 이후 발생한 이상반응 및 병용약물에 대해 조사한다.

⑧ 생활습관교정(TLC) 교육:

지난 방문 이후 이상지질혈증 치료를 위한 생활습관교정을 잘 이행하고 있는지 확인한다.

5) Unscheduled Visit

임상시험 진행 중 시험대상자는 필요한 경우, 예를 들어, 이상반응 등이 발생하였거나 이상반응 발생 여부가 의심될 경우 실시기관을 방문하여 필요한 검사를 시행할 수 있다. 이를 위해 시험자는 정기적 방문 시 시험대상자에게 이를 주지시키고 이상반응 발생 시 즉각적으로 연락할 수 있도록 한다. 계획되지 않은 방문은 전자증례기록서(e-CRF)에 기입한다.

10.2 유효성 평가변수와 평가

10.2.1 일차 유효성 평가변수(Primary Endpoint)

Baseline 으로부터 12 주 후의 LDL-C 의 변화율

10.2.2 이차 유효성 평가변수(Secondary Endpoint)

1) Lipid parameters 의 Baseline 으로부터 12 주 후의 변화율

   *Lipid parameters : HDL-C, TG, Total cholesterol, Apo-A1, Apo-B, Non-HDL-C/HDL-C ratio, Total Cholesterol/HDL-C ratio, LDL-C/HDL-C ratio, Apo-B/Apo-A1 ratio

2) HbA1c, Glucose 의 Baseline 으로부터 12 주 후의 변화량
3) 위험군별 LDL-C 목표 도달 비율 평가
4) 위험군별 Non-HDL-C 목표 도달 비율 평가

10.2.3 탐색적 평가 변수 (Exploratory Endpoint)

Baseline 으로부터 12 주 후의 LDL-C 의 비용-효과비

10.3 안전성 평가변수와 평가

10.3.1 이상반응

1) 임상시험 전반에 걸쳐 발생한 이상반응/약물이상반응/중대한 이상반응/중대한 약물이상반응 (AE/ADR/SAE/SADR)의 발현율
2) 예상하지 못한 이상반응/약물이상반응/중대한 이상반응/중대한 약물이상반응(Unexpected AE/ADR/SAE/SADR)의 발현율

10.3.2 활력징후

방문 시점 별로 평가한 활력징후 결과

10.3.3 실험실 검사

실험실검사 결과 임상적으로 의미 있는 비정상 비율

10.4 이상반응 보고

10.4.1 용어의 정의

1) "이상반응(Adverse Event, AE)"이란 임상시험용의약품을 투여한 시험대상자에게 발생한 모든 유해하고 의도하지 않은 증후(症候, sign, 실험실 실험 결과의 이상 등을 포함한다), 증상(症狀, symptom) 또는 질병을 말하며, 해당 임상시험용의약품과 반드시 인과관계를 가져야 하는 것은 아니다.

2) "약물이상반응(Adverse Drug Reaction, ADR)"이란 임상시험용의약품의 임의 용량에서 발생한 모든 유해하고 의도하지 않은 반응으로서 임상시험용의약품과의 인과관계를 부정할 수 없는 경우를 말한다.

3) "중대한 이상반응·약물이상반응(Serious AE·ADR)"이란 임상시험용의약품의 임의 용량에서 발생한 이상반응 또는 약물이상반응 중에서 다음 어느 하나에 해당하는 경우를 말한다.

가. 사망하거나 생명에 대한 위험이 발생한 경우

나. 입원할 필요가 있거나 입원 기간을 연장할 필요가 있는 경우

단, 아래와 같은 사유로 내원한 경우 SAE 로 간주하지 않는다.

- 진단 또는 기존에 갖고 있는 질환에 대해 계획된 수술을 받기 위한 입원 또는 입원기간의 연장
- 임상시험의 효과 측정을 위해 필요한 입원이나 입원기간의 연장
- 임상시험 대상질환에 대해 예정된 치료를 위한 입원이나 입원기간의 연장
- 응급실 방문 시, 내원 시간이 24 시간을 경과하지 않은 경우
- 건강검진을 위한 입원

다. 영구적이거나 중대한 장애 및 기능 저하를 가져온 경우

라. 태아에게 기형 또는 이상이 발생한 경우

마. 약물 의존성이나 남용의 발생 또는 혈액질환 등 그 밖에 의학적으로 중요한 상황이 발생되는사례

4) "예상하지 못한 약물이상반응(Unexpected Adverse Drug Reaction)"이란 임상시험자자료집 또는 의약품의 첨부문서 등 이용 가능한 의약품 관련 정보에 비추어 약물이상 반응의 양상이나 위해의 정도에서 차이가 나는 것을 말한다.

10.4.2 이상반응의 기록

임상시험 중 발생하는 모든 이상반응을 기록하는 것은 시험책임자와 담당자의 의무이다. 이상반응은 의학 진단 용어로 기록하여야 하며, 이것이 불가능한 경우 시험책임자 또는 담당자가 관찰하거나, 시험대상자가 보고한 증상 및 징후에 대한 용어를 기록하여야 한다.

10.4.3 심각한 정도의 평가

이상반응의 중증도는 임상적 판단에 근거하여 시험책임자 또는 담당자가 판단한다. 최대 강도(maximal intensity)를 기준으로 하며, 아래 분류 기준을 참고할 수 있다.

Grade	설명	
1	Mild (경증)	특별한 처치가 필요 없음.<br>증상이 없는 실험실적 검사 또는 방사선 결과의 이상 등
2	Moderate (중등증)	간단한 처치, 국소 처치, 비침습적 처치 등

3	Severe (중증)	입원 또는 침습적 처치 필요, 수혈, 치료적 내시경 또는 수술 등

10.4.4 약물과의 인과관계 평가

의약품 투여와 이상반응 발생의 연관 정도는 임상적 판단에 근거하여 시험책임자 또는 담당자가 판단한다. 이때, 의약품 등의 안전에 관한 규칙 별표 4의 3 의약품등 시판 후 안전관리기준에 따라, 의약품등과의 인과관계 평가기준을 다음과 같이 분류하며 "가능성 적음(Unlikely)"을 제외한 경우는 모두 의약품과 인과관계가 있다고 판단한다. 또한, "가능성 적음 (Unlikely)"인 경우 판단근거를 e-CRF에 입력한다.

인과관계		판단근거
1	확실함(Certain)	의약품등의 투여·사용과의 전후관계가 타당하고 다른 의약품이나 화학물질 또는 수반하는 질환으로 설명되지 아니하며, 그 의약품등의 투여중단시 임상적으로 타당한 반응을 보이고, 필요에 따른 그 의약품등의 재투여시, 약물학적 또는 현상학적으로 결정적인 경우
2	상당히 확실함 (Probable/likely)	의약품등의 투여·사용과의 시간적 관계가 합당하고 다른 의약품이나 화학물질 또는 수반하는 질환에 따른 것으로 보이지 아니하며, 그 의약품등의 투여중단시 임상적으로 합당한 반응을 보이는 경우(재투여 정보 없음)
3	가능함(Possible)	의약품등의 투여·사용과의 시간적 관계가 합당하나 다른 의약품이나 화학물질 또는 수반되는 질환에 따른 것으로도 설명되며, 그 의약품등의 투여중단에 관한 정보가 부족하거나 불명확한 경우
4	가능성 적음 (Unlikely)	의약품등의 투여·사용과 인과관계가 있을 것 같지 않은 일시적 사례이고, 다른 의약품이나 화학물질 또는 잠재된 질환에 따른 것으로도 타당한 설명이 가능한 경우
5	평가 곤란 (Conditional/Unclassified)	적정한 평가를 위해 더 많은 자료가 필요하거나 추가 자료를 검토 중인 경우
6	평가 불가 (Unassessable/Unclassifiable)	정보가 불충분하거나 상충되어 판단할 수 없고 이를 보완하거나 확인할 수 없는 경우
7	해당 없음(Not applicable)	

10.4.5 이상반응과 관련하여 취해진 조치

1) 임상시험용의약품과 관련된 조치

No.	설명
1	용량증량
2	용량유지
3	용량감량
4	투여중지
5	모름

6	해당없음

2) 그 외 조치

No.	설명
1	치료약물 병용 투여
2	비약물 치료
3	해당사항 없음
4	알 수 없음

10.4.6 이상반응의 경과

No.	설명
1	회복됨
2	회복중임
3	회복되지 않음
4	후유증을 동반한 회복
5	치명적 손상
6	모름
7	이상반응과의 연관성이 있을 수 있는 사망
8	이상반응과 관련이 없는 사망

10.4.7 중대한 이상반응 / 약물이상반응의 보고

본 시험 기간 중 임상시험책임자 및 담당자는 시험대상자의 안전에 만전을 기해야 하며, 중대한 이상반응 발생시에는 신속하고 적절한 조치를 취하여 이상반응을 최소화하여야 한다.

임상시험 중 중대한 이상반응 발생시 각 담당자의 의무는 다음과 같다.

1) 시험책임자의 의무

중대한 이상반응이 임상시험 기간 도중에 발생하는 경우, 각 실시기관 임상시험심사위원회의 표준작업지침서에 따라 임상시험심사위원회에 보고하여야 하며, 이상반응의 임상시험용의약품과의 관련 여부와 관계없이 시험책임자 또는 담당자는 24시간 이내, 혹은 늦어도 다음 근무일까지 ㈜종근당에 알려야 한다. 필요한 경우, 7일 이내에 상세한 내용이 포함된 추가 보고서를 작성하여 ㈜종근당 PV팀에 보고해야 한다.

ㅇ 전 화: 02-2194-0425

ㅇ 팩 스: 02-2194-0479

ㅇ 주 소: 서울시 서대문구 충정로 8

ㅇ 종근당 PV 담당자 e-mail: drugsafety2@ckdpharm.com

2) 시험담당자의 의무

시험담당자는 임상시험 실시 중 중대한 이상반응이 발생하는 경우 즉시 임상시험책임자에게 보고하여야 한다.

3)임상시험의뢰자의 의무

의뢰자는 시험자, 기관윤리심의위원회 및 한국의약품안전관리원장에게 임상시험에서 발생한 모든 이상반응을 다음의 구분에 따라 기한 내에 보고하여야 한다.

① 중대한 약물이상반응: 의뢰자가 해당 사실을 보고받거나 알게 된 날부터 15일 이내.

② ①항에 해당하지 않은 모든 중대한 이상반응: 매 분기 종료 후 1개월 이내

③ ①, ②항에 해당하지 않은 모든 이상반응: 결과보고서 제출일이 속한 분기의 종료 후 1개월 이내

10.4.8 이상반응의 추적 관찰

시험책임자 또는 담당자는 이상반응이 나타난 시험대상자에 대해 증상이 가라앉고 비정상적 임상검사치가 기준치로 회복되거나, 혹은 관찰된 변화에 대해 만족스러운 설명이 될 때까지 추적 관찰하여야 한다. 또한 이상반응의 진행 경과에 대하여 ㈜종근당의 담당자에게 보고하여야 한다.

10.4.9 임상시험 기간 중의 임신

임상시험 기간 중의 임신은 이상반응으로 간주하지 않으며, 합병증이 없는 선택적 유산(치료적 유산 제외) 또는 건강한 신생아의 정상 출산을 위한 입원 또한 중대한 이상반응으로 간주되지 않는다.

그러나 임상시험 기간 동안(임상시험용 의약품 투여 시점부터 마지막 투여 후 28 일 이내) 대상자(또는 배우자 또는 파트너)가 임신을 하게 되는 경우, 임신사실을 알게 된 후 24 시간 이내에 임신보고서 양식을 작성하여 임상시험 의뢰자에게 보고하여야 한다.

단, 임신정보 수집을 위해서는 임신정보 공유에 대한 동의서에 환자의 서명이 선행 되어야 한다. 대상자가 임신한 경우는, 시험 약물의 투여를 즉시 중단하고, 해당 대상자를 본 시험에서 탈락시킨다.

의뢰자는 대상자가 임상시험 참여를 중단하거나 임상시험을 종료 하더라도 이후 임산부와 태아의 경과에 대하여 출산 시까지 추적하고 문서화 하여야 한다.

11 자료분석 및 통계학적 고려사항

11.1 분석군

11.1.1 모든 분석 대상자군(Full Analysis Set, 이하 FAS)

무작위 배정된 모든 시험대상자를 분석에 포함시키는 배정 된 대로의 분석 원칙(The Intent-to-Treat Principle; ITT)을 가장 근접하고 완전하게 적용할 수 있는 분석대상 집단을 뜻한다. 따라서, 본 임상시험에 무작위 배정되어 1회 이상 임상시험용의약품을 복용하고, 임상시험용의약품 투여 후 최소한 1회 이상 유효성 평가를 시행한 시험대상자를 대상으로 한다.

11.1.2 계획서 순응 임상시험대상자군(Per Protocol Set)

계획서 순응 시험대상자 집단(이하, PP set)은 무작위 배정된 모든 시험대상자군 중에서 임상시험계획서를 중대하게 위반하였다고 고려되는 다음의 경우에 해당하는 시험대상자를 제외한다.

1) 임상시험계획서에 명시한 기간을 채우지 못하고 조기 종료한 시험대상자
2) 임상시험계획서를 중대하게 위반하였다고 판단되는 시험대상자
3) 전체 순응도가 80% 미만 또는 120% 초과인 시험대상자

11.1.3 안전성 분석 대상자군(Safety Analysis Set)

본 임상시험에 무작위 배정되고 임상시험용의약품 수령 후 1회 이상 복용한 시험대상자를 대상으로 한다.

11.2 통계분석방법

11.2.1 분석의 일반적 원칙

본 임상시험의 모든 통계분석은 유의수준 5% 하에서 양측검정을 실시한다. 유효성 자료에서 결측치가 발생한 경우 Observation Carried Forward(이하 LOCF) 방법을 적용하여 유효성 분석을 하고 안전성 자료의 분석은 LOCF 적용 없이 원자료를 그대로 분석한다.

11.2.2 인구학적 정보 및 기타 치료 전 특성

시험대상자의 인구학적 정보, 임상시험용의약품 투여 전 특성에 대하여 FA Set 을 대상으로 투여군별로 요약하고 분석한다. 연령 등의 연속형 자료는 평균, 표준편차, 중위수, 최솟값, 최댓값의 기술통계량을 제시하고 투여 군간 비교를 위해 독립 표본 t-검정(Independent Samples t-test)을 실시한다. 성별, 연령대와 같은 범주형 자료는 해당하는 시험대상자 수와 비율(%)을 제시하고 투여 군간 비교를 위해 카이제곱검정(Chi-square Test) 또는 피셔의 정확검정(Fisher's Exact Test)을 실시한다.

병용 약물은 임상시험용의약품 투여 이전에 투여 시작한 선행약물과 임상시험용의약품 투여 이후 투여 시작한 병용약물로 구분하여 WHO-ATC Index 에 따라 코딩함. WHO-ATC Index 1st level 에 따라 해당 시험대상자 수와 비율(%)을 제시하고 카이제곱검정(Chi-square test) 또는 피셔의 정확검정(Fisher's exact test)을 통하여 군간 비교를 실시한다.

또한, 병력은 MedDRA version20.0 을 사용하여 코딩한다. 신체기관계별 분류(SOC; System-Organ Class)에 따라 해당 시험대상자 수와 비율(%)을 제시하고 카이제곱검정(Chi-square test) 또는 피셔의 정확검정(Fisher's exact test)을 통하여 군간 비교를 실시한다.

11.2.3 유효성 자료의 분석(Statistical Analysis of Efficacy Data)

유효성 평가변수는 FA Set을 주 분석 대상, PP Set을 보조 분석 대상으로 하고 두 분석 결과의 경향이 다를 경우 그 원인을 확인해야 한다.

1) 1 차 유효성 평가 변수

   Baseline 대비 투여 12 주 후의 LDL-C 의 평균 변화율에 대하여 평균, 표준편차, 중위수, 최솟값, 최댓값의 기술통계량을 제시하고 시험군의 LDL-C 강하효과가 대조군의 LDL-C 강하효과에 비해 우월함을 보이기 위해 독립 표본 t-검정(Independent samples t-test) 또는 윌콕슨의 순위합 검정(Wilcoxon rank sum test)를 실시한다.

2) 2차 유효성 평가 변수

   ① Baseline 대비 투여 12주 후의 Lipid parameters의 평균 변화율에 대하여 평균, 표준편차, 중위수, 최솟값, 최댓값의 기술통계량을 제시하고 시험군과 대조군의 평균 변화율 비교를 위해 독립 표본 t-검정(Independent samples t-test) 또는 윌콕슨의 순위합 검정(Wilcoxon rank sum test)를 실시한다.

   * Lipid parameters : HDL-C, TG, Total cholesterol, Apo-A1, Apo-B, Non-HDL-C/HDL-C ratio, Total Cholesterol/HDL-C ratio, LDL-C/HDL-C ratio, Apo-B/Apo-A1 ratio

   ② Baseline 대비 투여 12주 후의 HbA1c, Glucose의 평균 변화량에 대하여 평균, 표준편차, 중위수, 최솟값, 최댓값의 기술통계량을 제시하고 시험군과 대조군의 평균 변화량 비교를 위해 독립 표본 t-검정(Independent samples t-test) 또는 윌콕슨의 순위합 검정(Wilcoxon rank sum test)를 실시한다.

   ③ 투여 12주 후 위험군별 LDL-C 도달 비율을 다음과 같이 설정하여 시험군과 대조군간 비교를 위해 카이제곱검정(Chi-square Test) 또는 피셔의 정확검정(Fisher's Exact Test)을 실시한다.

- 고위험군 LDL-C 도달: LDL-C 이 100 mg/dl 미만인 시험대상자의 비율(%)
- 초고위험군 LDL-C 도달: 초고위험군 LDL-C이 70 mg/dl 미만인 시험대상자의 비율(%)

④ 투여 12주 후 위험군별 Non-HDL-C 도달 비율을 다음과 같이 설정하여 시험군과 대조군간 비교를 위해 카이제곱검정(Chi-square Test) 또는 피셔의 정확검정(Fisher's Exact Test)을 실시한다.

- 고위험군 Non-HDL-C 도달: Non-HDL-C이 130 mg/dl 미만인 시험대상자의 비율(%)
- 초고위험군 Non-HDL-C 도달: Non-HDL-C이 100 mg/dl 미만인 시험대상자의 비율(%)

11.2.4 탐색적 평가변수의 분석(Statistical Analysis of Exploratory Data)

평균적 비용-효과비(ACER)를 아래와 같은 방법으로 산출하여

$$\mathrm{ACER} = \frac{mean(C_T - C_{N)}}{mean(E_T - E_N)}$$

ΔC: 시험군과 대조군의 평균비용 차이, ΔE: 시험군과 대조군의 평균 LDL – C 효과 차이

대조군에 비해 시험군의 증가된 효과 한 단위당 어느 정도의 비용이 추가 또는 절감되는지 탐색적으로 확인한다.

11.2.5 안전성 자료의 분석(Statistical Analysis of Safety Data)

안전성 평가변수는 Safety Analysis Set 을 대상으로 분석한다.

1) 이상반응

이상반응은 MedDRA version20.0 을 사용하여 코딩한다. 신체기관계별 분류(SOC; System-Organ Class)에 따라 해당 시험대상자 수와 발현율(%)을 제시하고 카이제곱검정(Chi-square test) 또는 피셔의 정확검정(Fisher's exact test)을 통하여 군간 비교를 실시한다. 시험대상자에게 발현한 이상반응은 약물이상반응(ADR), 중대한 이상반응(SAE), 중대한 약물이상반응(SADR)으로 구분하여 동일하게 분석한다.

또한, 허가사항 반영여부를 확인하여 예상하지 못한 이상반응(Unexpected AE)에 대한 신체기관계별 분류(SOC; System-Organ Class)와 우선순위 용어(PT; Preferred Terms)에 따라 해당 시험대상자 수와 발현율(%)을 제시하고 카이제곱검정(Chi-square test) 또는 피셔의 정확검정(Fisher's exact test)을 통하여 군간 비교를 실시함. 이상반응 분석과 동일하게 약물이상반응, 중대한 이상반응, 중대한 약물이상반응으로 구분하여 동일하게 분석한다.

2) 활력징후

활력징후 결과는 시점별 기술통계량을 제시하고 변화량에 대한 투여 군간 비교는 독립 표본 t-검정(Independent Samples t-test) 또는 윌콕슨 순위합 검정(Wilcoxon Rank Sum Test)을 통해 확인한다. 투여 군내 비교는 짝지은 표본 t-검정(Paired Samples t-test) 또는 윌콕슨 부호 순위 검정(Wilcoxon Signed Rank Test)을 통해 확인한다.

3) 실험실 검사

실험실 결과 임상적으로 의미 있는 비정상으로 판단된 시험대상자 수와 비율(%)을 투여 군별 제시하고 투여 전·후 비교는 맥니마 검정(McNemar Test), 투여 군간 비교는 카이제곱검정(Chi-square Test) 또는 피셔의 정확검정(Fisher's Exact Test)을 통해 확인한다.

11.3 분석시기 및 판정 기준

본 임상시험은 중간분석을 실시하지 않으며 임상시험을 종료한 후 통계분석을 수행한다.

11.4 시험대상자수 설정근거

본 임상시험은 원발성 이상지질혈증 환자에게 시험군(리피로우 20 mg)이 대조군(리피로우 10 mg)과 비교하여 12 주 후 LDL-C 변화율의 감소효과가 우월하다는 것을 증명하는 것이며, 이를 입증하기 위해 다음과 같은 가설을 설정하였다.

<가설>

$$H_0: \mu_t - \mu_c = 0 \quad vs. \quad H_1: \mu_t - \mu_c \neq 0$$

μ_t : 시험군의 baseline 대비 12 주 후 LDL-C 변화율

μ_c : 대조군의 baseline 대비 12 주 후 LDL-C 변화율

위 가설을 검정하기 위해 rosuvastatin FDA 허가자료를 검토해본 결과, 12 주 투여후의 LDL-C 변화율은 rosuvastatin 20 mg -50%(±13), rosuvastatin 10 mg -44%(±12)으로 군간 차이는 -6%으로 추정되었다.

% change, mean±sd	rosuvastatin 10 mg (n=44)	rosuvastatin 20 mg (n=44)
baseline	229±45	237±48
Week12	-44±12	-50±13

위 결과를 바탕으로 리피로우 20 mg과 10 mg 변화율 차이와 표준편차를 추정하고 아래 식을 이용하여 시험대상자 수를 산출하였다. 이때, 군간 차이는 -6, 표준편차를 13으로 추정하고 양측 유의수준 5%(단측 2.5%), 검정력 90%로 가정하였다. 산출한 시험대상자 수는 군당 99명이며 중도탈락률 20%를 고려 시 등재해야 할 시험대상자 수는 총 248명(124명X2군)이다.

$$n = \frac{2\times(Z_{1-\alpha/2}+Z_{1-\beta})^2\times\sigma^2}{\epsilon^2} = \frac{2\times(1.96+1.28)^2\times 13^2}{(-6)^2} = 2\times\frac{99}{(1-0.2)} \cong 248$$

$$\epsilon = \text{군간 차이}(-6)$$

$$\sigma^2 = \text{분산}(13^2)$$

12 자료관리

12.1 자료의 기록 및 수집

본 임상시험 중에 수집된 모든 자료는 임상시험 의뢰자가 제공한 전자 문서 형태의 증례기록서에 시험자가가 기록해야 한다. 자료가 누락된 경우에는 시험자가 타당한 설명을 붙여야 한다.

기록이 끝난 증례기록서는 임상시험 책임자가 최종 서명한다.

12.2 자료의 접근

임상시험 모니터요원, 점검을 실시하는 자, 심사위원회 및 식품의약품안전처장은 시험대상자의 비밀보장을 침해하지 않고 관련 규정이 정하는 범위 안에서 임상시험의 실시절차와 자료의 신뢰성을 검증하기 위해 자료를 직접 열람할 수 있다.

12.3 자료의 보호 및 보관

시험자는 본 시험의 이행에 관련된 기록이나 서류(예, 시험대상자 증례기록서, 시험대상자 동의서, 임상시험결과보고서, 그 외 관련정보서 등)를 품목허가일로부터(허가와 관계 없는 임상시험의 경우 임상시험 종료일 기준) 3 년간 보관하여야 한다.

13 윤리적 고려사항 및 행정적 절차

13.1 임상시험관리기준(KGCP)

본 임상시험을 실시함에 있어 KGCP 및 Helsinki 선언의 근본 정신을 준수하여 윤리적이고 과학적인 배려 하에 연구를 실시하도록 한다.

13.2 시험대상자 동의절차

시험대상자의 신상 보호를 위해 동의서 취득은 독립된 공간에서 진행한다. 시험담당자는 임상시험에 참여하는 시험대상자에게 사전에 시험의 성격, 범위, 예상되는 결과 등에 대하여 이해하기 쉽도록 설명하고, 시험대상자의 다양한 질문에 성실히 답변한다. 충분한 질의 응답 시간을 가진 후, 준비된 동의서 양식에 시험대상자 또는 시험대상자의 대리인이 서명을 하도록 하며, 시험담당자도 서명한다.

13.3 윤리준수

본 계획서 및 이에 대한 수정사항은 국내 규정에 따라 시험 실시의 정식 승인을 위하여 구성된 실시기관의 IRB 에 제출되며, 시험실시에 대한 IRB 의 결정은 시험자 및 의뢰자에게 시험 시작 전에 문서로 전달된다.

13.4 시험대상자 안전보호 대책

1) 치료 전 검사를 통해 시험대상자가 본 임상시험에 적절한 지를 엄격히 평가한다 .

2) 임상시험계획서에 따라 임상시험을 실시하고 시험기간 중 정기적인 검사와 검진을 통하여 이상반응 및 이상약물반응의 출현 여부와 그 정도를 평가하고 적절한 조치를 취한다 .

3) 임상시험실시기관은 본 시험계획서에 규정된 대로 임상시험이 적절히 진행될 수 있도록 임상시험에 필요한 설비와 전문인력을 갖추고 시험대상자의 안전보호에 만전을 기해야 한다 .

13.5 결과발표

본 임상시험의 결과는 학술잡지 또는 학회에 발표할 수 있다. 그러나 시험결과의 발표나 제출에 앞서 의뢰자는 발표내용을 검토할 권리가 있다.

13.6 환자기록 비밀 유지

임상시험의뢰자는 시험대상자에게 개인의 사생활에 대해 보호받을 권리를 제공해야 한다. 시험대상자 기록은 시험대상자의 코드 번호와 이니셜에 의해서만 확인 가능하도록 관리하고 임상시험 관련 자료는 잠금 장치가 있는 장소에 보관하도록 한다. 그러나 정부기관의 요청이나 임상시험의뢰자의 요청 등 경우에 따라 시험책임자는 임상시험과 관계되는 임상기록을 열람할 수 있도록 조치하고, 필요 시 복사본 등을 제공해야 한다. 서명을 받은 시험대상자 동의서는 임상시험 종료 시까지 시험책임자가 보관하며, 시험책임자는 시험대상자 번호 및 시험대상자명이 기록된 리스트를 작성하여 나중에 시험대상자 관련 기록을 찾을 수 있도록 관리한다.

13.7 품질관리 및 신뢰성 보증

13.7.1 품질관리

임상시험이 KGCP 에 따라 실시되고 국내외에서 등록 시 인정될 수 있도록 하기 위해 임상시험의뢰자 측에서 모니터링을 실시한다. 모니터링 시에는 증례기록이 완전하고 명확한지 확인하고 시험담당자의 입회 하에 대조 검토가 필요하며, 시험담당자는 임상시험용의약품의 조제 및 보관장소, 시험에 관련된 서류에 대해 임상시험 모니터 또는 본 업무의 위임자가 접근할 수 있도록 동의하고 협조해야 한다. 모든 미사용 임상시험용의약품은 임상시험이 종료된 후 회수한다.

13.7.2 신뢰성 보증

임상시험의뢰자는 신뢰성 보증의 일환으로 점검(Audit)을 실시할 수 있으며, 임상시험의 실시가 계획서, 표준작업지침서 및 관련 규정에 따라 이루어졌는지를 확인할 수 있다.

14 임상시험 의뢰자의 정보 및 시험책임자의 성명 및 직책

14.1 임상시험 의뢰자

1) 의뢰자명: ㈜종근당

2) 주소: 03742 서울시 서대문구 충정로 8

14.2 시험책임자의 성명 및 직책

14.2.1 시험조정자

고려대학교구로병원 순환기내과 김진원 교수

14.2.2 시험책임자

No.	소속	진료과	시험 책임자
1	고려대학교구로병원	순환기내과	김진원
2	고려대학교안산병원	순환기내과	송우혁
3	동아대학교병원	순환기내과	박종성
4	분당서울대학교병원	순환기내과	연태진
5	세종병원	심장내과	김지박
6	양산부산대학교병원	순환기내과	박용현
7	울산대학교병원	심장내과	김신재
8	원주세브란스기독병원	심장내과	안성균
9	인제대학교 일산백병원	순환기내과	도준형
10	명지병원	심장내과	조윤형

14.3 수탁기관(CRO, 분석기관, CRF, Central lab)

14.3.1 CRO

서울 CRO

14.3.2 분석기관

해당 없음

14.3.3 CRF 업체

CRScube

14.3.4 Central lab

서울의과학연구소(SCL)

15 참고문헌

1) National Cholesterol Education Program Adult Treatment Panel Ⅲ (NCEP ATP Ⅲ) guideline
2) 2013 ACC/AHA Guideline on the Treatment of Blood Cholesterol to Reduce Atherosclerotic Cardiovascular Risk in Adults
3) Clin Pharmacokinet 2003; 42(13): 1141-1160, Clinical Pharmacokinetics of Atorvastatin.
4) Cost-effectiveness of the Use of Statins in the Korean Population 2012;1(2):87-94, Journal of Lipid and Atherosclerosis
5) 리피로우 20 mg 허가사항 중 효능효과
6) 이상지질혈증 치료지침 (이상지질혈증 치료지침 제정위원회, 2015 년 제 3 판 전체본)
7) 고지혈증치료제에 대한 임상시험평가지침 (2008.02)
8) 고지혈증 치료제 임상시험 가이드라인 (2015.09)
9) Atorvastatin 사용상의 주의사항
